# Dissecting Causal Relationships Between Dietary Habits and Diverse Subtypes of Stroke: Mendelian Randomization Study

**DOI:** 10.3390/nu16203548

**Published:** 2024-10-19

**Authors:** Yan Cao, Fan Ye, Ling Zhang, Chuan Qin

**Affiliations:** 1 NHC Key Laboratory of Human Disease Comparative Medicine, Beijing Key Laboratory for Animal Models of Emerging and Remerging Infectious Diseases, Institute of Laboratory Animal Science, Chinese Academy of Medical Sciences and Comparative Medicine Center, Peking Union Medical College, Beijing 100730, China; caoyan961019@163.com (Y.C.); yefanvec@163.com (F.Y.); 2 Changping Laboratory, Beijing 102206, China

**Keywords:** dietary habits, stroke, adiposity, Mendelian randomization

## Abstract

Background: Understanding the causal relations between dietary habits and stroke is crucial for prioritizing public health interventions and developing effective health strategies. This study utilized Mendelian randomization (MR) analysis to examine the causal associations between 20 dietary habits and various stroke subtypes, aiming to identify potential mediators and evaluate the proportions of mediation. Methods: A two-sample MR analysis was conducted to examine the causal relationships between dietary habits and stroke incidence. Mediation analysis, two-step MR (TSMR), and multivariable MR (MVMR) were employed to identify potential mediators. Genetic data pertaining to dietary habits and stroke were obtained from extensive genome-wide association study (GWAS) consortia. The inverse variance-weighted (IVW) method served as the primary analytical approach, with the additional scrutiny of significant correlations conducted through the Egger regression, MR-Pleiotropy Residual Sum and Outlier (MR-PRESSO), and weighted median techniques. Results: Our analyses indicated that genetically predicted intakes of dried fruits, cheese, cereal, oily fish, and hot drink temperatures were protective against stroke, whereas higher intakes of lamb/mutton, poultry, and added salt significantly elevated stroke risk. Specifically, dried fruit consumption demonstrated a protective effect against total stroke (β = −0.009, *p* = 0.013), ischemic stroke (β = −0.475, *p* = 0.003), and small-vessel ischemic stroke (β = −0.682, *p* = 0.033) through reductions in BMI levels, accounting for mediated proportions of 3.2%, 17.1%, and 8.5%, respectively. Furthermore, cheese intake provided a protective effect against ischemic stroke (β = −0.275, *p* = 0.003) by decreasing BMI and increasing HDL-C levels, with mediated proportions of 30.5% and 6.5%. Together, BMI and HDL-C accounted for 34.9% of the beneficial effect of cheese intake on reducing the risk of ischemic stroke. In contrast, an increased salt intake exhibited a positive association with large-artery ischemic stroke (β = 0.432, *p* = 0.033) through BMI elevation, with a mediated proportion of 10.9%. Conclusions: Our findings provide compelling evidence supporting causal relationships between dietary habits and stroke subtypes, while identifying mediators and evaluating the proportions of mediation. Adhering to a low-calorie, nutrient-dense diet enriched with dried fruits, cheese, and cereal, along with reduced salt and poultry consumption, could potentially mitigate stroke risk.

## 1. Introduction

Stroke, a highly heterogeneous cardiovascular disease (CVD) with multiple underlying causes, stands as the second most common cause of mortality and the third most prevalent cause of disability globally, imposing a substantial economic burden on society [1,2]. This condition is primarily categorized into the following two types: ischemic stroke and hemorrhagic stroke, with ischemic stroke being the most prevalent form. It is commonly triggered by factors such as the atherosclerosis of large arteries, cardioembolism, and small-vessel occlusion. Stroke typically strikes suddenly, progresses rapidly, and can have devastating consequences, manifesting in symptoms such as limb paralysis, speech impediments, swallowing difficulties, cognitive impairment, and depression among affected patients. While it is essential to investigate the intricate pathophysiological mechanisms underlying stroke and enhance standardized treatment protocols, early prevention has emerged as a promising strategy for reducing stroke incidence and mitigating its associated complications. Among the various preventive approaches, dietary modifications have garnered increasing attention for their potential role in stroke prevention.

According to the findings from the 2019 Global Burden of Disease Study, approximately 87% of strokes were associated with modifiable risk factors, such as hyperglycemia, adiposity, hyperlipidemia, and hypertension, while 47% were linked to behavioral factors and lifestyle, including sedentary behavior, diet, and smoking [1]. Adopting a healthy lifestyle, particularly concerning dietary habits, is essential for general health and wellness. Embracing a balanced and nutritious diet, such as the Mediterranean diet, abundant in whole grains, nuts, vegetables, and fruits, could contribute to weight management, enhance immune function, and prevent disease [3,4]. Several studies have indicated that adherence to the Mediterranean diet is correlated with a reduced incidence of stroke, as confirmed by a network meta-analysis [5]. Furthermore, findings from the Lyon Diet Heart Study demonstrated that the Mediterranean diet was approximately twice as effective as simvastatin in secondary prevention. In primary prevention, a comparison of the consumption of low-fat foods and two variations of the Mediterranean diet—one fortified with mixed nuts and the other with olive oil—revealed that both versions significantly reduced cardiovascular events, with the nut-fortified diet exhibiting a 47% reduction over a five-year period [6]. In stark contrast, the Western diet, characterized by high levels of cholesterol, fat, and calories, was associated with a heightened risk of CVD [7,8,9,10,11]. Studies demonstrated that, with rising prosperity, the intake of eggs and meat escalated dramatically, while that of vegetables and fruits intake declined. This dietary shift was correlated with a 213% surge in coronary mortality and a 26.6% increase in stroke mortality from 2003 to 2013 [12]. This harmful Western dietary pattern might disrupt gut microbiota homeostasis, potentially leading to intestinal barrier dysfunction and the translocation of toxic bacterial byproducts into the bloodstream [13]. This cascade of events could trigger a low-grade inflammatory response, subsequently elevating stroke risk [11]. While previous studies have explored the relationship between diet and stroke, many of these investigations have inadequately controlled for confounders and reverse causality, impeding the establishment of definitive causal relationships. Additionally, some research has encountered challenges in assessing the correlation between specific stroke subtypes and dietary elements. Statistical power has often been insufficient to support stroke subgroup analysis, particularly due to the limited hemorrhagic stroke cases, rendering such results statistically insignificant. Moreover, accurately gauging the long-term dietary patterns of individuals, especially in observational studies, has proven challenging, as lifestyle habits frequently evolve over time due to various social, financial, or medical factors [14]. Furthermore, while dietary habits might affect susceptibility to stroke through various mechanisms, the ideal dietary practices for preventing stroke remain obscure [15].

To address these limitations, we employed Mendelian randomization (MR) analysis, utilizing genetic variations strongly associated with dietary habits as instrumental variables (IVs). This approach will facilitate a new perspective for reliably assessing the causal links between diet and various stroke subtypes [16]. Specifically, we performed a two-sample MR analysis to examine the causal links between 20 dietary habits and various stroke subtypes. Additionally, we conducted an MR mediation analysis to investigate potential intermediates and evaluate the proportions mediated, aiming to elucidate the preventive role of dietary habits in stroke prevention and provide recommendations for making the optimal food choices. Through this methodology, we aimed to overcome the limitations of previous studies and provide clearer evidence supporting the influence of diet on stroke risk. Our research not only strives to offer a scientific basis for public health policy formulation, but might also guide clinical practice, helping healthcare providers and nutritionists to develop more effective dietary intervention strategies in order to reduce the risk of stroke.

## 2. Materials and Methods

### 2.1. Study Design

**Figure 1:**
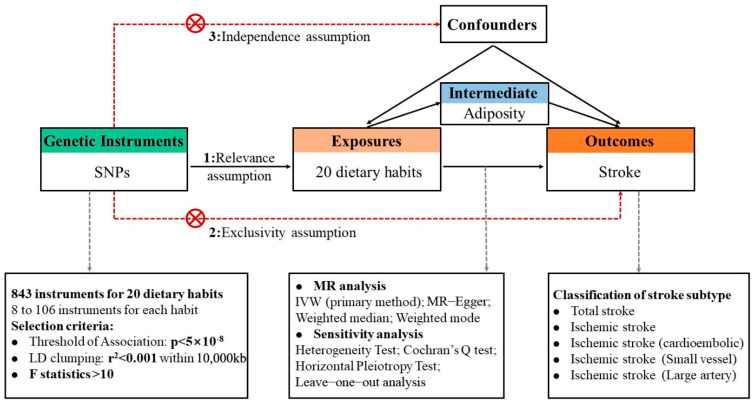
The workflow of the Mendelian randomization (MR) analysis focused on investigating the causal relationships between dietary habits and various subtypes of stroke. Initially, a two-sample MR analysis was conducted to assess the causal associations between 20 dietary habits and different types of stroke. This was followed by a two-step MR approach: Step 1 examined the impact of dietary habits on adiposity, while Step 2 elevated the influence of adiposity on different stroke subtypes. Finally, multivariate MR (MVMR) was employed to assess the mediation proportions.

The study design is illustrated in the diagram below, which emphasizes that the causal interpretations of MR estimates depended on the three following essential assumptions [17] (Figure 1): (1) the chosen IVs must be strongly associated with dietary habits; (2) these IVs should exhibit no association with any potential confounders; and (3) the IVs should influence the various subtypes of stroke exclusively through dietary habits, without involving other pathways. These principles were rigorously maintained throughout the entire study. To address potential biases that could compromise the reliability of our findings, we conducted an extensive series of downstream analyses. Specifically, we calculated F-statistics to confirm the validity of the IVs and employed Egger regression along with MR-Pleiotropy Residual Sum and Outlier (MR-PRESSO) to identify and adjust for horizontal pleiotropy and outliers. Additionally, we performed leave-one-out analysis to evaluate the influences of any predominant IVs [18,19,20].

### 2.2. Data Source

In this study, we identified 20 dietary habits as exposures based on previous research [21,22,23], including cooked vegetable intake, salad/raw vegetable intake, bread intake, lamb/mutton intake, fresh fruit intake, pork intake, beef intake, processed meat intake, cereal intake, water intake, cheese intake, hot drink temperature, coffee intake, dried fruit intake, non-oily fish intake, tea intake, oily fish intake, poultry intake, salt added to food, and frequency of alcohol consumption. Dietary information included both continuous variables, such as the average daily intake of coffee, and categorical variables, like how often poultry was consumed. We eliminated implausible responses during the data submission process. The specific contents of the questionnaire are presented (Table S1). Our primary outcome focused on the various subtypes of stroke, for which we obtained genome-wide association study (GWAS) data from a publicly available online database. These data included total stroke (sample size: 484,598; n_case: 6925; n_control: 477,673), ischemic stroke (sample size: 440,328; n_case: 34,217; n_control: 406,111), large-artery ischemic stroke (sample size: 150,765; n_case: 4373; n_control: 406,111), small-vessel ischemic stroke (sample size: 198,048; n_case: 5386; n_control: 192,662), and cardioembolic ischemic stroke (sample size: 211,763; n_case: 7193; n_control: 406,111). All stroke samples were derived from individuals of European ancestry. Additionally, we considered the following two indicators of adiposity: waist-to-hip ratio (WHR, sample size: 224,459) and body mass index (BMI, sample size: 454,884), along with two lipoprotein cholesterol metrics, high-density lipoprotein cholesterol (HDL-C, sample size: 357,810) and low-density lipoprotein cholesterol (LDL-C, sample size: 389,189), as potential intermediates. Summary-level data for adiposity traits were acquired from the Genetic Investigation of Anthropometric Traits (GIANT) Consortium for WHR and from the MRC Integrative Epidemiology Unit (MRC-IEU) Consortium for BMI. While individuals with the WHR trait were drawn from diverse populations, those with BMI, LDL-C, and HDL-C data had exclusively European ancestry. Presented below are detailed characteristics regarding the corresponding GWAS data sources (Tables S3 and S4).

### 2.3. Selection of IVs

For the MR analysis, we extracted single-nucleotide polymorphisms (SNPs) associated with the dietary habits and intermediates (*p* < 5 × 10^−8^), ensuring that these SNPs were independent of one another (r^2^ < 0.001 or distance > 10,000 kb). The instrumental strength was assessed using the F statistic, with an F statistic greater than 10 considered as adequate to mitigate weak instrumental bias [24]. The strength of each IV was calculated using the formulas R^2^ = (2β^2^ × EAF × (1 − EAF))/(2β^2^ × EAF × (1 − EAF) + 2N × EAF × (1 − EAF) × SE^2^) and F = (R^2^ × (N − 2))/(1 − R^2^), respectively. Among them, EAF represents the effect allele frequency, while SE and β denote the standard error and effect size of the SNP–antibodies association, respectively. N indicates the sample size [25].

### 2.4. Mediation Analysis Link “Dietary Habits–Adiposity–Stroke”

We employed the IVW method as the primary strategy for MR analysis. Mediation analysis, two-step MR (TSMR) [26], and multivariable MR (MVMR) [27] were utilized to estimate both the direct and indirect effects of dietary habits and adiposity on stroke. TSMR assumed no interaction between the exposures and intermediates. In addition to the basic effect estimate of dietary habits on stroke (β1) obtained from the two-sample MR analysis, the following two additional estimates were calculated: (1) the causal effect of the intermediates on stroke (β2) and (2) the causal effect of the exposures (the 20 different dietary habits analyzed in the primary MR analysis) on the intermediates (α). Furthermore, we performed MVMR as an alternative approach to validate the mediator roles identified in TSMR. In MVMR, we estimated the controlled direct effect as follows: the effect of intermediates on stroke while adjusting for dietary habits (β2*) and the effect of dietary habits on stroke while accounting for intermediates (β1*) [28]. The indirect effect, representing the causal influence of dietary habits on stroke through intermediates, was estimated using the product of coefficients method (α × β2*). Consequently, the proportion mediated was calculated as “indirect effect/total effect” ([α × β2*]/β1).

### 2.5. Sensitivity Analysis

To identify and minimize horizontal pleiotropy, the following three sensitivity analyses were performed: MR-Egger, the weighted median, and MR-PRESSO. Given its lower statistical power, MR-Egger primarily emphasizes the direction and magnitude of effects [29,30]. The weighted median provides a robust estimate when more than half of the SNPs are valid [31]. MR-PRESSO is capable of detecting outlier SNPs that may introduce pleiotropy and generating adjusted estimates after excluding these outliers [19]. Horizontal pleiotropy was evaluated utilizing the MR-Egger method, which employs weighted linear regression with an unconstrained intercept. This intercept indicates the average pleiotropic effect across genetic variants (the average direct effect of a variant with the outcome). If the intercept significantly deviates from zero (MR-Egger Intercept *p*-value < 0.05), it suggests the presence of horizontal pleiotropy. Additionally, heterogeneity was assessed using Cochrane’s Q test, where smaller *p*-values indicate a greater heterogeneity and a higher potential for directional pleiotropy. Leave-one-out analyses were also conducted to identify any outlier SNPs. All MR analyses were conducted in R (version 4.3.2) using the “TwoSampleMR” packages.

### 2.6. Ethical Approval and Consent to Participate

This study solely utilized publicly accessible GWAS data. Ethical approval and consent to participate are available in the original GWAS study.

## 3. Results

### 3.1. Genetic Instruments for 20 Different Dietary Habits

Twenty distinct dietary habits were considered as exposures in this study. Detailed information about the IVs employed for these dietary habits was provided (Table S2). The calculated F-statistic values for the identified single-nucleotide polymorphisms (SNPs) ranged from 29.74 to 811.85, indicating that the results were robust and less susceptible to biases associated with weak IVs. Based on this foundation, we proceeded to assess the causal relationships between 20 dietary habits and different subtypes of stroke.

### 3.2. Genetic Causality Between 20 Dietary Habits and Different Subtypes of Stroke

**Figure 2:**
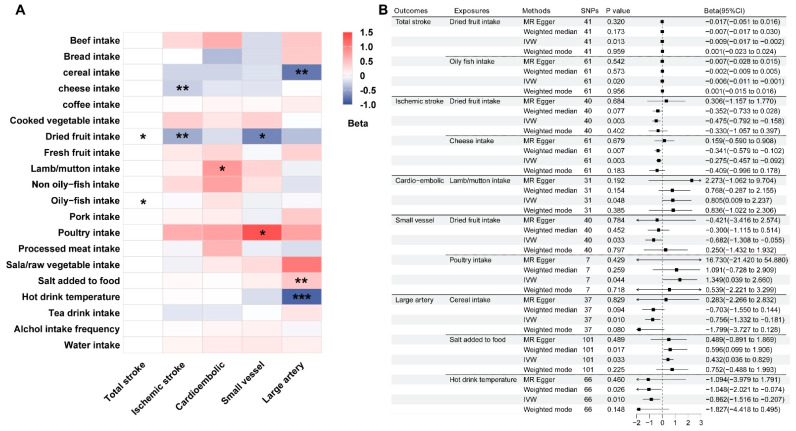
Heat map (**A**) and forest map (**B**) illustrated the statistically causal relationships between dietary habits and various subtypes of stroke, utilizing the inverse-variance weighting (IVW) approach. *: *p* < 0.05; **: *p* < 0.01; ***: *p* < 0.001.

The two-sample MR analysis was conducted to examine the causal relationships between dietary habits and various subtypes of stroke. The results demonstrated that the consumption of dried fresh fruit was significantly correlated with a reduced risk of total stroke (β = −0.009; 95%CI: −0.017 to −0.002; *p* = 0.013), ischemic stroke (β = −0.475; 95%CI: −0.792 to −0.158; *p* = 0.003), and small-vessel ischemic stroke (β = −0.682; 95%CI: −1.308 to −0.055; *p* = 0.033). Similarly, the consumption of oily fish and cheese intake were associated with a notable decrease in the risk of total stroke (β = −0.006; 95%CI: −0.011 to −0.0009; *p* = 0.020) and ischemic stroke (β = −0.275; 95%CI: −0.457 to −0.092; *p* = 0.003), respectively. Additionally, cereal intake (β = −0.756; 95%CI: −1.332 to −0.181; *p* = 0.010) and the consumption of hot beverages at an optimal temperature (β = −0.862; 95%CI: −1.516 to −0.207; *p* = 0.0098) were linked to a reduced risk of large-artery ischemic stroke. Conversely, the intake of lamb/mutton, poultry, and added salt significantly raised the risk of cardioembolic ischemic stroke (β = 0.805; 95%CI: 0.009 to 2.237; *p* = 0.048), small-vessel ischemic stroke (β = 1.349; 95%CI: 0.039 to 2.660; *p* = 0.044), and large-artery ischemic stroke (β = 0.432; 95%CI: 0.036 to 0.829; *p* = 0.033), respectively (Figure 2; Table S5). Other dietary habits showed no significant association with stroke risk. Scatter plots were generated to visually represent the primary results (Figure S1). While a low to moderate heterogeneity was observed in the associations between dietary habits and stroke, there was no indication of horizontal pleiotropy (MR-Egger Intercept *p* > 0.05). Furthermore, the leave-one-out estimates confirmed statistical significance (Figures S2 and S3).

### 3.3. Genetically Predicted Dietary Habits and Their Association with Potential Intermediators in Stroke Risk

Genetically predicted dietary habits demonstrated significant associations with several potential intermediators, including adiposity (measured by BMI and WHR) and lipoprotein cholesterol levels. Among the dietary habits associated with stroke risk, notable correlations were observed with these intermediators. Specifically, dried fruit intake exhibited a negative association with BMI (β = −0.395; 95%CI, −0.702 to −0.088; *p* = 0.012) and LDL-C levels (β = −0.105; 95%CI, −0.201 to −0.009; *p* = 0.032). Conversely, poultry intake and oily fish intake were associated with notable increases in BMI levels (β = 0.572; 95%CI, 0.042 to 1.101; *p* = 0.034) and HDL-C levels (β = 0.146; 95%CI, 0.007 to 0.286; *p* = 0.039), respectively. Furthermore, cereal intake was notably correlated with reductions in both BMI (β = −0.425; 95%CI, −0.587 to −0.262; *p* = 2.90 × 10^−7^) and WHR (β = −0.330; 95%CI, −0.484 to −0.176; *p* = 2.73 × 10^−5^). In contrast, salt added to food was linked to significant increases in both BMI (β = 0.162; 95%CI, 0.062 to 0.262; *p* = 2.00 × 10^−3^) and WHR (β = 0.175; 95%CI, 0.082 to 0.268; *p* = 2.00 × 10^−4^). Additionally, cheese intake could significantly reduce both BMI (β = −0.440; 95%CI, −0.584 to −0.296; *p* = 2.30 × 10^−9^) and WHR (β = −0.377; 95%CI, −0.494 to −0.259; *p* = 2.94 × 10^−10^), while also elevating HDL-C levels (β = 0.191; 95%CI, 0.001 to 0.381; *p* = 0.049) (Tables S6 and S8). Moreover, various intermediates were causally associated with different subtypes of stroke. Increased BMI levels were positively associated with total stroke (β = 0.0019; 95%CI, 0.0003 to 0.003; *p* = 0.017), ischemic stroke (β = 0.227; 95%CI, 0.166 to 0.314; *p* = 1.88 × 10^−13^), cardioembolic ischemic stroke (β = 0.132; 95%CI, 0.009 to 0.254; *p* = 0.035), small-vessel ischemic stroke (β = 0.191; 95%CI, 0.070 to 0.311; *p* = 1.90 × 10^−3^), and large-artery ischemic stroke (β = 0.299; 95%CI, 0.158 to 0.440; *p* = 3.17 × 10^−5^). WHR was also positively associated with ischemic stroke (β = 0.209; 95%CI, 0.066 to 0.352; *p* = 4.00 × 10^−3^) and small-vessel ischemic stroke (β = 0.531; 95%CI, 0.215 to 0.847; *p* = 1.00 × 10^−3^). Furthermore, LDL-C levels were positively associated with stroke (β = 0.001; 95%CI, 0.00 to 0.002; *p* = 0.05), ischemic stroke (β = 0.082; 95%CI, 0.011 to 0.153; *p* = 0.024), and large-artery ischemic stroke (β = 0.281; 95%CI, 0.291 to 0.434; *p* = 3.04 × 10^−4^). In contrast, HDL-C levels were negatively associated with ischemic stroke (β = −0.107; 95%CI, −0.166 to −0.049; *p* = 3.00 × 10^−4^), small-vessel ischemic stroke (β = −0.207; 95%CI, −0.308 to −0.107; *p* = 5.00 × 10^−5^), and large-artery ischemic stroke (β = −0.179; 95%CI, −0.298 to −0.059; *p* = 0.003). A moderate to high heterogeneity was observed; however, there was no evidence of horizontal pleiotropy, as indicated by the MR-Egger Intercept *p* value exceeding 0.05 (Tables S7 and S9).

**Figure 3:**
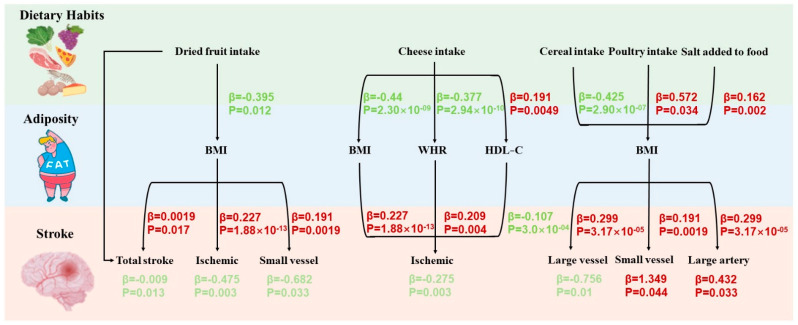
The two-step MR (TSMR) analysis revealed significant causal relationships between potential intermediates and dietary habits, as well as various subtypes of stroke. The diagram illustrated a TSMR mediation model depicting the pathway of “dietary habits–adiposity–stroke”. The beta values (β) in the diagram represent the estimated causal effects derived employing the IVW method, truncated at *p* < 0.05. Characters colored in red and green indicate negative and positive associations, respectively. BMI, body mass index; WHR, waist-to-hip ratio; and HDL-C, high-density lipoprotein cholesterol.

Additionally, we utilized TSMR analysis to examine the potential intermediates between dietary habits and stroke, as well as MVMR analysis to evaluate the proportions mediated. In the TSMR analysis, dried fruit intake exerted protective effects against various subtypes of stroke, particularly total stroke, ischemic stroke, and small-vessel ischemic stroke through the downregulation of BMI levels. Similarly, cheese intake appeared to confer protective effects against ischemic stroke by reducing both BMI and WHR levels while elevating HDL-C levels. Cereal intake contributed to a reduced risk of large-artery ischemic stroke through BMI reduction. Conversely, poultry intake and added salt were correlated with elevated risks of small-vessel ischemic stroke and large-artery ischemic stroke, respectively, by increasing BMI (Figure 3). In the MVMR analysis, we evaluated the mediation effects identified in the TSMR and calculated the indirect effects and proportions mediated. The results demonstrated indirect effects of BMI levels in the associations between dried fruit intake and total stroke, ischemic stroke, and small-vessel ischemic stroke, with mediated proportions of 3.2% (*p* = 3.46 × 10^−6^), 17.1% (*p* = 0.007), and 8.5% (*p* = 0.02), respectively. For cheese intake and ischemic stroke, the mediated proportion was 30.5% (*p* = 0.006), and for added salt and large-artery ischemic stroke, the mediated proportion was 10.9% (*p* = 0.012). Furthermore, HDL-C levels had an indirect effect on the relationship between cheese intake and ischemic stroke, demonstrating a mediated proportion of 6.5% (*p* = 0.047). Other associations did not reach statistical significance after adjustment (Table 1).

### 3.4. BMI and HDL-C Levels Were Particularly Involved in the Mediation of the Causal Association Between Cheese Intake and Ischemic Stroke

**Figure 4:**
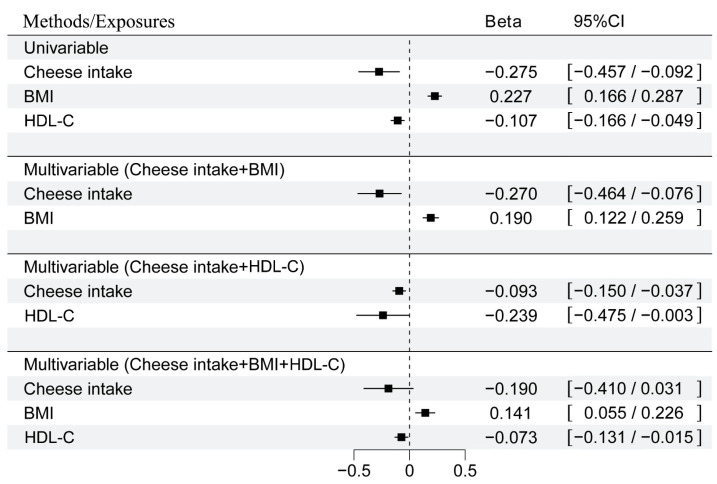
Effect of a one standard deviation (SD) increase in cheese intake on the odds of ischemic stroke in two-sample and multivariable models. Two-sample MR models provided effect estimates indicating that each SD increase in cheese intake exposure was associated with a reduction in the odds of ischemic stroke by over 0.275-fold. Multivariable models presented estimates that adjusted for additional factors; for instance, a one S.D. increase in cheese intake reduced the odds of ischemic stroke by 0.270-fold when accounting for BMI. This effect was further attenuated to 0.190-fold when both BMI and HDL-C levels were considered.

**Figure 5:**
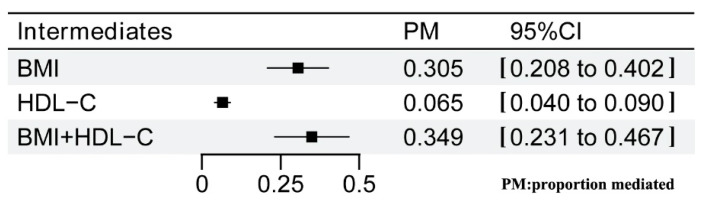
Estimates of the effect of cheese intake on ischemic stroke, delineated by each mediator and their combined influence. PM, proportion mediated.

In the MVMR analysis focusing on cheese intake, BMI levels, and ischemic stroke, the direct effect of cheese intake on ischemic stroke was estimated at β= −0.270 (95%CI: −0.464 to −0.076) after adjusting for BMI levels. Conversely, the direct effect of BMI on ischemic stroke was β = 0.190 (95%CI: 0.122 to 0.259) when controlling for cheese intake (Figure 4). The proportion of mediation attributed to BMI levels was found to be 30.5% (95%CI: 20.8% to 40.2%) (Figure 5). In another MVMR analysis examining the interplay between cheese intake, HDL-C levels, and ischemic stroke, the direct effect of cheese intake on ischemic stroke was observed at β = −0.093 (95%CI: −0.150 to −0.037) after accounting for HDL-C levels. Similarly, after accounting for cheese intake, the direct effect of HDL-C on ischemic stroke was assessed at β = −0.239 (95%CI: −0.475 to −0.003) (Figure 4). The mediation proportion attributed to HDL-C levels was 6.5% (95%CI: 4% to 9%) (Figure 5). Moreover, when both BMI and HDL-C levels were integrated into the MVMR analysis, the effect sizes for cheese intake (β = −0.190; 95%CI: −0.410 to 0.031), BMI levels (β = 0.141; 95%CI: 0.055 to 0.226), and HDL-C levels (β = −0.073; 95%CI: −0.131 to −0.015) were notably attenuated (Figure 5). Collectively, BMI and HDL-C levels accounted for a mediation proportion of 34.9% (95%CI: 23.1% to 46.7%) of the effect of cheese intake on ischemic stroke (Figure 5). Undeniably, there were inherent challenges in fully accounting for all potential confounding factors. Careful consideration was required when interpreting these results and further studies—potentially involving longitudinal data or randomized controlled designs—are necessary for robust causal validation.

## 4. Discussion

In this study, we conducted a comprehensive MR analysis to elucidate the causal relationships between dietary habits and the incidence of different stroke subtypes, along with investigating potential mediating pathways. Our two-sample MR analysis revealed significant findings, as follows: the consumption of fresh fruits and oily fish was associated with a reduced risk of stroke. Specifically, cheese intake and fresh fruit intake were pivotal in decreasing the incidence of ischemic stroke, while lamb consumption was linked to a higher risk of cardioembolic ischemic stroke. Further analysis demonstrated that fresh fruit intake lowered the risk of small-vessel ischemic stroke, whereas poultry consumption increased this risk. Additionally, cereal and hot beverage intake significantly reduced the risk of large-vessel ischemic stroke, contrasting with the adverse impact of a high salt intake. Mediation analysis highlighted that obesity and dyslipidemia, influenced by dietary choices, played critical roles in stroke development. Notably, cheese consumption appeared to lower BMI and elevate HDL-C levels, suggesting a promising strategy for reducing ischemic stroke risk. These findings underscore the importance of specific dietary patterns in stroke prevention and provide valuable evidence to inform public health policies and individual dietary choices.

The relationships between dietary habits and stroke were multifaceted, with different dietary components exerting diverse effects on different subtypes of stroke. Research findings have indicated a correlation between an increased consumption of dried fruit and a decreased risk of ischemic stroke and small-vessel ischemic stroke. Specifically, the risk of ischemic stroke decreased by 54.53% and that of small-vessel stroke risk decreased by 65.01% with a daily intake of 1.8 g more dried fruit [32], which is consistent with our findings [33]. Dried fresh fruits are abundant in vitamins, minerals, antioxidants, and dietary fiber, which, together, have the potential to enhance endothelial function, mitigate blood clot formation, and decelerate the progression of atherosclerosis, consequently lowering the incidence of stroke [34]. These studies indicated that the ingestion of dietary fiber and antioxidants could significantly contribute to cardiovascular health. Moreover, the intake of oily fish, cheese, cereal, and hot beverages at an appropriate temperature was correlated with a reduced incidence of total stroke, large-artery ischemic stroke, and ischemic stroke. Oily fish is abundant in omega-3 fatty acids, which have the potential to diminish platelet aggregation, inhibit inflammatory processes, and enhance endothelial function, thereby exerting a protective effect against stroke [35,36,37]. Previous studies indicated that a minimal fish intake of 175 g (equivalent to around two servings) weekly was associated with a lower risk of major CVD among patients with a history of CVD, but not in general populations [38]. Consequently, it was recommended that individuals with CVD moderately increased their intake of oily fish. Dairy products such as cheese can serve as valuable sources of calcium, vitamins, and probiotics that have also been proven to lower blood pressure, regulate lipid metabolism, and optimize the gut microbiota composition, which could further contribute to a reduced risk of stroke [39]. Numerous studies have demonstrated that cheese consumption could significantly reduce the risk of stroke [40,41,42]. However, the relationship between cheese intake and its benefits follows a “U-shaped” pattern, rather than a linear one. This indicates that individuals who consume cheese in moderation (averaging 40 g per day) exhibit the lowest risk of stroke. Interestingly, research has revealed that cheese consumption significantly elevates levels of HDL-C, thereby contributing to a protective effect against stroke, a finding that is highly consistent with our experimental results. Conversely, an excessive consumption of mutton, poultry, and salt was linked to an increased risk of cardioembolic ischemic stroke, small-vessel ischemic stroke, and large-artery ischemic stroke. The study demonstrated that diet-related inflammation might significantly elevate the risk of stroke, with individuals exhibiting the highest dietary inflammation index facing an 87% increased risk compared to those with the lowest index [11]. The consumption of high-fat and high-cholesterol meat and poultry could trigger inflammatory responses, exacerbate atherosclerosis, and elevate the risk of vascular occlusion and thrombosis [43,44]. Excessive salt intake was associated with hypertension, fluid retention, and arterial stiffness, all of which significantly contribute to stroke risk [45,46]. Importantly, the study did not consider inflammation as an intermediary factor, which limited our understanding of its mediating role in the relationship between dietary habits and stroke incidence. Future research should aim to explore this potential intermediate to better elucidate the complex interplay between dietary choices, inflammation, and stroke risk.

Through MR mediation analysis, we further investigated the potential intermediates in the causal relationships between dietary patterns and stroke subtypes, while assessing the proportions mediated by adiposity and lipoprotein cholesterol levels. Our findings demonstrated that these factors play crucial roles in determining stroke risk. Specifically, dried fresh fruits exerted protective effects against total stroke, ischemic stroke, and small-vessel ischemic stroke, primarily by lowering BMI levels. Antioxidant-rich fruits and vegetables play an important role in enhancing cardiovascular health, potentially by reducing inflammation and improving vascular function [47]. Similarly, cereal intake was also associated with protective effects against large-artery ischemic stroke. Previous studies have indicated that increasing dietary fiber could improve lipid profiles, especially by enhancing HDL-C levels, thereby reducing the risk of cardiovascular events [48]. However, this study showed no significant causal relationship between cereal intake and HDL-C levels. Previous studies might have been influenced by confounding factors, such as participants’ lifestyle, dietary habits, and socioeconomic status, which can partially influence HDL-C levels, resulting in biased results. Moreover, the varieties of cereal available were extensive, and different types of cereal differ significantly in their nutritional composition and metabolic effects. Failure to adequately control for the intake of different types of cereal might lead to exaggerations about the impact of cereal intake on HDL-C. Furthermore, cheese intake was linked to a decreased risk of ischemic stroke through a lower BMI and WHR. Some studies have suggested that certain fatty acid components in dairy products might contribute to weight loss while reducing overall energy intake by enhancing satiety. Conversely, a high consumption of poultry and salt was found to elevate the risk of large-artery ischemic stroke and small-vessel ischemic stroke by elevating BMI levels. A high-salt diet could lead to hypertension, thereby increasing the incidence of CVD [49]. Given the already established causal relationship between hypertension and CVD, our findings somewhat validated the current consensus regarding the association between diet and cardiovascular health. Previous research has demonstrated that, after adjusting for confounders such as education level, smoking, alcohol consumption, and physical activity, adiposity is closely associated with stroke [50]. Adiposity influences cardiovascular pathophysiology through various mechanisms. Anatomically, obesity leads to excessive fat distribution—especially visceral fat—which is closely associated with the development of CVD. For example, fatty tissue surrounding the kidneys can activate the renin–angiotensin–aldosterone system (RAAS), leading to elevated blood pressure levels. Metabolically, the cytokines secreted by visceral fat, including leptin, adiponectin, and TNF-α, can incite inflammatory responses and cause endothelial dysfunction, thereby facilitating atherosclerosis and heightening the risk of CVD. Furthermore, adipose tissue serves as a reservoir for immune cells, including macrophages and lymphocytes, with the macrophages in adipose tissue often polarizing towards the pro-inflammatory M1 phenotype. This results in the release of cytokines that promote endothelial dysfunction and thrombosis. Additionally, the excessive infiltration of immune cells into adipose tissue can trigger systemic inflammatory responses, further exacerbating CVD [51].

Lipoprotein cholesterol levels were crucial factors mediating the associations between dietary habits and the risk of stroke. Dysfunction of the adipose tissue is frequently accompanied by dyslipidemia, a crucial hallmark of CVD. The excessive release of free fatty acids and inflammatory mediators from adipocytes can impair the lipid metabolism, leading to heightened triglyceride production in the liver, an impaired clearance of lipids, and elevated plasma triglyceride and LDL-C levels, while decreasing HDL-C levels. These lipid imbalances contribute to the pathogenesis of atherosclerosis, thereby increasing the risk of stroke [52,53,54,55]. Our findings revealed that the intake of oily fish, cheese, and appropriately consumed hot drinks exhibited positive associations with HDL-C levels. This aligns with previous studies that have highlighted the beneficial effects of omega-3 fatty acids from oily fish on lipid profiles, which significantly increased HDL-C levels while simultaneously decreasing triglyceride levels [56]. In contrast, our research indicated that mutton intake was linked to increased LDL-C levels. Red meat consumption, particularly processed forms, was linked to higher LDL-C levels due to their increased saturated fat content [57]. Moreover, dried fresh fruit intake was shown to noticeably decrease LDL-C levels. Collectively, cheese intake and the consumption of optimal hot drink temperatures exerted protective effects against ischemic stroke and large-artery ischemic stroke through elevating HDL levels. Cheese, being rich in minerals such as calcium, might protect against stroke by modulating fatty acid absorption and cholesterol metabolism [58,59,60]. Furthermore, the probiotics in cheese can suppress inflammation and the immune response by modulating the gut microbiota composition and influencing bile acid reabsorption, further benefiting cardiovascular health [61,62]. However, some studies have revealed mixed results regarding the health effects of dairy consumption, suggesting that high-fat dairy products can exacerbate cardiovascular risks due to their saturated fat content. For instance, previous studies have posed that high-fat dairy intake might be detrimental, raising LDL-C levels in some populations [63]. These discrepancies might arise from differences in dietary patterns, genetic predispositions, and the specific populations studied. In summary, our research supports the idea that certain dietary components, including oily fish, cheese, and dried fruits, can positively influence lipid profiles and reduce the risk of stroke. Future studies should continue to investigate these associations, considering varying dietary contexts and the interplay of macronutrients to elucidate their mechanisms further and inform dietary guidelines aimed at stroke prevention.

The complex interplay between dietary habits and adiposity, along with lipoprotein cholesterol, further influences the risk of various stroke subtypes. Diets characterized by high calorie consumption yet a low nutrient density—particularly those enriched in saturated fats, refined sugars, and processed foods—can contribute to increased adiposity, insulin resistance, and dyslipidemia, subsequently exacerbating stroke risk [64]. Conversely, a low-calorie, nutrient-dense diet enriched in whole grains, vegetables, fruits, and lean proteins can provide a protective effect against stroke. Such diets are enriched in essential nutrients, antioxidants, and anti-inflammatory compounds that counteract adipose tissue dysfunction and optimize lipid distribution. Understanding these intricate interactions between dietary habits and stroke is paramount for developing effective prevention, early detection, and management strategies against stroke. Targeted interventions aimed at alleviating adiposity and promoting healthy dietary patterns, alongside lifestyle modifications, are paramount for mitigating the burden of stroke. Given the rising global incidence of stroke, it is imperative to clarify the mechanistic links between adiposity, metabolic dysfunction, and cardiovascular pathology. This understanding would lay a robust theoretical foundation for personalized prevention and treatment strategies that cater to individual risk factors and metabolic profiles. By tailoring interventions to address the unique needs of diverse populations, we can enhance their efficacy and ensure that they are better suited for real-world application. This approach will not only empower healthcare providers with actionable insights, but also encourage individuals to adopt healthier lifestyles, ultimately contributing to a reduction in stroke incidence and its associated complications.

The strengths of this study primarily originate from its utilization of data derived from larger-scale research, employing two-sample MR analysis in conjunction with MVMR analysis. This methodological framework allowed us to investigate the potential intermediate pathways linking dietary habits, adiposity, and stroke risk. By levering genetic variants as IVs, we provided insights into causal relationships while mitigating concerns related to confounding factors and reverse causation. This rigorous approach contributes significantly to the credibility of our findings and their applicability in clinical contexts. However, several limitations warrant careful consideration when interpreting our findings. (1) The validity of our assumptions could have been compromised by horizontal pleiotropy, whereby genetic variants affected outcomes through pathways unrelated to the exposure being examined. This could potentially lead to biased estimates and undermine the reliability of our conclusions. To address these, we conducted a series of sensitivity analyses, including MR-PRESSO, the intercept test in MR-Egger regression, a heterogeneity test, and a leave-one-out analysis. These measures consistently corroborated our findings, reinforcing the robustness of our results and supporting the validity of our methodological approach. (2) Another limitation was that the GWAS analyzed predominantly involved individuals of European descent. This demographic limitation might restrict the generalizability of our results to other ethnic groups, as the genetic and environmental factors influencing stroke risk might differ significantly across populations. Further research should seek to incorporate more diverse cohorts to enhance the applicability of findings across different ethnic backgrounds. (3) The original study lacked detailed data, which prevented us from conducting further nonlinear regression analyses and subgroup analyses that could provide additional insights into the intricate relationships between dietary factors, adiposity, and stroke risk. Such analyses could offer a more nuanced understanding of how specific dietary components interact with various metabolic factors to influence stroke outcomes. Future research should focus on aggregating data across multiple studies or conducting meta-analyses to better assess the implications of our findings across different stroke types.

In conclusion, while this study provides valuable insights into the role of dietary patterns and adiposity in stroke risk, acknowledging its limitations is essential for the interpretation of our findings. Future research should aim to address these gaps through comprehensive datasets and diverse population samples, ultimately contributing to more effective and personalized stroke prevention strategies.

## 5. Conclusions

This study elucidated a clear causal relationship between 20 distinct dietary habits and various subtypes of stroke, revealing the roles of potential intermediates and quantitatively assessing their mediation proportions. These findings provide critical insights that significantly advanced our understanding of dietary influences on stroke risk, offering actionable dietary recommendations aimed at enhancing stroke prevention strategies. Moreover, the results underscore the urgent need for further investigation into the underlying biological and physiological mechanisms linking specific dietary habits to stroke risk. By elucidating these connections, we can develop targeted interventions that not only effectively reduce stroke incidence, but also enhance public health outcomes. This research lays the groundwork for future initiatives that can tailor prevention strategies to individual risk profiles, ultimately contributing to more effective stroke management and improved health across populations.

## Figures and Tables

**Table 1:** The MVMR analysis examined the causal relationships between dietary habits, potential intermediates, and various subtypes of stroke. Beta (β), standard errors (SE), and *p*-values were derived from the MVMR analysis. The values β1* and β2* indicate the controlled direct effects of each dietary habit–intermediate pair on stroke, adjusted for one another. The parameter α denotes the effect of dietary habits on the intermediates, while the indirect effect (α × β2*) reflects the influence of dietary habits on stroke through the associated intermediates. The β1 value reflects the overall effect of dietary habits on stroke, as determined by the two-sample MR approach. The mediated proportion is calculated as the ratio of indirect effects to the total effect.

Outcomes	Exposures	Intermediators	Direct Effect (β1* ± SE)	Direct Effect (β2* ± SE)	Indirect Effect (α × β2* ± SE)	*p* Value	Proportion Mediated (α × β2*/β1)
Total stroke	Dried fruit intake	BMI	−0.016 ± 0.004	0.0007 ± 0.008	−0.0003 ± 0.0001	3.46 × 10^−6^	0.032
Ischemic stroke	Dried fruit intake	BMI	−0.383 ± 0.142	0.206 ± 0.033	−0.081 ± 0.005	0.007	0.171
	Cheese intake	BMI	−0.270 ± 0.099	0.190 ± 0.035	−0.084 ± 0.003	0.006	0.305
	Cheese intake	WHR	−0.107 ± 0.124	0.205 ± 0.084	−0.077 ± 0.005	0.385	0.280
	Cheese intake	HDL-C	−0.239 ± 0.120	−0.093 ± 0.029	−0.018 ± 0.003	0.047	0.065
Small vessel	Dried fruit intake	BMI	−0.661 ± 0.285	0.147 ± 0.066	−0.058 ± 0.01	0.020	0.085
	Poultry intake	BMI	0.597 ± 0.389	0.130 ± 0.075	0.074 ± 0.020	0.125	0.055
Large artery	Cereal intake	BMI	−0.582 ± 0.325	0.261 ± 0.079	−0.111 ± 0.007	0.073	0.147
	Salt added to food	BMI	0.561 ± 0.222	0.290 ± 0.075	0.047 ± 0.004	0.012	0.109

## Data Availability

The original contributions presented in the study are included in the article/Supplementary Material, further inquiries can be directed to the corresponding author.

## Supplementary Materials

The following supporting information can be downloaded at: https://www.mdpi.com/article/10.3390/nu16203548/s1, Figure S1: Scatter plots depicting the significant associations between dietary habits and stroke; Figure S2: Funnel plots depicting the significant associations between dietary habits and stroke; Figure S3: Leave−one−out plots depicting the significant associations between dietary habits and stroke; Table S1: Questionnaire survey on 20 dietary habits; Table S2: Significant SNP associated with exposures; Table S3: Detailed information of exposures dataset; Table S4: Detailed information of intermediators and outcomes dataset; Table S5: Detailed information on the significant causal relationship between dietary habits and stroke; Table S6: Detailed information on the significant causal relationship between dietary habits and obesity; Table S7: Detailed information on the significant causal relationship between obesity and stroke; Table S8: Detailed information on the significant causal relationship between dietary habits and lipoproteins; Table S9: Detailed information on the significant causal relationship between lipoproteins and stroke.
