# Supplementary material for: Dissecting Causal Relationships Between Dietary Habits and Diverse Subtypes of Stroke: Mendelian Randomization Study
Source: Nutrients. 2024 Oct 19;16(20):3548. doi: 10.3390/nu16203548 (PMC11510269; doi:10.3390/nu16203548)

Supplementary material

Contents

Supplementary Tables

Supplementary Table 1. Questionnaire survey on 20 dietary habits.
Supplementary Table 2. Significant SNP associated with exposures.
Supplementary Table 3. Detailed information of exposures dataset.
Supplementary Table 4. Detailed information of intermediators and outcomes dataset.
Supplementary Table 5. Detailed information on the significant causal relationship between dietary habits and stroke.
Supplementary Table 6. Detailed information on the significant causal relationship between dietary habits and obesity.
Supplementary Table 7. Detailed information on the significant causal relationship between obesity and stroke.
Supplementary Table 8. Detailed information on the significant causal relationship between dietary habits and lipoproteins.
Supplementary Table 9. Detailed information on the significant causal relationship between lipoproteins and stroke.

Supplementary Figures

Supplementary Figure 1. Scatter plots depicting the significant associations between dietary habits and stroke.
Supplementary Figure 2. Funnel plots depicting the significant associations between dietary habits and stroke.
Supplementary Figure 3. Leave-one-out plots depicting the significant associations between dietary habits and stroke.

Supplementary Table 1. Questionnaire survey on 20 dietary habits.

	Category	Question	Options
Beef intake	Categorical(single)	How often do you eat beef? (Do not count processed meats)	Options: never, less than once a week, once a week, 2-4 times a week, 5-6 times a week, once or more daily, do not know, prefer not to answer
Bread intake	Integer, slices/week	How many slices of bread do you eat each WEEK? For other types of bread:<br>- one bread roll = 2 slices<br>- one pitta bread = 2 slices	-
Cereal intake	Integer, bowls/week	How many bowls of cereal do you eat a WEEK?	-
Cheese intake	Categorical(single)	How often do you eat cheese? (Include cheese in pizzas, quiches, cheese sauce etc)	Options: never, less than once a week, once a week, 2-4 times a week, 5-6 times a week, once or more daily, do not know, prefer not to answer
Coffee intake	Integer, cups/day	How many cups of coffee do you drink each DAY? (Include decaffeinated coffee)	-
Cooked vegetable intake	Integer, tablespoons/day	On average how many heaped tablespoons of COOKED vegetables would you eat per DAY? (Do not include potatoes; put '0' if you do not eat any)	-
Dried fruit intake	Integer, pieces/day	About how many pieces of DRIED fruit would you eat per DAY? (Count one prune, one dried apricot, 10 raisins as one piece; put '0' if you do not eat any)	-
Fresh fruit intake	Integer, pieces/day	About how many pieces of FRESH fruit would you eat per DAY? (Count one apple, one banana, 10 grapes as one piece; put '0' if you do not eat any)	-
Lamb/mutton intake	Categorical(single)	How often do you eat lamb/mutton? (Do not count processed meats)	Options: never, less than once a week, once a week, 2-4 times a week, 5-6 times a week, once or more daily, do not know, prefer not to answer
Oily fish intake	Categorical(single)	How often do you eat oily fish? (e.g. sardines, salmon, mackerel, herring) Oily fish include: Salmon Anchovies, Trout Swordfish, Mackerel Bloater, Herring Cacha, Sardines Carp, Pilchards Hilsa,	Options: never, less than once a week, once a week, 2-4 times a week, 5-6 times a week, once or more daily, do not know, prefer not to answer

		Kipper Jack fish, Eel Katla, Whitebait Orange roughy, Tuna (fresh only) Pangas, Sprats	
Non-oily fish intake	Categorical(single)	How often do you eat other types of fish? (e.g. cod, tinned tuna, haddock)	Options: never, less than once a week, once a week, 2-4 times a week, 5-6 times a week, once or more daily, do not know, prefer not to answer
Pork intake	Categorical(single)	How often do you eat pork? (Do not count processed meats such as bacon or ham)	Options: never, less than once a week, once a week, 2-4 times a week, 5-6 times a week, once or more daily, do not know, prefer not to answer
Poultry intake	Categorical(single)	How often do you eat chicken, turkey or other poultry? (Do not count processed meats)	Options: never, less than once a week, once a week, 2-4 times a week, 5-6 times a week, once or more daily, do not know, prefer not to answer
Processed meat intake	Categorical(single)	How often do you eat processed meats (such as bacon, ham, sausages, meat pies, kebabs, burgers, chicken nuggets)?	Options: never, less than once a week, once a week, 2-4 times a week, 5-6 times a week, once or more daily, do not know, prefer not to answer
Salad/raw vegetable intake	Integer, tablespoons/day	On average how many heaped tablespoons of SALAD or RAW vegetables would you eat per DAY? (Include lettuce, tomato in sandwiches; put '0' if you do not eat any)	-
Salt added to food	Categorical(single)	Do you add salt to your food? (Do not include salt used in cooking)	Options: never/ rarely, sometimes, usually, always, prefer not to answer
Water intake	Integer, glasses/day	How many glasses of water do you drink each DAY?	-
Hot drink temperature	Categorical(single)	How do you like your hot drinks? (Such as coffee or tea)	Options: very hot, hot, warm, do not drink hot drinks, prefer not to answer
Tea intake	Integer, cups/day	How do you like your hot drinks? (Such as coffee or tea)	-
Alcohol intake frequency	Categorical(single)	About how often do you drink alcohol?	Options: daily or almost daily, three or four times a week, once or twice a week, one to three times a month, special occasions only, never, prefer not to answer

Supplementary Table 2. Significant SNP associated with exposures.

exposure||ID	samplesize	SNP	Effect_allel	Other_allel	eaf	beta	pval	se	MAF	R^2	FSTAT
Beef intake || id:ukb-b-2862	461053	rs11165829	G	C	0.35997	-0.0102	9.80E-09	0.001779	0.35997	7.13E-05	32.88713

Beef intake || id:ukb-b-2862	461053	rs1105388	T	C	0.300147	-0.011375	1.30E-09	0.001876	0.300147	7.98E-05	36.78215
Beef intake || id:ukb-b-2862	461053	rs10789340	G	A	0.626712	-0.01378	6.80E-15	0.001769	0.373288	1.32E-04	60.65201
Beef intake || id:ukb-b-2862	461053	rs1470610	C	G	0.196185	-0.012216	1.50E-08	0.002158	0.196185	6.95E-05	32.0502
Beef intake || id:ukb-b-2862	461053	rs62169335	T	C	0.543241	-0.009686	2.40E-08	0.001735	0.456759	6.76E-05	31.16004
Beef intake || id:ukb-b-2862	461053	rs4676964	T	C	0.510605	0.013353	9.60E-15	0.001724	0.489395	1.30E-04	59.96659
Beef intake || id:ukb-b-2862	461053	rs62396185	C	G	0.260057	-0.014845	2.80E-14	0.001951	0.260057	1.26E-04	57.89186
Beef intake || id:ukb-b-2862	461053	rs7791463	A	G	0.534781	0.009548	2.40E-08	0.001711	0.465219	6.76E-05	31.15678
Beef intake || id:ukb-b-2862	461053	rs79809011	A	G	0.029465	-0.028059	3.40E-08	0.005085	0.029465	6.60E-05	30.45093
Beef intake || id:ukb-b-2862	461053	rs9407624	A	T	0.488187	-0.013763	1.10E-15	0.001716	0.488187	1.40E-04	64.33099
Beef intake || id:ukb-b-2862	461053	rs10959890	C	T	0.212297	-0.012666	1.50E-09	0.002096	0.212297	7.92E-05	36.51197
Beef intake || id:ukb-b-2862	461053	rs12247907	C	G	0.485752	0.009841	8.90E-09	0.001711	0.485752	7.17E-05	33.06866
Beef intake || id:ukb-b-2862	461053	rs1421085	C	T	0.403433	-0.012123	3.50E-12	0.001743	0.403433	1.05E-04	48.39132
Beef intake || id:ukb-b-2862	461053	rs784251	T	C	0.477649	-0.010339	1.70E-09	0.001717	0.477649	7.87E-05	36.2775
Beef intake || id:ukb-b-2862	461053	rs429358	C	T	0.154226	-0.014851	3.60E-10	0.002368	0.154226	8.53E-05	39.31852
Beef intake || id:ukb-b-2862	461053	rs11878917	A	G	0.109688	0.015019	4.60E-08	0.002748	0.109688	6.48E-05	29.86699
Beef intake || id:ukb-b-2862	461053	rs132901	T	C	0.787662	0.013925	2.90E-11	0.002093	0.212338	9.60E-05	44.27092
Lamb/mutton intake || id:ukb-b-14179	460006	rs7550173	T	A	0.610281	-0.009124	1.30E-09	0.001502	0.389719	8.02E-05	36.88442
Lamb/mutton intake || id:ukb-b-14179	460006	rs660880	A	G	0.51281	-0.009032	6.80E-10	0.001464	0.48719	8.27E-05	38.0641
Lamb/mutton intake || id:ukb-b-14179	460006	rs56394517	G	A	0.09584	-0.013775	3.20E-08	0.002491	0.09584	6.65E-05	30.57341
Lamb/mutton intake || id:ukb-b-14179	460006	rs139237013	A	G	0.057668	0.018899	1.80E-09	0.00314	0.057668	7.87E-05	36.22367
Lamb/mutton intake || id:ukb-b-14179	460006	rs2222760	A	G	0.28089	-0.009093	2.80E-08	0.001637	0.28089	6.70E-05	30.84368
Lamb/mutton intake || id:ukb-b-14179	460006	rs62106258	C	T	0.048562	0.02163	2.00E-10	0.003402	0.048562	8.79E-05	40.43673
Lamb/mutton intake || id:ukb-b-14179	460006	rs2678900	G	T	0.427924	0.010084	9.90E-12	0.001481	0.427924	1.01E-04	46.35288
Lamb/mutton intake || id:ukb-b-14179	460006	rs2140714	G	C	0.557738	-0.008525	7.70E-09	0.001476	0.442262	7.25E-05	33.34094
Lamb/mutton intake || id:ukb-b-14179	460006	rs12634740	G	T	0.252078	-0.010087	2.80E-09	0.001697	0.252078	7.68E-05	35.32371
Lamb/mutton intake || id:ukb-b-14179	460006	rs6829572	A	G	0.456717	0.008401	1.20E-08	0.001474	0.456717	7.06E-05	32.48103
Lamb/mutton intake || id:ukb-b-14179	460006	rs16891982	G	C	0.972143	-0.024268	2.70E-08	0.004364	0.027857	6.72E-05	30.91908
Lamb/mutton intake || id:ukb-b-14179	460006	rs11743441	T	G	0.574343	-0.008842	2.70E-09	0.001486	0.425657	7.70E-05	35.408
Lamb/mutton intake || id:ukb-b-14179	460006	rs7447465	C	T	0.619449	0.009582	2.00E-10	0.001506	0.380551	8.80E-05	40.47382
Lamb/mutton intake || id:ukb-b-14179	460006	rs62398404	T	C	0.127211	0.012915	4.00E-09	0.002194	0.127211	7.53E-05	34.64158
Lamb/mutton intake || id:ukb-b-14179	460006	rs994270	G	C	0.234851	0.00982	1.40E-08	0.001729	0.234851	7.01E-05	32.2416
Lamb/mutton intake || id:ukb-b-14179	460006	rs35797675	G	T	0.215928	-0.01085	1.40E-09	0.001793	0.215928	7.96E-05	36.60052

Lamb/mutton intake || id:ukb-b-14179	460006	rs4272399	A	C	0.321491	-0.009242	4.50E-09	0.001576	0.321491	7.48E-05	34.40556
Lamb/mutton intake || id:ukb-b-14179	460006	rs276453	C	A	0.488356	-0.014237	2.90E-22	0.001467	0.488356	2.05E-04	94.15398
Lamb/mutton intake || id:ukb-b-14179	460006	rs1556147	T	A	0.671626	0.009101	5.30E-09	0.001559	0.328374	7.41E-05	34.07761
Lamb/mutton intake || id:ukb-b-14179	460006	rs673696	T	C	0.080995	0.015821	3.70E-09	0.002682	0.080995	7.56E-05	34.79561
Lamb/mutton intake || id:ukb-b-14179	460006	rs4489752	T	G	0.836007	0.013811	2.80E-12	0.001976	0.163993	1.06E-04	48.85804
Lamb/mutton intake || id:ukb-b-14179	460006	rs6581296	G	C	0.794714	0.01002	4.00E-08	0.001824	0.205286	6.56E-05	30.16602
Lamb/mutton intake || id:ukb-b-14179	460006	rs3105056	C	T	0.732738	-0.011624	1.80E-12	0.001649	0.267262	1.08E-04	49.71848
Lamb/mutton intake || id:ukb-b-14179	460006	rs1958801	G	A	0.287998	-0.008944	3.20E-08	0.001617	0.287998	6.65E-05	30.57523
Lamb/mutton intake || id:ukb-b-14179	460006	rs55813438	A	G	0.763165	-0.011418	4.70E-11	0.001735	0.236835	9.41E-05	43.29988
Lamb/mutton intake || id:ukb-b-14179	460006	rs2726033	G	A	0.422384	-0.00949	1.50E-10	0.00148	0.422384	8.93E-05	41.09048
Lamb/mutton intake || id:ukb-b-14179	460006	rs3964074	C	T	0.547043	-0.008141	3.20E-08	0.001472	0.452957	6.65E-05	30.5805
Lamb/mutton intake || id:ukb-b-14179	460006	rs2926119	A	C	0.569417	0.008111	4.40E-08	0.001482	0.430583	6.51E-05	29.96374
Lamb/mutton intake || id:ukb-b-14179	460006	rs429358	C	T	0.154171	-0.018197	2.70E-19	0.002027	0.154171	1.75E-04	80.6112
Lamb/mutton intake || id:ukb-b-14179	460006	rs17270057	C	T	0.113347	0.012653	4.30E-08	0.002309	0.113347	6.53E-05	30.03095
Lamb/mutton intake || id:ukb-b-14179	460006	rs136548	T	C	0.376668	0.009532	2.90E-10	0.001512	0.376668	8.64E-05	39.73241
Lamb/mutton intake || id:ukb-b-14179	460006	rs11090045	A	G	0.307145	-0.010676	3.00E-11	0.001607	0.307145	9.60E-05	44.15949
Pork intake || id:ukb-b-5640	460162	rs11211124	C	T	0.230603	-0.009953	1.40E-08	0.001754	0.230603	7.00E-05	32.19465
Pork intake || id:ukb-b-5640	460162	rs9973426	G	A	0.176598	0.011085	1.00E-08	0.001937	0.176598	7.12E-05	32.75559
Pork intake || id:ukb-b-5640	460162	rs7641973	A	G	0.353362	0.008446	4.20E-08	0.001541	0.353362	6.53E-05	30.0419
Pork intake || id:ukb-b-5640	460162	rs254152	G	C	0.23498	-0.010427	2.20E-09	0.001742	0.23498	7.78E-05	35.81809
Pork intake || id:ukb-b-5640	460162	rs9379832	G	A	0.255452	-0.011472	1.80E-11	0.001706	0.255452	9.82E-05	45.19995
Pork intake || id:ukb-b-5640	460162	rs10972033	T	G	0.45643	0.008976	1.30E-09	0.001479	0.45643	8.01E-05	36.85307
Pork intake || id:ukb-b-5640	460162	rs1355171	A	C	0.488844	-0.010983	1.00E-13	0.001477	0.488844	1.20E-04	55.29615
Pork intake || id:ukb-b-5640	460162	rs34161520	G	C	0.160381	0.011592	9.60E-09	0.002021	0.160381	7.15E-05	32.91142
Pork intake || id:ukb-b-5640	460162	rs2387807	T	C	0.077893	-0.015085	4.10E-08	0.002748	0.077893	6.55E-05	30.12419
Pork intake || id:ukb-b-5640	460162	rs4146837	T	C	0.455561	0.008792	4.00E-09	0.001494	0.455561	7.52E-05	34.61509
Pork intake || id:ukb-b-5640	460162	rs3964074	C	T	0.546932	-0.008951	1.60E-09	0.001483	0.453068	7.91E-05	36.40617
Pork intake || id:ukb-b-5640	460162	rs36124222	C	T	0.433239	0.008409	2.10E-08	0.001501	0.433239	6.82E-05	31.39496
Pork intake || id:ukb-b-5640	460162	rs12721051	G	C	0.188393	-0.012364	5.60E-11	0.001887	0.188393	9.33E-05	42.94452
Pork intake || id:ukb-b-5640	460162	rs838133	G	A	0.549354	0.010894	9.00E-13	0.001525	0.450646	1.11E-04	51.04066
Poultry intake || id:ukb-b-8006	461900	rs9997448	T	C	0.369236	-0.010465	2.70E-08	0.001883	0.369236	6.69E-05	30.88859
Poultry intake || id:ukb-b-8006	461900	rs7829800	G	A	0.670759	0.011458	3.70E-09	0.001943	0.329241	7.53E-05	34.78133

Poultry intake || id:ukb-b-8006	461900	rs7046351	A	T	0.509739	0.011075	1.10E-09	0.001817	0.490261	8.04E-05	37.13104
Poultry intake || id:ukb-b-8006	461900	rs1051730	A	G	0.331381	-0.010876	1.70E-08	0.001929	0.331381	6.88E-05	31.7762
Poultry intake || id:ukb-b-8006	461900	rs9923768	A	G	0.59853	0.010512	1.60E-08	0.001859	0.40147	6.92E-05	31.97713
Poultry intake || id:ukb-b-8006	461900	rs2565017	A	G	0.372697	0.010952	5.90E-09	0.001882	0.372697	7.33E-05	33.85753
Poultry intake || id:ukb-b-8006	461900	rs2965200	A	G	0.640011	-0.01042	4.20E-08	0.001901	0.359989	6.50E-05	30.04655
Poultry intake || id:ukb-b-8006	461900	rs2426440	G	A	0.732602	0.011218	4.70E-08	0.002053	0.267398	6.46E-05	29.84905
Processed meat intake || id:ukb-b-6324	461981	rs7531118	C	T	0.531133	-0.014072	2.80E-11	0.002113	0.468867	9.60E-05	44.3314
Processed meat intake || id:ukb-b-6324	461981	rs77165542	T	C	0.035496	0.033879	3.30E-09	0.005725	0.035496	7.58E-05	35.0183
Processed meat intake || id:ukb-b-6324	461981	rs11887120	T	C	0.397683	0.011962	3.10E-08	0.00216	0.397683	6.64E-05	30.65721
Processed meat intake || id:ukb-b-6324	461981	rs11894162	T	C	0.547464	0.012049	1.10E-08	0.002107	0.452536	7.08E-05	32.70183
Processed meat intake || id:ukb-b-6324	461981	rs4077924	C	T	0.701892	0.012499	4.50E-08	0.002285	0.298108	6.48E-05	29.92796
Processed meat intake || id:ukb-b-6324	461981	rs3762621	T	C	0.183451	-0.01499	3.60E-08	0.00272	0.183451	6.57E-05	30.3771
Processed meat intake || id:ukb-b-6324	461981	rs9809856	G	A	0.475881	0.013289	2.50E-10	0.0021	0.475881	8.67E-05	40.0554
Processed meat intake || id:ukb-b-6324	461981	rs2873054	C	A	0.353276	0.013996	1.60E-10	0.00219	0.353276	8.84E-05	40.84707
Processed meat intake || id:ukb-b-6324	461981	rs6786550	C	T	0.635043	0.012187	2.10E-08	0.002174	0.364957	6.80E-05	31.42301
Processed meat intake || id:ukb-b-6324	461981	rs6765179	A	G	0.309968	-0.01276	1.80E-08	0.002266	0.309968	6.86E-05	31.7086
Processed meat intake || id:ukb-b-6324	461981	rs10454812	C	A	0.103032	-0.01996	6.70E-09	0.003442	0.103032	7.28E-05	33.62602
Processed meat intake || id:ukb-b-6324	461981	rs2029401	G	A	0.586212	0.014631	6.30E-12	0.002129	0.413788	1.02E-04	47.2199
Processed meat intake || id:ukb-b-6324	461981	rs1422192	A	G	0.158076	0.016968	3.40E-09	0.002871	0.158076	7.56E-05	34.92315
Processed meat intake || id:ukb-b-6324	461981	rs6961970	A	C	0.244698	-0.014014	9.50E-09	0.002441	0.244698	7.13E-05	32.95092
Processed meat intake || id:ukb-b-6324	461981	rs4240672	A	G	0.494014	0.01712	3.00E-16	0.002094	0.494014	1.45E-04	66.83556
Processed meat intake || id:ukb-b-6324	461981	rs6484504	C	T	0.72462	0.015473	4.40E-11	0.002348	0.27538	9.40E-05	43.42869
Processed meat intake || id:ukb-b-6324	461981	rs11032380	T	A	0.333352	-0.013323	2.10E-09	0.002223	0.333352	7.77E-05	35.91999
Processed meat intake || id:ukb-b-6324	461981	rs4778053	G	C	0.843808	0.016467	1.30E-08	0.002899	0.156192	6.98E-05	32.26998
Processed meat intake || id:ukb-b-6324	461981	rs34241936	G	A	0.037369	0.032837	1.10E-08	0.005753	0.037369	7.05E-05	32.57411
Processed meat intake || id:ukb-b-6324	461981	rs8096167	C	T	0.1926	-0.014594	4.70E-08	0.002671	0.1926	6.46E-05	29.85611
Processed meat intake || id:ukb-b-6324	461981	rs838133	G	A	0.549369	0.019014	1.60E-18	0.002166	0.450631	1.67E-04	77.08945
Processed meat intake || id:ukb-b-6324	461981	rs6010651	C	A	0.379439	-0.012406	1.10E-08	0.002168	0.379439	7.09E-05	32.74554
Processed meat intake || id:ukb-b-6324	461981	rs203319	T	C	0.204594	-0.016423	2.80E-10	0.002602	0.204594	8.62E-05	39.84146
Non-oily fish intake || id:ukb-b-17627	460880	rs16822430	C	T	0.23321	0.011634	1.40E-09	0.00192	0.23321	7.97E-05	36.71992
Non-oily fish intake || id:ukb-b-17627	460880	rs1260326	C	T	0.604249	-0.009554	7.90E-09	0.001655	0.395751	7.23E-05	33.3099
Non-oily fish intake || id:ukb-b-17627	460880	rs11680516	C	T	0.202388	0.012284	1.40E-09	0.002028	0.202388	7.96E-05	36.68021

Non-oily fish intake || id:ukb-b-17627	460880	rs3799077	G	T	0.309993	-0.010735	1.00E-09	0.001758	0.309993	8.09E-05	37.3001
Non-oily fish intake || id:ukb-b-17627	460880	rs4318925	T	C	0.177245	-0.015038	1.30E-12	0.002121	0.177245	1.09E-04	50.28083
Non-oily fish intake || id:ukb-b-17627	460880	rs6957745	C	T	0.202978	-0.012179	1.80E-09	0.002023	0.202978	7.86E-05	36.23295
Non-oily fish intake || id:ukb-b-17627	460880	rs17317920	G	A	0.479238	0.00906	2.80E-08	0.001631	0.479238	6.69E-05	30.8509
Non-oily fish intake || id:ukb-b-17627	460880	rs35287743	T	G	0.11583	-0.01774	3.60E-12	0.002552	0.11583	1.05E-04	48.30579
Non-oily fish intake || id:ukb-b-17627	460880	rs7148387	G	A	0.590715	-0.009306	1.70E-08	0.00165	0.409285	6.90E-05	31.80419
Non-oily fish intake || id:ukb-b-17627	460880	rs56094641	G	A	0.404619	0.012588	2.50E-14	0.001651	0.404619	1.26E-04	58.12204
Non-oily fish intake || id:ukb-b-17627	460880	rs838133	G	A	0.549409	0.016184	4.70E-22	0.001676	0.450591	2.02E-04	93.21639
Oily fish intake || id:ukb-b-2209	460443	rs973526	T	C	0.513307	-0.011507	2.50E-09	0.00193	0.486693	7.72E-05	35.56139
Oily fish intake || id:ukb-b-2209	460443	rs45501495	T	C	0.236013	0.01568	3.70E-12	0.002257	0.236013	1.05E-04	48.26071
Oily fish intake || id:ukb-b-2209	460443	rs55930451	T	C	0.108001	-0.017054	2.90E-08	0.003076	0.108001	6.68E-05	30.7444
Oily fish intake || id:ukb-b-2209	460443	rs55985303	A	G	0.241079	0.012974	6.60E-09	0.002236	0.241079	7.31E-05	33.65655
Oily fish intake || id:ukb-b-2209	460443	rs17050031	T	C	0.4801	-0.012049	3.50E-10	0.001921	0.4801	8.55E-05	39.3546
Oily fish intake || id:ukb-b-2209	460443	rs275160	C	T	0.700597	0.012119	8.00E-09	0.002101	0.299403	7.23E-05	33.27222
Oily fish intake || id:ukb-b-2209	460443	rs13070166	A	T	0.228586	0.014211	4.40E-10	0.002278	0.228586	8.45E-05	38.91056
Oily fish intake || id:ukb-b-2209	460443	rs114497213	T	G	0.05481	0.027323	1.10E-10	0.004236	0.05481	9.03E-05	41.60225
Oily fish intake || id:ukb-b-2209	460443	rs10513136	A	G	0.065424	-0.023323	1.60E-09	0.003863	0.065424	7.92E-05	36.45098
Oily fish intake || id:ukb-b-2209	460443	rs1876245	C	T	0.431482	0.015116	5.00E-15	0.001931	0.431482	1.33E-04	61.26605
Oily fish intake || id:ukb-b-2209	460443	rs10510554	C	T	0.569277	0.011045	1.20E-08	0.001936	0.430723	7.07E-05	32.53313
Oily fish intake || id:ukb-b-2209	460443	rs905575	G	C	0.82397	0.013882	3.60E-08	0.002519	0.17603	6.59E-05	30.36773
Oily fish intake || id:ukb-b-2209	460443	rs9841174	C	T	0.373913	0.014778	8.50E-14	0.00198	0.373913	1.21E-04	55.69232
Oily fish intake || id:ukb-b-2209	460443	rs1201289	G	T	0.394525	-0.010727	4.40E-08	0.00196	0.394525	6.51E-05	29.96565
Oily fish intake || id:ukb-b-2209	460443	rs7683782	G	C	0.833405	0.014478	1.90E-08	0.002574	0.166595	6.87E-05	31.63527
Oily fish intake || id:ukb-b-2209	460443	rs10076975	C	T	0.381413	0.011239	1.10E-08	0.001967	0.381413	7.09E-05	32.64279
Oily fish intake || id:ukb-b-2209	460443	rs10061973	T	G	0.5139	-0.010852	1.50E-08	0.001916	0.4861	6.97E-05	32.08069
Oily fish intake || id:ukb-b-2209	460443	rs16891727	A	C	0.129814	-0.023717	6.80E-17	0.00284	0.129814	1.51E-04	69.74013
Oily fish intake || id:ukb-b-2209	460443	rs34555420	T	G	0.097821	-0.023768	1.50E-13	0.003219	0.097821	1.18E-04	54.51365
Oily fish intake || id:ukb-b-2209	460443	rs12663865	A	G	0.758185	0.012772	1.10E-08	0.002232	0.241815	7.11E-05	32.73225
Oily fish intake || id:ukb-b-2209	460443	rs4869859	C	T	0.449939	0.01401	3.10E-13	0.001922	0.449939	1.15E-04	53.12801
Oily fish intake || id:ukb-b-2209	460443	rs11767283	G	A	0.221709	0.017674	2.50E-14	0.002319	0.221709	1.26E-04	58.06049
Oily fish intake || id:ukb-b-2209	460443	rs6465487	G	A	0.399814	-0.012354	2.70E-10	0.001956	0.399814	8.66E-05	39.87642
Oily fish intake || id:ukb-b-2209	460443	rs11986122	G	C	0.422568	0.014817	2.90E-14	0.001949	0.422568	1.25E-04	57.77662

Oily fish intake || id:ukb-b-2209	460443	rs790564	C	A	0.722952	0.014691	7.90E-12	0.002148	0.277048	1.02E-04	46.78925
Oily fish intake || id:ukb-b-2209	460443	rs552234	A	G	0.495449	-0.011647	1.10E-09	0.001913	0.495449	8.05E-05	37.08891
Oily fish intake || id:ukb-b-2209	460443	rs9886779	A	T	0.439213	-0.010721	2.70E-08	0.001929	0.439213	6.71E-05	30.89409
Oily fish intake || id:ukb-b-2209	460443	rs10828250	G	C	0.309332	-0.020146	2.60E-22	0.002073	0.309332	2.05E-04	94.40913
Oily fish intake || id:ukb-b-2209	460443	rs703987	C	G	0.615005	0.011122	1.70E-08	0.001974	0.384995	6.90E-05	31.75566
Oily fish intake || id:ukb-b-2209	460443	rs61882686	A	C	0.085234	0.019756	8.00E-09	0.003425	0.085234	7.22E-05	33.26359
Oily fish intake || id:ukb-b-2209	460443	rs4278546	G	A	0.441085	0.012556	9.30E-11	0.001938	0.441085	9.11E-05	41.9693
Oily fish intake || id:ukb-b-2209	460443	rs2374424	G	A	0.601525	-0.011444	4.90E-09	0.001956	0.398475	7.43E-05	34.22578
Oily fish intake || id:ukb-b-2209	460443	rs510161	G	C	0.31037	-0.0113	4.50E-08	0.002066	0.31037	6.50E-05	29.90997
Oily fish intake || id:ukb-b-2209	460443	rs631490	C	G	0.709106	-0.01514	6.00E-13	0.002103	0.290894	1.13E-04	51.85062
Oily fish intake || id:ukb-b-2209	460443	rs303817	G	A	0.751146	0.01358	8.00E-10	0.00221	0.248854	8.20E-05	37.76976
Oily fish intake || id:ukb-b-2209	460443	rs35287743	T	G	0.115869	-0.02822	7.00E-21	0.003011	0.115869	1.91E-04	87.8572
Oily fish intake || id:ukb-b-2209	460443	rs9597870	G	T	0.245844	-0.012745	1.10E-08	0.00223	0.245844	7.09E-05	32.64992
Oily fish intake || id:ukb-b-2209	460443	rs3124402	G	A	0.733261	-0.022001	1.90E-24	0.002157	0.266739	2.26E-04	104.0868
Oily fish intake || id:ukb-b-2209	460443	rs12855717	T	C	0.526774	-0.01223	2.00E-10	0.001923	0.473226	8.79E-05	40.45677
Oily fish intake || id:ukb-b-2209	460443	rs1361016	G	T	0.844853	0.014957	1.70E-08	0.002653	0.155147	6.90E-05	31.78422
Oily fish intake || id:ukb-b-2209	460443	rs9301837	A	C	0.143273	-0.015743	8.10E-09	0.00273	0.143273	7.22E-05	33.2425
Oily fish intake || id:ukb-b-2209	460443	rs4982738	A	G	0.582899	0.010861	3.50E-08	0.001969	0.417101	6.60E-05	30.41386
Oily fish intake || id:ukb-b-2209	460443	rs12896749	C	G	0.384721	-0.010964	2.50E-08	0.001966	0.384721	6.75E-05	31.09898
Oily fish intake || id:ukb-b-2209	460443	rs1951286	G	T	0.644911	-0.014583	3.00E-13	0.001999	0.355089	1.16E-04	53.19559
Oily fish intake || id:ukb-b-2209	460443	rs28533540	A	G	0.534203	0.014642	2.80E-14	0.001925	0.465797	1.26E-04	57.86856
Oily fish intake || id:ukb-b-2209	460443	rs1421085	C	T	0.40341	0.018481	2.50E-21	0.001949	0.40341	1.95E-04	89.94241
Oily fish intake || id:ukb-b-2209	460443	rs11859365	C	A	0.253765	0.022567	9.40E-25	0.002197	0.253765	2.29E-04	105.5273
Oily fish intake || id:ukb-b-2209	460443	rs9889161	T	G	0.357929	-0.01332	2.80E-11	0.002002	0.357929	9.62E-05	44.28961
Oily fish intake || id:ukb-b-2209	460443	rs28623270	T	A	0.14872	-0.017833	7.30E-11	0.002737	0.14872	9.22E-05	42.4367
Oily fish intake || id:ukb-b-2209	460443	rs2952140	T	C	0.482607	-0.010677	2.50E-08	0.001915	0.482607	6.75E-05	31.08662
Oily fish intake || id:ukb-b-2209	460443	rs4510068	T	G	0.402798	-0.013014	4.00E-11	0.001971	0.402798	9.47E-05	43.6006
Oily fish intake || id:ukb-b-2209	460443	rs7243428	G	A	0.224648	-0.012978	1.50E-08	0.002292	0.224648	6.96E-05	32.06016
Oily fish intake || id:ukb-b-2209	460443	rs9958909	G	T	0.139718	0.015762	1.40E-08	0.002778	0.139718	6.99E-05	32.18931
Oily fish intake || id:ukb-b-2209	460443	rs59355765	T	C	0.160079	-0.016252	4.70E-10	0.00261	0.160079	8.42E-05	38.78418
Oily fish intake || id:ukb-b-2209	460443	rs4002471	T	C	0.547362	-0.019244	1.50E-23	0.001924	0.452638	2.17E-04	99.99853
Oily fish intake || id:ukb-b-2209	460443	rs7254235	G	A	0.577254	-0.01063	4.30E-08	0.00194	0.422746	6.52E-05	30.02204

Oily fish intake || id:ukb-b-2209	460443	rs75887709	G	A	0.13588	-0.015911	1.60E-08	0.002815	0.13588	6.94E-05	31.95039
Oily fish intake || id:ukb-b-2209	460443	rs12983532	T	C	0.251137	-0.01339	2.00E-09	0.002233	0.251137	7.81E-05	35.94356
Oily fish intake || id:ukb-b-2209	460443	rs6033437	A	C	0.257329	0.012468	1.70E-08	0.00221	0.257329	6.91E-05	31.836
Oily fish intake || id:ukb-b-2209	460443	rs6059844	G	A	0.495121	0.011	9.20E-09	0.001915	0.495121	7.17E-05	33.01232
Oily fish intake || id:ukb-b-2209	460443	rs6089753	T	C	0.530967	-0.011542	1.80E-09	0.001918	0.469033	7.86E-05	36.19778
Oily fish intake || id:ukb-b-2209	460443	rs2827161	G	T	0.422842	0.010711	3.20E-08	0.001937	0.422842	6.64E-05	30.57357
Oily fish intake || id:ukb-b-2209	460443	rs9606833	C	T	0.243593	0.016986	2.70E-14	0.002231	0.243593	1.26E-04	57.97108
Cooked vegetable intake || id:ukb-b-8089	448651	rs2252508	G	A	0.480256	0.009102	5.70E-09	0.001562	0.480256	7.57E-05	33.94925
Cooked vegetable intake || id:ukb-b-8089	448651	rs2102738	C	A	0.172403	-0.012158	5.30E-09	0.002083	0.172403	7.59E-05	34.066
Cooked vegetable intake || id:ukb-b-8089	448651	rs4851029	G	T	0.526969	0.010174	7.80E-11	0.001564	0.473031	9.43E-05	42.30722
Cooked vegetable intake || id:ukb-b-8089	448651	rs12629972	C	T	0.588311	0.0118	1.20E-13	0.001591	0.411689	1.23E-04	55.01734
Cooked vegetable intake || id:ukb-b-8089	448651	rs28450747	A	G	0.23257	-0.01016	4.30E-08	0.001855	0.23257	6.69E-05	30.01044
Cooked vegetable intake || id:ukb-b-8089	448651	rs1816263	C	T	0.280188	0.009581	3.70E-08	0.001741	0.280188	6.75E-05	30.29889
Cooked vegetable intake || id:ukb-b-8089	448651	rs2844672	A	G	0.624125	-0.009642	2.10E-09	0.001609	0.375875	8.00E-05	35.90125
Cooked vegetable intake || id:ukb-b-8089	448651	rs12550717	A	G	0.372413	0.009187	1.40E-08	0.001619	0.372413	7.18E-05	32.20349
Cooked vegetable intake || id:ukb-b-8089	448651	rs10156602	G	A	0.361369	0.011006	1.80E-11	0.001637	0.361369	1.01E-04	45.21186
Cooked vegetable intake || id:ukb-b-8089	448651	rs11138705	C	G	0.757204	0.010372	1.40E-08	0.00183	0.242796	7.16E-05	32.11885
Cooked vegetable intake || id:ukb-b-8089	448651	rs2052063	T	C	0.515902	-0.009458	1.60E-09	0.001568	0.484098	8.11E-05	36.38469
Cooked vegetable intake || id:ukb-b-8089	448651	rs349062	C	G	0.39275	-0.008904	2.50E-08	0.001598	0.39275	6.92E-05	31.0599
Cooked vegetable intake || id:ukb-b-8089	448651	rs28711392	C	T	0.367132	-0.010746	4.60E-11	0.001632	0.367132	9.66E-05	43.34481
Cooked vegetable intake || id:ukb-b-8089	448651	rs10161952	C	A	0.31274	-0.00958	1.30E-08	0.001686	0.31274	7.20E-05	32.28884
Cooked vegetable intake || id:ukb-b-8089	448651	rs1421085	C	T	0.40346	0.010332	8.30E-11	0.001591	0.40346	9.40E-05	42.19335
Cooked vegetable intake || id:ukb-b-8089	448651	rs838133	G	A	0.549575	0.011688	4.50E-13	0.001615	0.450425	1.17E-04	52.39615
Cooked vegetable intake || id:ukb-b-8089	448651	rs34155012	T	C	0.227386	0.010558	3.90E-08	0.001922	0.227386	6.73E-05	30.17528
Salad / raw vegetable intake || id:ukb-b-1996	435435	rs9427220	T	A	0.554661	-0.008008	2.80E-08	0.001442	0.445339	7.08E-05	30.83503
Salad / raw vegetable intake || id:ukb-b-1996	435435	rs4083969	G	C	0.057217	0.017133	3.80E-08	0.003115	0.057217	6.95E-05	30.24833
Salad / raw vegetable intake || id:ukb-b-1996	435435	rs7619139	A	T	0.589193	0.012477	8.00E-18	0.001451	0.410807	1.70E-04	73.94904
Salad / raw vegetable intake || id:ukb-b-1996	435435	rs13102393	G	C	0.499077	0.007979	2.40E-08	0.00143	0.499077	7.15E-05	31.12309
Salad / raw vegetable intake || id:ukb-b-1996	435435	rs17460017	T	A	0.190124	0.011174	7.20E-10	0.001813	0.190124	8.72E-05	37.97246
Salad / raw vegetable intake || id:ukb-b-1996	435435	rs2194027	A	T	0.484664	-0.008608	2.00E-09	0.001436	0.484664	8.26E-05	35.95012
Salad / raw vegetable intake || id:ukb-b-1996	435435	rs3129962	A	G	0.129168	-0.013303	3.70E-10	0.002123	0.129168	9.02E-05	39.28044
Salad / raw vegetable intake || id:ukb-b-1996	435435	rs12203592	T	C	0.219338	-0.010286	1.30E-09	0.001694	0.219338	8.47E-05	36.86589

Salad / raw vegetable intake || id:ukb-b-1996	435435	rs3095337	C	G	0.203909	-0.012622	9.00E-13	0.001766	0.203909	1.17E-04	51.05958
Salad / raw vegetable intake || id:ukb-b-1996	435435	rs75248709	T	C	0.045947	-0.019734	2.20E-08	0.003528	0.045947	7.19E-05	31.29181
Salad / raw vegetable intake || id:ukb-b-1996	435435	rs57221424	G	C	0.321733	0.008941	5.50E-09	0.001533	0.321733	7.81E-05	34.0043
Salad / raw vegetable intake || id:ukb-b-1996	435435	rs62461186	C	A	0.179827	-0.011344	1.00E-09	0.001857	0.179827	8.57E-05	37.30282
Salad / raw vegetable intake || id:ukb-b-1996	435435	rs790561	G	A	0.704134	0.012474	1.40E-15	0.001562	0.295866	1.46E-04	63.77537
Salad / raw vegetable intake || id:ukb-b-1996	435435	rs7821179	C	G	0.846577	-0.010818	4.40E-08	0.001976	0.153423	6.88E-05	29.96103
Salad / raw vegetable intake || id:ukb-b-1996	435435	rs10819082	A	G	0.66734	-0.009165	1.40E-09	0.001513	0.33266	8.42E-05	36.67079
Salad / raw vegetable intake || id:ukb-b-1996	435435	rs6482190	G	A	0.719408	0.011272	1.40E-12	0.00159	0.280592	1.15E-04	50.24137
Salad / raw vegetable intake || id:ukb-b-1996	435435	rs1890012	G	T	0.19473	-0.010426	8.10E-09	0.001808	0.19473	7.64E-05	33.25437
Salad / raw vegetable intake || id:ukb-b-1996	435435	rs12908495	A	C	0.242533	-0.00935	2.00E-08	0.001666	0.242533	7.24E-05	31.50736
Salad / raw vegetable intake || id:ukb-b-1996	435435	rs1052352	T	C	0.52355	0.008164	1.00E-08	0.001426	0.47645	7.53E-05	32.79677
Salad / raw vegetable intake || id:ukb-b-1996	435435	rs34186148	C	G	0.370054	-0.008051	4.80E-08	0.001475	0.370054	6.84E-05	29.80011
Salad / raw vegetable intake || id:ukb-b-1996	435435	rs4291983	A	C	0.517576	-0.0084	3.70E-09	0.001425	0.482424	7.98E-05	34.75579
Salad / raw vegetable intake || id:ukb-b-1996	435435	rs8130508	A	G	0.289686	0.00874	3.00E-08	0.001578	0.289686	7.05E-05	30.68055
Dried fruit intake || id:ukb-b-16576	421764	rs261809	G	A	0.540636	-0.009629	9.80E-09	0.001679	0.459364	7.80E-05	32.88597
Dried fruit intake || id:ukb-b-16576	421764	rs11586016	C	G	0.371004	0.009878	1.10E-08	0.00173	0.371004	7.73E-05	32.59034
Dried fruit intake || id:ukb-b-16576	421764	rs12137234	T	C	0.303772	0.010205	2.80E-08	0.001837	0.303772	7.31E-05	30.84652
Dried fruit intake || id:ukb-b-16576	421764	rs72720396	G	A	0.229157	0.011427	8.70E-09	0.001985	0.229157	7.85E-05	33.12257
Dried fruit intake || id:ukb-b-16576	421764	rs11811826	A	T	0.224231	0.013218	4.40E-11	0.002006	0.224231	1.03E-04	43.42512
Dried fruit intake || id:ukb-b-16576	421764	rs3101339	C	A	0.603285	0.01426	6.20E-17	0.001705	0.396715	1.66E-04	69.90745
Dried fruit intake || id:ukb-b-16576	421764	rs75641275	C	A	0.143372	-0.014161	2.90E-09	0.002385	0.143372	8.36E-05	35.25022
Dried fruit intake || id:ukb-b-16576	421764	rs7582086	T	G	0.468273	-0.009628	8.80E-09	0.001674	0.468273	7.84E-05	33.08847
Dried fruit intake || id:ukb-b-16576	421764	rs7599488	T	C	0.426408	-0.010415	6.70E-10	0.001687	0.426408	9.03E-05	38.10398
Dried fruit intake || id:ukb-b-16576	421764	rs4149513	A	G	0.493537	0.01173	2.20E-12	0.001671	0.493537	1.17E-04	49.25364
Dried fruit intake || id:ukb-b-16576	421764	rs17184707	T	C	0.212811	-0.011438	2.10E-08	0.00204	0.212811	7.45E-05	31.43392
Dried fruit intake || id:ukb-b-16576	421764	rs4269101	G	T	0.718948	-0.01381	1.10E-13	0.001859	0.281052	1.31E-04	55.17243
Dried fruit intake || id:ukb-b-16576	421764	rs11720884	G	A	0.250137	0.01118	7.60E-09	0.001936	0.250137	7.91E-05	33.36214
Dried fruit intake || id:ukb-b-16576	421764	rs57499472	C	T	0.404131	0.009912	8.10E-09	0.001719	0.404131	7.89E-05	33.26232
Dried fruit intake || id:ukb-b-16576	421764	rs10026792	A	G	0.290404	0.010847	3.90E-09	0.001842	0.290404	8.22E-05	34.66223
Dried fruit intake || id:ukb-b-16576	421764	rs1648404	T	C	0.476112	0.009416	1.80E-08	0.001674	0.476112	7.50E-05	31.65472
Dried fruit intake || id:ukb-b-16576	421764	rs746868	G	C	0.614662	-0.012906	5.20E-14	0.001715	0.385338	1.34E-04	56.65816
Dried fruit intake || id:ukb-b-16576	421764	rs9385269	T	C	0.524565	0.012067	7.20E-13	0.001682	0.475435	1.22E-04	51.48183

Dried fruit intake || id:ukb-b-16576	421764	rs2328887	C	T	0.899467	0.018948	8.80E-12	0.002776	0.100533	1.10E-04	46.58789
Dried fruit intake || id:ukb-b-16576	421764	rs2533273	A	C	0.48453	-0.009877	3.90E-09	0.001677	0.48453	8.22E-05	34.67954
Dried fruit intake || id:ukb-b-16576	421764	rs7808471	C	T	0.322136	-0.011536	1.10E-10	0.001786	0.322136	9.89E-05	41.7197
Dried fruit intake || id:ukb-b-16576	421764	rs11772627	C	G	0.18202	0.018334	3.00E-17	0.002171	0.18202	1.69E-04	71.31605
Dried fruit intake || id:ukb-b-16576	421764	rs7829800	G	A	0.671041	-0.010446	5.10E-09	0.001787	0.328959	8.10E-05	34.16883
Dried fruit intake || id:ukb-b-16576	421764	rs10740991	C	G	0.717606	0.016739	2.00E-19	0.001857	0.282394	1.93E-04	81.23258
Dried fruit intake || id:ukb-b-16576	421764	rs7916868	T	A	0.503499	0.009607	9.10E-09	0.001672	0.496501	7.83E-05	33.03007
Dried fruit intake || id:ukb-b-16576	421764	rs893856	A	G	0.148988	-0.013361	1.30E-08	0.002349	0.148988	7.67E-05	32.34631
Dried fruit intake || id:ukb-b-16576	421764	rs10896126	G	A	0.303582	-0.015009	1.60E-16	0.001819	0.303582	1.61E-04	68.06837
Dried fruit intake || id:ukb-b-16576	421764	rs11037497	C	G	0.446175	0.01044	5.70E-10	0.001684	0.446175	9.11E-05	38.41182
Dried fruit intake || id:ukb-b-16576	421764	rs1622515	G	A	0.484704	0.009917	2.90E-09	0.001671	0.484704	8.35E-05	35.23237
Dried fruit intake || id:ukb-b-16576	421764	rs3764002	T	C	0.261416	0.013122	5.10E-12	0.001901	0.261416	1.13E-04	47.6527
Dried fruit intake || id:ukb-b-16576	421764	rs4140799	A	G	0.531856	0.009457	1.80E-08	0.001678	0.468144	7.53E-05	31.74286
Dried fruit intake || id:ukb-b-16576	421764	rs34162196	T	C	0.101001	-0.022363	7.10E-16	0.002772	0.101001	1.54E-04	65.09747
Dried fruit intake || id:ukb-b-16576	421764	rs10129747	G	A	0.530254	0.009359	2.60E-08	0.001681	0.469746	7.35E-05	30.98767
Dried fruit intake || id:ukb-b-16576	421764	rs1797235	C	G	0.374623	-0.010021	8.90E-09	0.001742	0.374623	7.84E-05	33.07612
Dried fruit intake || id:ukb-b-16576	421764	rs11632215	C	A	0.120179	-0.014143	4.40E-08	0.002584	0.120179	7.10E-05	29.96271
Dried fruit intake || id:ukb-b-16576	421764	rs862227	G	A	0.458327	-0.009165	4.30E-08	0.001672	0.458327	7.12E-05	30.02998
Dried fruit intake || id:ukb-b-16576	421764	rs1582322	G	A	0.604805	0.009943	6.80E-09	0.001716	0.395195	7.96E-05	33.58808
Dried fruit intake || id:ukb-b-16576	421764	rs62084586	C	T	0.165729	0.013395	3.20E-09	0.002262	0.165729	8.32E-05	35.07293
Dried fruit intake || id:ukb-b-16576	421764	rs8081370	T	C	0.910232	-0.016667	1.40E-08	0.002938	0.089768	7.63E-05	32.18127
Dried fruit intake || id:ukb-b-16576	421764	rs4800488	A	C	0.489858	0.011984	7.70E-13	0.001672	0.489858	1.22E-04	51.36764
Dried fruit intake || id:ukb-b-16576	421764	rs17175518	A	C	0.232775	0.011496	5.90E-09	0.001975	0.232775	8.03E-05	33.87746
Dried fruit intake || id:ukb-b-16576	421764	rs11152349	A	G	0.302856	0.009912	4.90E-08	0.001818	0.302856	7.05E-05	29.73973
Dried fruit intake || id:ukb-b-16576	421764	rs429358	C	T	0.154208	0.019945	6.70E-18	0.002314	0.154208	1.76E-04	74.31221
Fresh fruit intake || id:ukb-b-3881	446462	rs2790688	T	C	0.154068	0.011447	1.50E-11	0.001696	0.154068	1.02E-04	45.54417
Fresh fruit intake || id:ukb-b-3881	446462	rs559734	C	G	0.711817	0.007768	1.10E-08	0.001361	0.288183	7.30E-05	32.58722
Fresh fruit intake || id:ukb-b-3881	446462	rs12044599	G	A	0.21008	0.009421	3.70E-10	0.001503	0.21008	8.80E-05	39.27135
Fresh fruit intake || id:ukb-b-3881	446462	rs7554485	C	T	0.611539	-0.00801	1.70E-10	0.001254	0.388461	9.14E-05	40.79305
Fresh fruit intake || id:ukb-b-3881	446462	rs1620977	G	A	0.730517	-0.013174	1.10E-21	0.001378	0.269483	2.05E-04	91.45216
Fresh fruit intake || id:ukb-b-3881	446462	rs2867113	A	G	0.130942	-0.013861	1.50E-12	0.00196	0.130942	1.12E-04	49.99645
Fresh fruit intake || id:ukb-b-3881	446462	rs17049185	T	G	0.267872	0.00804	7.30E-09	0.00139	0.267872	7.49E-05	33.4442

Fresh fruit intake || id:ukb-b-3881	446462	rs11896330	A	G	0.632839	-0.008446	3.40E-11	0.001274	0.367161	9.84E-05	43.91923
Fresh fruit intake || id:ukb-b-3881	446462	rs72974263	T	C	0.318282	0.007387	1.80E-08	0.001312	0.318282	7.10E-05	31.67888
Fresh fruit intake || id:ukb-b-3881	446462	rs817223	C	T	0.481314	-0.007269	2.80E-09	0.001223	0.481314	7.91E-05	35.30426
Fresh fruit intake || id:ukb-b-3881	446462	rs4953150	T	C	0.344088	-0.008439	6.60E-11	0.001293	0.344088	9.55E-05	42.6206
Fresh fruit intake || id:ukb-b-3881	446462	rs10192394	T	C	0.528785	-0.007661	4.50E-10	0.001229	0.471215	8.71E-05	38.87521
Fresh fruit intake || id:ukb-b-3881	446462	rs1375566	A	G	0.627412	-0.007834	6.10E-10	0.001266	0.372588	8.58E-05	38.28933
Fresh fruit intake || id:ukb-b-3881	446462	rs13072255	C	A	0.493966	0.00898	2.10E-13	0.001224	0.493966	1.21E-04	53.86652
Fresh fruit intake || id:ukb-b-3881	446462	rs1356292	T	C	0.807546	0.009175	3.50E-09	0.001553	0.192454	7.81E-05	34.89037
Fresh fruit intake || id:ukb-b-3881	446462	rs12641371	T	C	0.433148	0.007913	1.40E-10	0.001234	0.433148	9.22E-05	41.14737
Fresh fruit intake || id:ukb-b-3881	446462	rs10064431	C	T	0.522495	-0.007574	6.00E-10	0.001224	0.477505	8.58E-05	38.31114
Fresh fruit intake || id:ukb-b-3881	446462	rs149449	A	G	0.489167	0.007289	2.40E-09	0.001221	0.489167	7.99E-05	35.6555
Fresh fruit intake || id:ukb-b-3881	446462	rs2143081	A	G	0.539822	0.008321	1.30E-11	0.001229	0.460178	1.03E-04	45.84382
Fresh fruit intake || id:ukb-b-3881	446462	rs586346	C	T	0.635353	-0.006922	4.50E-08	0.001266	0.364647	6.70E-05	29.90011
Fresh fruit intake || id:ukb-b-3881	446462	rs994270	G	C	0.235126	0.013259	4.20E-20	0.001444	0.235126	1.89E-04	84.31768
Fresh fruit intake || id:ukb-b-3881	446462	rs329274	G	A	0.485636	0.006818	2.80E-08	0.001228	0.485636	6.90E-05	30.82205
Fresh fruit intake || id:ukb-b-3881	446462	rs10271924	T	C	0.492595	-0.007047	2.00E-08	0.001256	0.492595	7.05E-05	31.48133
Fresh fruit intake || id:ukb-b-3881	446462	rs10249294	A	G	0.372973	0.019564	4.10E-54	0.001263	0.372973	5.37E-04	239.9387
Fresh fruit intake || id:ukb-b-3881	446462	rs12536253	C	G	0.24901	-0.008159	8.30E-09	0.001416	0.24901	7.44E-05	33.20453
Fresh fruit intake || id:ukb-b-3881	446462	rs7818437	C	T	0.235903	-0.008048	3.00E-08	0.001453	0.235903	6.88E-05	30.70096
Fresh fruit intake || id:ukb-b-3881	446462	rs1866823	A	G	0.544469	0.007431	2.10E-09	0.001241	0.455531	8.04E-05	35.87604
Fresh fruit intake || id:ukb-b-3881	446462	rs6475724	T	C	0.727296	0.007722	1.90E-08	0.001374	0.272704	7.07E-05	31.57946
Fresh fruit intake || id:ukb-b-3881	446462	rs7869969	G	A	0.330836	0.007567	5.70E-09	0.001299	0.330836	7.60E-05	33.92687
Fresh fruit intake || id:ukb-b-3881	446462	rs4302893	A	G	0.334189	0.007389	1.30E-08	0.0013	0.334189	7.24E-05	32.32259
Fresh fruit intake || id:ukb-b-3881	446462	rs2093654	G	A	0.388133	0.007129	1.50E-08	0.001259	0.388133	7.18E-05	32.07757
Fresh fruit intake || id:ukb-b-3881	446462	rs11248509	T	A	0.371237	0.00733	7.40E-09	0.001268	0.371237	7.48E-05	33.41401
Fresh fruit intake || id:ukb-b-3881	446462	rs9919429	G	A	0.486008	-0.006723	3.80E-08	0.001223	0.486008	6.77E-05	30.22581
Fresh fruit intake || id:ukb-b-3881	446462	rs10828266	G	A	0.715634	0.012364	8.10E-20	0.001357	0.284366	1.86E-04	83.02254
Fresh fruit intake || id:ukb-b-3881	446462	rs12780952	A	G	0.286382	0.007475	3.40E-08	0.001354	0.286382	6.83E-05	30.49182
Fresh fruit intake || id:ukb-b-3881	446462	rs10840126	G	A	0.376121	-0.007718	1.90E-09	0.001286	0.376121	8.07E-05	36.02766
Fresh fruit intake || id:ukb-b-3881	446462	rs10838724	T	G	0.367992	0.009011	2.10E-12	0.001283	0.367992	1.11E-04	49.34406
Fresh fruit intake || id:ukb-b-3881	446462	rs11032362	A	G	0.090976	0.012393	5.30E-09	0.002123	0.090976	7.63E-05	34.06312
Fresh fruit intake || id:ukb-b-3881	446462	rs60452247	A	G	0.363079	0.00797	3.40E-10	0.001269	0.363079	8.83E-05	39.422

Fresh fruit intake || id:ukb-b-3881	446462	rs7982441	C	T	0.731883	-0.008413	9.80E-10	0.001376	0.268117	8.37E-05	37.3707
Fresh fruit intake || id:ukb-b-3881	446462	rs9517948	T	C	0.451352	0.006953	1.70E-08	0.001233	0.451352	7.12E-05	31.80729
Fresh fruit intake || id:ukb-b-3881	446462	rs12885598	A	G	0.596671	0.007515	1.70E-09	0.001247	0.403329	8.13E-05	36.30071
Fresh fruit intake || id:ukb-b-3881	446462	rs34162196	T	C	0.1008	-0.018142	4.00E-19	0.00203	0.1008	1.79E-04	79.88961
Fresh fruit intake || id:ukb-b-3881	446462	rs28479795	T	C	0.221473	0.011232	2.50E-14	0.001473	0.221473	1.30E-04	58.11288
Fresh fruit intake || id:ukb-b-3881	446462	rs62051554	A	G	0.108545	0.011612	4.60E-09	0.001981	0.108545	7.69E-05	34.34311
Fresh fruit intake || id:ukb-b-3881	446462	rs862227	G	A	0.457858	-0.010145	1.10E-16	0.001224	0.457858	1.54E-04	68.7022
Fresh fruit intake || id:ukb-b-3881	446462	rs1051547	C	T	0.561537	-0.007575	1.10E-09	0.001242	0.438463	8.34E-05	37.22368
Fresh fruit intake || id:ukb-b-3881	446462	rs139042899	C	A	0.013469	0.035995	3.20E-09	0.006081	0.013469	7.85E-05	35.03675
Fresh fruit intake || id:ukb-b-3881	446462	rs2048522	T	A	0.434968	0.009563	1.80E-14	0.001248	0.434968	1.32E-04	58.73725
Fresh fruit intake || id:ukb-b-3881	446462	rs73455661	G	A	0.279338	0.010294	4.10E-14	0.001362	0.279338	1.28E-04	57.10416
Fresh fruit intake || id:ukb-b-3881	446462	rs8095324	G	A	0.404041	-0.006946	2.70E-08	0.001249	0.404041	6.93E-05	30.92702
Fresh fruit intake || id:ukb-b-3881	446462	rs11085749	A	G	0.387076	-0.007734	7.10E-10	0.001255	0.387076	8.51E-05	37.98694
Fresh fruit intake || id:ukb-b-3881	446462	rs2302593	G	C	0.486625	0.008528	3.10E-12	0.001223	0.486625	1.09E-04	48.62059
Fresh fruit intake || id:ukb-b-3881	446462	rs739320	C	T	0.60575	-0.008997	1.90E-12	0.001277	0.39425	1.11E-04	49.63491
Fresh fruit intake || id:ukb-b-3881	446462	rs78537042	A	C	0.086801	-0.011926	4.80E-08	0.002184	0.086801	6.68E-05	29.80903
Bread intake || id:ukb-b-11348	452236	rs9662365	T	C	0.499481	0.012166	9.60E-10	0.001989	0.499481	8.27E-05	37.40585
Bread intake || id:ukb-b-11348	452236	rs13023099	A	C	0.571784	-0.011471	1.40E-08	0.002023	0.428216	7.11E-05	32.15508
Bread intake || id:ukb-b-11348	452236	rs6754311	C	T	0.264485	0.014135	3.10E-10	0.002247	0.264485	8.75E-05	39.58843
Bread intake || id:ukb-b-11348	452236	rs4665972	C	T	0.604541	-0.014236	3.20E-12	0.002043	0.395459	1.07E-04	48.53942
Bread intake || id:ukb-b-11348	452236	rs75287965	A	G	0.062933	-0.024748	1.50E-09	0.004097	0.062933	8.07E-05	36.4782
Bread intake || id:ukb-b-11348	452236	rs13016665	A	C	0.423235	0.014754	3.50E-13	0.002028	0.423235	1.17E-04	52.91528
Bread intake || id:ukb-b-11348	452236	rs1492988	G	C	0.599095	0.011545	1.40E-08	0.002035	0.400905	7.11E-05	32.1681
Bread intake || id:ukb-b-11348	452236	rs9832088	A	T	0.521617	0.014729	1.30E-13	0.001989	0.478383	1.21E-04	54.83532
Bread intake || id:ukb-b-11348	452236	rs9881332	G	C	0.581804	0.01137	1.90E-08	0.002022	0.418196	6.99E-05	31.62449
Bread intake || id:ukb-b-11348	452236	rs1994315	C	T	0.685162	-0.016824	4.30E-15	0.002144	0.314838	1.36E-04	61.5488
Bread intake || id:ukb-b-11348	452236	rs73802707	T	C	0.153694	-0.01593	8.00E-09	0.002762	0.153694	7.36E-05	33.27136
Bread intake || id:ukb-b-11348	452236	rs2068650	C	A	0.472074	-0.013938	3.10E-12	0.001999	0.472074	1.07E-04	48.60125
Bread intake || id:ukb-b-11348	452236	rs17083079	A	G	0.047423	0.030114	1.20E-10	0.004679	0.047423	9.16E-05	41.41451
Bread intake || id:ukb-b-11348	452236	rs2517678	T	C	0.368395	0.013233	2.20E-10	0.002086	0.368395	8.90E-05	40.24338
Bread intake || id:ukb-b-11348	452236	rs596878	C	A	0.449595	-0.011739	5.30E-09	0.002011	0.449595	7.54E-05	34.08553
Bread intake || id:ukb-b-11348	452236	rs79436018	C	T	0.116276	-0.017615	1.60E-08	0.003116	0.116276	7.06E-05	31.94687

Bread intake || id:ukb-b-11348	452236	rs7802468	T	C	0.371501	-0.023353	6.90E-30	0.002056	0.371501	2.85E-04	128.9744
Bread intake || id:ukb-b-11348	452236	rs10761661	T	C	0.45322	-0.011485	1.00E-08	0.002005	0.45322	7.26E-05	32.81762
Bread intake || id:ukb-b-11348	452236	rs55745436	T	C	0.237279	0.013425	1.00E-08	0.002343	0.237279	7.26E-05	32.83877
Bread intake || id:ukb-b-11348	452236	rs1940033	T	C	0.592727	-0.011079	4.70E-08	0.002028	0.407273	6.60E-05	29.83178
Bread intake || id:ukb-b-11348	452236	rs11183201	C	T	0.507856	-0.016725	5.30E-17	0.001996	0.492144	1.55E-04	70.22996
Bread intake || id:ukb-b-11348	452236	rs6580721	G	A	0.188665	0.017205	1.30E-11	0.002544	0.188665	1.01E-04	45.75377
Bread intake || id:ukb-b-11348	452236	rs11060853	G	A	0.411804	-0.012789	2.50E-10	0.002021	0.411804	8.86E-05	40.04926
Bread intake || id:ukb-b-11348	452236	rs9564268	C	T	0.615619	-0.012161	3.00E-09	0.002049	0.384381	7.79E-05	35.21182
Bread intake || id:ukb-b-11348	452236	rs9529024	T	A	0.370396	-0.012917	4.20E-10	0.002068	0.370396	8.63E-05	39.0164
Bread intake || id:ukb-b-11348	452236	rs11628639	C	T	0.243333	-0.013495	6.20E-09	0.002322	0.243333	7.47E-05	33.76931
Bread intake || id:ukb-b-11348	452236	rs9323989	C	T	0.379169	-0.011614	1.60E-08	0.002055	0.379169	7.06E-05	31.92728
Bread intake || id:ukb-b-11348	452236	rs28406095	A	G	0.461729	-0.010949	4.40E-08	0.002	0.461729	6.63E-05	29.96642
Bread intake || id:ukb-b-11348	452236	rs4984685	A	G	0.201006	0.013585	4.40E-08	0.002481	0.201006	6.63E-05	29.98589
Bread intake || id:ukb-b-11348	452236	rs62091167	C	A	0.215931	-0.01384	1.20E-08	0.002425	0.215931	7.20E-05	32.56506
Bread intake || id:ukb-b-11348	452236	rs656817	G	A	0.334442	-0.012688	1.80E-09	0.002109	0.334442	8.00E-05	36.18532
Bread intake || id:ukb-b-11348	452236	rs7276867	C	G	0.541677	0.011348	1.50E-08	0.002003	0.458323	7.10E-05	32.09777
Cereal intake || id:ukb-b-15926	441640	rs10857964	C	T	0.205117	0.014087	1.70E-10	0.002205	0.205117	9.24E-05	40.8014
Cereal intake || id:ukb-b-15926	441640	rs12354267	C	T	0.309145	0.011647	1.70E-09	0.001933	0.309145	8.22E-05	36.31443
Cereal intake || id:ukb-b-15926	441640	rs112780312	A	G	0.274969	-0.012148	1.80E-09	0.002019	0.274969	8.20E-05	36.20757
Cereal intake || id:ukb-b-15926	441640	rs184643	A	G	0.56672	-0.012165	1.60E-11	0.001805	0.43328	1.03E-04	45.4375
Cereal intake || id:ukb-b-15926	441640	rs6545770	T	A	0.748101	-0.013728	2.70E-11	0.00206	0.251899	1.01E-04	44.39206
Cereal intake || id:ukb-b-15926	441640	rs4988235	A	G	0.736893	0.0114	1.50E-08	0.002012	0.263107	7.27E-05	32.09038
Cereal intake || id:ukb-b-15926	441640	rs67723420	A	T	0.376335	0.010525	1.20E-08	0.001847	0.376335	7.36E-05	32.4855
Cereal intake || id:ukb-b-15926	441640	rs7619139	A	T	0.588533	-0.016951	9.70E-21	0.001815	0.411467	1.97E-04	87.22485
Cereal intake || id:ukb-b-15926	441640	rs9846396	T	C	0.441556	0.011967	3.00E-11	0.0018	0.441556	1.00E-04	44.18787
Cereal intake || id:ukb-b-15926	441640	rs11097340	T	C	0.399592	-0.011533	2.10E-10	0.001815	0.399592	9.14E-05	40.37892
Cereal intake || id:ukb-b-15926	441640	rs3115230	A	C	0.752001	-0.011478	3.00E-08	0.002071	0.247999	6.95E-05	30.70667
Cereal intake || id:ukb-b-15926	441640	rs11940694	G	A	0.604068	-0.012665	5.00E-12	0.001834	0.395932	1.08E-04	47.67229
Cereal intake || id:ukb-b-15926	441640	rs10057775	C	T	0.893563	0.020029	4.50E-12	0.002894	0.106437	1.08E-04	47.90695
Cereal intake || id:ukb-b-15926	441640	rs79642906	A	G	0.083314	-0.018158	1.90E-08	0.003228	0.083314	7.16E-05	31.64022
Cereal intake || id:ukb-b-15926	441640	rs1853931	A	G	0.531294	-0.011337	3.80E-10	0.00181	0.468706	8.88E-05	39.22295
Cereal intake || id:ukb-b-15926	441640	rs6918737	A	T	0.234488	0.013733	7.60E-11	0.00211	0.234488	9.59E-05	42.36733

Cereal intake || id:ukb-b-15926	441640	rs2817377	A	G	0.537948	0.009901	3.10E-08	0.001789	0.462052	6.93E-05	30.6169
Cereal intake || id:ukb-b-15926	441640	rs2504706	C	T	0.234682	0.018179	5.30E-18	0.002102	0.234682	1.69E-04	74.77044
Cereal intake || id:ukb-b-15926	441640	rs9374896	T	C	0.466274	0.017527	1.30E-22	0.001792	0.466274	2.17E-04	95.69342
Cereal intake || id:ukb-b-15926	441640	rs4410790	C	T	0.630668	-0.010914	3.40E-09	0.001847	0.369332	7.91E-05	34.91967
Cereal intake || id:ukb-b-15926	441640	rs62442924	T	C	0.194276	0.012731	1.70E-08	0.002257	0.194276	7.20E-05	31.81402
Cereal intake || id:ukb-b-15926	441640	rs13234131	G	A	0.128369	0.017012	1.60E-10	0.00266	0.128369	9.26E-05	40.8945
Cereal intake || id:ukb-b-15926	441640	rs9987289	G	A	0.908767	0.017868	7.80E-09	0.003095	0.091233	7.55E-05	33.33557
Cereal intake || id:ukb-b-15926	441640	rs4739095	A	G	0.765732	-0.012866	9.90E-10	0.002105	0.234268	8.46E-05	37.34728
Cereal intake || id:ukb-b-15926	441640	rs2927238	G	T	0.613181	0.010247	2.10E-08	0.001829	0.386819	7.11E-05	31.39183
Cereal intake || id:ukb-b-15926	441640	rs2799849	T	C	0.678123	-0.012329	9.80E-11	0.001906	0.321877	9.48E-05	41.85275
Cereal intake || id:ukb-b-15926	441640	rs7040561	A	T	0.850635	-0.016269	1.10E-10	0.00252	0.149365	9.44E-05	41.67591
Cereal intake || id:ukb-b-15926	441640	rs491711	C	A	0.311975	0.011689	1.50E-09	0.001934	0.311975	8.27E-05	36.53889
Cereal intake || id:ukb-b-15926	441640	rs2450126	G	A	0.156746	-0.014902	1.30E-09	0.002457	0.156746	8.33E-05	36.79431
Cereal intake || id:ukb-b-15926	441640	rs10837531	G	C	0.455002	0.010784	2.00E-09	0.001797	0.455002	8.15E-05	36.01573
Cereal intake || id:ukb-b-15926	441640	rs11038810	G	A	0.644091	0.011134	2.30E-09	0.001863	0.355909	8.09E-05	35.70932
Cereal intake || id:ukb-b-15926	441640	rs627185	G	C	0.544427	-0.010827	1.50E-09	0.001791	0.455573	8.28E-05	36.55371
Cereal intake || id:ukb-b-15926	441640	rs2472297	T	C	0.261458	-0.015852	4.50E-15	0.002022	0.261458	1.39E-04	61.46674
Cereal intake || id:ukb-b-15926	441640	rs1104608	C	G	0.426239	0.010863	2.30E-09	0.001818	0.426239	8.08E-05	35.68588
Cereal intake || id:ukb-b-15926	441640	rs68136852	A	C	0.152389	-0.014122	1.20E-08	0.002478	0.152389	7.35E-05	32.46623
Cereal intake || id:ukb-b-15926	441640	rs3859193	A	T	0.470074	-0.010326	9.50E-09	0.001799	0.470074	7.46E-05	32.93536
Cereal intake || id:ukb-b-15926	441640	rs8097544	G	A	0.145206	-0.024637	3.20E-22	0.002541	0.145206	2.13E-04	94.00045
Cereal intake || id:ukb-b-15926	441640	rs4797242	A	C	0.297207	0.011431	4.50E-09	0.001949	0.297207	7.79E-05	34.40385
Cereal intake || id:ukb-b-15926	441640	rs11670024	G	A	0.115508	0.016011	1.10E-08	0.002799	0.115508	7.41E-05	32.71184
Cereal intake || id:ukb-b-15926	441640	rs6510177	C	T	0.805589	-0.013035	1.20E-08	0.002287	0.194411	7.35E-05	32.47486
Cereal intake || id:ukb-b-15926	441640	rs78854891	C	T	0.065728	0.022141	1.10E-09	0.003633	0.065728	8.41E-05	37.13958
Cereal intake || id:ukb-b-15926	441640	rs56131196	A	G	0.188724	0.018002	2.70E-15	0.002277	0.188724	1.42E-04	62.51034
Cereal intake || id:ukb-b-15926	441640	rs838133	G	A	0.549201	-0.020652	3.90E-29	0.001843	0.450799	2.84E-04	125.5176
Cheese intake || id:ukb-b-1489	451486	rs78876700	A	G	0.137431	0.018097	3.40E-08	0.003279	0.137431	6.75E-05	30.46713
Cheese intake || id:ukb-b-1489	451486	rs531358	T	C	0.649768	0.013167	1.80E-08	0.002337	0.350232	7.03E-05	31.73312
Cheese intake || id:ukb-b-1489	451486	rs2802530	A	G	0.876502	0.018627	4.20E-08	0.003397	0.123498	6.66E-05	30.06519
Cheese intake || id:ukb-b-1489	451486	rs6685323	T	C	0.309293	-0.013189	4.80E-08	0.002416	0.309293	6.60E-05	29.80097
Cheese intake || id:ukb-b-1489	451486	rs2339928	A	G	0.704069	0.014858	1.20E-09	0.002445	0.295931	8.18E-05	36.92927

Cheese intake || id:ukb-b-1489	451486	rs12475594	G	A	0.178459	0.016007	4.40E-08	0.002925	0.178459	6.63E-05	29.94533
Cheese intake || id:ukb-b-1489	451486	rs504675	T	C	0.352634	0.027442	1.00E-31	0.002342	0.352634	3.04E-04	137.3145
Cheese intake || id:ukb-b-1489	451486	rs72970243	A	G	0.12044	0.022201	6.70E-11	0.003402	0.12044	9.43E-05	42.59565
Cheese intake || id:ukb-b-1489	451486	rs1514755	G	A	0.239596	0.016375	3.90E-10	0.002617	0.239596	8.67E-05	39.16612
Cheese intake || id:ukb-b-1489	451486	rs79184944	A	T	0.134346	0.019602	2.40E-09	0.003285	0.134346	7.89E-05	35.61478
Cheese intake || id:ukb-b-1489	451486	rs4296548	G	T	0.609594	0.013025	1.20E-08	0.002288	0.390406	7.18E-05	32.41009
Cheese intake || id:ukb-b-1489	451486	rs62245792	A	T	0.150049	-0.017934	1.40E-08	0.003163	0.150049	7.12E-05	32.14491
Cheese intake || id:ukb-b-1489	451486	rs77742462	G	A	0.020525	-0.047467	9.80E-09	0.008278	0.020525	7.28E-05	32.87933
Cheese intake || id:ukb-b-1489	451486	rs2352974	T	C	0.48979	-0.014497	1.00E-10	0.002243	0.48979	9.25E-05	41.78162
Cheese intake || id:ukb-b-1489	451486	rs6774906	C	A	0.040653	0.031629	2.50E-08	0.005673	0.040653	6.89E-05	31.08942
Cheese intake || id:ukb-b-1489	451486	rs4681981	A	C	0.469086	-0.012436	2.90E-08	0.002241	0.469086	6.82E-05	30.78941
Cheese intake || id:ukb-b-1489	451486	rs4860341	C	T	0.928742	0.024365	2.20E-08	0.004352	0.071258	6.94E-05	31.35134
Cheese intake || id:ukb-b-1489	451486	rs73096946	C	T	0.157396	-0.020591	1.90E-11	0.003067	0.157396	9.99E-05	45.08626
Cheese intake || id:ukb-b-1489	451486	rs13107325	T	C	0.074693	-0.029164	7.00E-12	0.004253	0.074693	1.04E-04	47.02504
Cheese intake || id:ukb-b-1489	451486	rs10938397	G	A	0.434462	-0.012707	1.80E-08	0.002258	0.434462	7.02E-05	31.68339
Cheese intake || id:ukb-b-1489	451486	rs4692708	C	A	0.252659	0.01473	1.30E-08	0.002589	0.252659	7.17E-05	32.36788
Cheese intake || id:ukb-b-1489	451486	rs26579	C	G	0.586217	-0.012801	2.40E-08	0.002294	0.413783	6.89E-05	31.12981
Cheese intake || id:ukb-b-1489	451486	rs6873324	C	A	0.425802	-0.012477	3.90E-08	0.002271	0.425802	6.69E-05	30.18975
Cheese intake || id:ukb-b-1489	451486	rs9504123	C	A	0.274743	0.014171	1.50E-08	0.002504	0.274743	7.09E-05	32.02956
Cheese intake || id:ukb-b-1489	451486	rs975303	G	A	0.181313	0.021276	2.50E-13	0.002907	0.181313	1.19E-04	53.5642
Cheese intake || id:ukb-b-1489	451486	rs1931805	C	T	0.500062	0.012636	1.60E-08	0.002236	0.499938	7.07E-05	31.93959
Cheese intake || id:ukb-b-1489	451486	rs113367286	T	C	0.278467	0.01518	1.30E-09	0.0025	0.278467	8.17E-05	36.87199
Cheese intake || id:ukb-b-1489	451486	rs34198643	T	C	0.224165	-0.016701	4.50E-10	0.002679	0.224165	8.61E-05	38.87431
Cheese intake || id:ukb-b-1489	451486	rs12672200	A	G	0.325794	-0.013763	9.00E-09	0.002395	0.325794	7.32E-05	33.03681
Cheese intake || id:ukb-b-1489	451486	rs9649582	T	A	0.317256	-0.014617	1.40E-09	0.002412	0.317256	8.14E-05	36.73715
Cheese intake || id:ukb-b-1489	451486	rs7012814	A	G	0.473966	-0.018516	2.10E-16	0.002254	0.473966	1.49E-04	67.48939
Cheese intake || id:ukb-b-1489	451486	rs7386207	T	C	0.563517	-0.012497	3.60E-08	0.002269	0.436483	6.72E-05	30.33002
Cheese intake || id:ukb-b-1489	451486	rs13257887	C	T	0.358942	0.016181	2.70E-10	0.002562	0.358942	8.83E-05	39.89213
Cheese intake || id:ukb-b-1489	451486	rs3911016	G	T	0.120938	0.021357	5.30E-10	0.00344	0.120938	8.54E-05	38.54776
Cheese intake || id:ukb-b-1489	451486	rs4503172	T	C	0.608301	0.01296	1.60E-08	0.002293	0.391699	7.07E-05	31.94281
Cheese intake || id:ukb-b-1489	451486	rs1806771	G	T	0.087876	-0.022147	4.10E-08	0.004035	0.087876	6.67E-05	30.12514
Cheese intake || id:ukb-b-1489	451486	rs73335955	C	T	0.05334	0.027773	2.40E-08	0.004978	0.05334	6.89E-05	31.12286

Cheese intake || id:ukb-b-1489	451486	rs10896050	T	G	0.193171	-0.01847	7.20E-11	0.002834	0.193171	9.40E-05	42.46119
Cheese intake || id:ukb-b-1489	451486	rs67238148	T	G	0.217471	0.016543	1.10E-09	0.002714	0.217471	8.23E-05	37.14625
Cheese intake || id:ukb-b-1489	451486	rs7936836	A	C	0.417538	0.015903	2.60E-12	0.002273	0.417538	1.08E-04	48.96197
Cheese intake || id:ukb-b-1489	451486	rs73024305	C	G	0.054789	0.032537	4.00E-11	0.004927	0.054789	9.66E-05	43.60781
Cheese intake || id:ukb-b-1489	451486	rs12786959	T	A	0.196267	-0.016071	1.20E-08	0.00282	0.196267	7.19E-05	32.4813
Cheese intake || id:ukb-b-1489	451486	rs524468	G	A	0.260633	-0.014242	2.40E-08	0.002552	0.260633	6.90E-05	31.15427
Cheese intake || id:ukb-b-1489	451486	rs1024853	G	C	0.437902	-0.012863	1.30E-08	0.002262	0.437902	7.16E-05	32.32683
Cheese intake || id:ukb-b-1489	451486	rs7298331	C	A	0.604531	-0.013175	1.10E-08	0.002306	0.395469	7.23E-05	32.6457
Cheese intake || id:ukb-b-1489	451486	rs12296440	A	G	0.169681	0.018794	2.80E-10	0.00298	0.169681	8.81E-05	39.77928
Cheese intake || id:ukb-b-1489	451486	rs61953351	T	G	0.250366	0.014593	1.50E-08	0.00258	0.250366	7.09E-05	31.99434
Cheese intake || id:ukb-b-1489	451486	rs1073242	A	G	0.553825	0.015728	6.70E-12	0.002292	0.446175	1.04E-04	47.10669
Cheese intake || id:ukb-b-1489	451486	rs11620149	C	T	0.143308	-0.017675	3.60E-08	0.003209	0.143308	6.72E-05	30.34508
Cheese intake || id:ukb-b-1489	451486	rs17115145	T	C	0.401282	-0.012881	1.80E-08	0.00229	0.401282	7.01E-05	31.64945
Cheese intake || id:ukb-b-1489	451486	rs35270670	G	A	0.217962	0.016379	1.50E-09	0.00271	0.217962	8.09E-05	36.5375
Cheese intake || id:ukb-b-1489	451486	rs4776970	T	A	0.357982	0.015409	3.50E-11	0.002327	0.357982	9.71E-05	43.85372
Cheese intake || id:ukb-b-1489	451486	rs12447542	A	G	0.12558	0.019748	6.80E-09	0.003407	0.12558	7.44E-05	33.596
Cheese intake || id:ukb-b-1489	451486	rs61734410	T	C	0.255213	0.016654	2.20E-10	0.002623	0.255213	8.93E-05	40.31885
Cheese intake || id:ukb-b-1489	451486	rs62034322	A	G	0.379848	-0.013936	1.40E-09	0.0023	0.379848	8.13E-05	36.71473
Cheese intake || id:ukb-b-1489	451486	rs71386942	A	C	0.268947	0.014455	9.90E-09	0.002522	0.268947	7.28E-05	32.85118
Cheese intake || id:ukb-b-1489	451486	rs11649653	G	C	0.38208	0.013849	1.50E-09	0.002292	0.38208	8.09E-05	36.51672
Cheese intake || id:ukb-b-1489	451486	rs919109	C	G	0.138752	0.019938	7.90E-10	0.003243	0.138752	8.37E-05	37.79187
Cheese intake || id:ukb-b-1489	451486	rs2854175	A	C	0.257474	0.016994	3.70E-11	0.002569	0.257474	9.69E-05	43.77147
Cheese intake || id:ukb-b-1489	451486	rs12951057	G	C	0.165627	-0.021156	3.60E-12	0.003044	0.165627	1.07E-04	48.30945
Cheese intake || id:ukb-b-1489	451486	rs2960578	G	T	0.496281	0.017028	2.60E-14	0.002236	0.496281	1.28E-04	57.9983
Cheese intake || id:ukb-b-1489	451486	rs1434511	T	C	0.455286	0.012965	9.50E-09	0.002259	0.455286	7.30E-05	32.94632
Cheese intake || id:ukb-b-1489	451486	rs1291145	C	T	0.685848	-0.02025	4.40E-17	0.00241	0.314152	1.56E-04	70.5828
Cheese intake || id:ukb-b-1489	451486	rs6126641	A	G	0.336196	0.013226	3.30E-08	0.002395	0.336196	6.75E-05	30.49812
Cheese intake || id:ukb-b-1489	451486	rs62236533	A	G	0.108791	0.024762	1.10E-11	0.003647	0.108791	1.02E-04	46.10514
Salt added to food || id:ukb-b-8121	462630	rs12563932	C	A	0.029478	-0.028493	4.50E-08	0.00521	0.029478	6.46E-05	29.90905
Salt added to food || id:ukb-b-8121	462630	rs10752999	C	A	0.702035	0.012762	3.50E-11	0.001927	0.297965	9.48E-05	43.85355
Salt added to food || id:ukb-b-8121	462630	rs12094804	G	A	0.061754	0.020506	2.00E-08	0.003656	0.061754	6.80E-05	31.46251
Salt added to food || id:ukb-b-8121	462630	rs11210985	A	G	0.3791	-0.015657	7.20E-18	0.001818	0.3791	1.60E-04	74.16023

Salt added to food || id:ukb-b-8121	462630	rs6695915	G	A	0.095551	-0.016629	2.90E-08	0.002998	0.095551	6.65E-05	30.75928
Salt added to food || id:ukb-b-8121	462630	rs1008078	T	C	0.395512	0.01166	1.10E-10	0.001806	0.395512	9.00E-05	41.65832
Salt added to food || id:ukb-b-8121	462630	rs528301	A	G	0.554846	0.015261	6.60E-18	0.00177	0.445154	1.61E-04	74.33145
Salt added to food || id:ukb-b-8121	462630	rs976179	T	A	0.485324	0.010067	1.20E-08	0.001765	0.485324	7.03E-05	32.53711
Salt added to food || id:ukb-b-8121	462630	rs55897719	A	C	0.316817	0.013502	1.10E-12	0.001896	0.316817	1.10E-04	50.71356
Salt added to food || id:ukb-b-8121	462630	rs7591518	C	T	0.298453	-0.020772	5.10E-27	0.00193	0.298453	2.50E-04	115.8648
Salt added to food || id:ukb-b-8121	462630	rs11126666	A	G	0.255562	-0.011988	2.90E-09	0.002019	0.255562	7.62E-05	35.25147
Salt added to food || id:ukb-b-8121	462630	rs2263636	C	A	0.719001	0.011256	1.00E-08	0.001965	0.280999	7.10E-05	32.82684
Salt added to food || id:ukb-b-8121	462630	rs13400612	G	C	0.246906	0.020285	3.00E-23	0.002043	0.246906	2.13E-04	98.62758
Salt added to food || id:ukb-b-8121	462630	rs12988960	C	G	0.315972	0.014981	2.80E-15	0.001896	0.315972	1.35E-04	62.40112
Salt added to food || id:ukb-b-8121	462630	rs264932	G	A	0.605889	-0.01042	7.30E-09	0.001802	0.394111	7.23E-05	33.44353
Salt added to food || id:ukb-b-8121	462630	rs7581335	T	A	0.140518	0.014931	3.80E-09	0.002533	0.140518	7.51E-05	34.74112
Salt added to food || id:ukb-b-8121	462630	rs17805497	C	T	0.345071	0.012482	1.50E-11	0.00185	0.345071	9.84E-05	45.51017
Salt added to food || id:ukb-b-8121	462630	rs13084934	T	A	0.494691	-0.009676	4.40E-08	0.001767	0.494691	6.48E-05	29.96974
Salt added to food || id:ukb-b-8121	462630	rs6776248	C	T	0.26336	0.011341	1.40E-08	0.001998	0.26336	6.96E-05	32.21964
Salt added to food || id:ukb-b-8121	462630	rs6780346	T	C	0.620997	-0.016837	1.90E-20	0.001817	0.379003	1.86E-04	85.84466
Salt added to food || id:ukb-b-8121	462630	rs400750	G	T	0.616712	0.016712	3.30E-20	0.001815	0.383288	1.83E-04	84.81246
Salt added to food || id:ukb-b-8121	462630	rs2736748	G	A	0.789193	0.028012	1.80E-38	0.00216	0.210807	3.64E-04	168.2479
Salt added to food || id:ukb-b-8121	462630	rs73040343	G	A	0.381434	-0.010351	1.20E-08	0.001814	0.381434	7.04E-05	32.55427
Salt added to food || id:ukb-b-8121	462630	rs736935	T	C	0.336413	0.015688	6.80E-17	0.001879	0.336413	1.51E-04	69.72772
Salt added to food || id:ukb-b-8121	462630	rs9843358	T	C	0.171903	0.018953	6.20E-16	0.002344	0.171903	1.41E-04	65.36599
Salt added to food || id:ukb-b-8121	462630	rs9835772	T	A	0.243664	0.011451	2.40E-08	0.002052	0.243664	6.73E-05	31.13726
Salt added to food || id:ukb-b-8121	462630	rs11130206	G	C	0.432321	-0.012032	1.20E-11	0.001776	0.432321	9.93E-05	45.92473
Salt added to food || id:ukb-b-8121	462630	rs6804929	A	G	0.105016	0.016706	7.40E-09	0.002889	0.105016	7.23E-05	33.43316
Salt added to food || id:ukb-b-8121	462630	rs6443950	T	A	0.631504	-0.014205	8.50E-15	0.001831	0.368496	1.30E-04	60.21301
Salt added to food || id:ukb-b-8121	462630	rs7673170	A	G	0.395273	-0.012597	2.70E-12	0.001801	0.395273	1.06E-04	48.90183
Salt added to food || id:ukb-b-8121	462630	rs12501838	T	A	0.23366	0.014667	1.80E-12	0.002082	0.23366	1.07E-04	49.64757
Salt added to food || id:ukb-b-8121	462630	rs7670308	A	G	0.409195	-0.01164	1.00E-10	0.001801	0.409195	9.03E-05	41.75928
Salt added to food || id:ukb-b-8121	462630	rs4860797	A	G	0.601276	0.011667	1.10E-10	0.001808	0.398724	9.00E-05	41.65791
Salt added to food || id:ukb-b-8121	462630	rs34906832	G	A	0.140896	0.015098	2.40E-09	0.00253	0.140896	7.70E-05	35.60811
Salt added to food || id:ukb-b-8121	462630	rs526210	A	G	0.542868	0.010095	2.80E-08	0.001819	0.457132	6.66E-05	30.80944
Salt added to food || id:ukb-b-8121	462630	rs3890316	A	G	0.249954	-0.011333	2.80E-08	0.002042	0.249954	6.66E-05	30.81051

Salt added to food || id:ukb-b-8121	462630	rs13131880	C	T	0.220413	-0.012619	3.00E-09	0.002128	0.220413	7.60E-05	35.18207
Salt added to food || id:ukb-b-8121	462630	rs12658060	C	T	0.222971	-0.027415	2.50E-38	0.002118	0.222971	3.62E-04	167.5956
Salt added to food || id:ukb-b-8121	462630	rs868720	C	G	0.305451	0.015994	9.60E-17	0.001925	0.305451	1.49E-04	69.05329
Salt added to food || id:ukb-b-8121	462630	rs33137	T	C	0.493871	-0.012084	9.40E-12	0.001773	0.493871	1.00E-04	46.44312
Salt added to food || id:ukb-b-8121	462630	rs1823011	G	A	0.659939	-0.010226	3.90E-08	0.001861	0.340061	6.52E-05	30.18528
Salt added to food || id:ukb-b-8121	462630	rs4912891	C	T	0.691882	0.010655	3.00E-08	0.001923	0.308118	6.64E-05	30.70236
Salt added to food || id:ukb-b-8121	462630	rs4235642	G	A	0.379691	0.012294	1.30E-11	0.001816	0.379691	9.90E-05	45.80386
Salt added to food || id:ukb-b-8121	462630	rs6887291	G	T	0.636126	0.012676	5.10E-12	0.001836	0.363874	1.03E-04	47.63925
Salt added to food || id:ukb-b-8121	462630	rs2339234	A	G	0.683186	-0.017642	1.60E-20	0.0019	0.316814	1.86E-04	86.20457
Salt added to food || id:ukb-b-8121	462630	rs9375448	T	A	0.492981	-0.011679	3.40E-11	0.001762	0.492981	9.50E-05	43.95038
Salt added to food || id:ukb-b-8121	462630	rs35099536	C	A	0.08461	0.025959	5.80E-16	0.003207	0.08461	1.42E-04	65.49881
Salt added to food || id:ukb-b-8121	462630	rs4236065	C	T	0.255885	-0.011171	3.80E-08	0.002031	0.255885	6.54E-05	30.26694
Salt added to food || id:ukb-b-8121	462630	rs28366169	A	G	0.077319	-0.020848	2.60E-10	0.003299	0.077319	8.63E-05	39.92524
Salt added to food || id:ukb-b-8121	462630	rs2506738	G	A	0.401141	-0.015101	5.00E-17	0.001801	0.401141	1.52E-04	70.33383
Salt added to food || id:ukb-b-8121	462630	rs9278020	A	G	0.10619	0.023222	4.80E-16	0.002862	0.10619	1.42E-04	65.85888
Salt added to food || id:ukb-b-8121	462630	rs2463710	A	T	0.753885	0.011952	4.90E-08	0.002191	0.246115	6.43E-05	29.7537
Salt added to food || id:ukb-b-8121	462630	rs1726866	A	G	0.552077	0.026544	7.70E-51	0.00177	0.447923	4.86E-04	224.8973
Salt added to food || id:ukb-b-8121	462630	rs11761254	T	C	0.136978	-0.017681	3.80E-12	0.002546	0.136978	1.04E-04	48.22963
Salt added to food || id:ukb-b-8121	462630	rs2457427	C	T	0.863856	-0.016582	1.10E-10	0.002569	0.136144	9.00E-05	41.64746
Salt added to food || id:ukb-b-8121	462630	rs6987313	C	T	0.520156	0.009907	2.00E-08	0.001766	0.479844	6.80E-05	31.47767
Salt added to food || id:ukb-b-8121	462630	rs4739105	C	T	0.787781	0.015465	1.30E-12	0.00218	0.212219	1.09E-04	50.31784
Salt added to food || id:ukb-b-8121	462630	rs10971930	C	T	0.127418	0.015501	4.50E-09	0.002644	0.127418	7.43E-05	34.38301
Salt added to food || id:ukb-b-8121	462630	rs586716	A	G	0.414681	0.014367	1.10E-15	0.001793	0.414681	1.39E-04	64.1806
Salt added to food || id:ukb-b-8121	462630	rs7465705	A	G	0.384538	-0.011847	6.80E-11	0.001816	0.384538	9.20E-05	42.56707
Salt added to food || id:ukb-b-8121	462630	rs7021360	A	C	0.388225	-0.010119	2.30E-08	0.00181	0.388225	6.75E-05	31.25247
Salt added to food || id:ukb-b-8121	462630	rs10128297	T	C	0.402787	-0.010555	4.50E-09	0.0018	0.402787	7.43E-05	34.38221
Salt added to food || id:ukb-b-8121	462630	rs10883796	A	G	0.294358	0.011441	3.30E-09	0.001934	0.294358	7.56E-05	34.98445
Salt added to food || id:ukb-b-8121	462630	rs4595499	T	C	0.391888	-0.017278	1.20E-21	0.001808	0.391888	1.97E-04	91.34669
Salt added to food || id:ukb-b-8121	462630	rs4948275	T	C	0.481851	0.012715	7.40E-13	0.001773	0.481851	1.11E-04	51.4467
Salt added to food || id:ukb-b-8121	462630	rs10736951	C	G	0.635537	-0.011362	7.00E-10	0.001843	0.364463	8.22E-05	38.0091
Salt added to food || id:ukb-b-8121	462630	rs9667150	A	G	0.559523	0.012589	1.50E-12	0.001781	0.440477	1.08E-04	49.98594
Salt added to food || id:ukb-b-8121	462630	rs329670	C	T	0.870289	-0.015292	6.30E-09	0.002633	0.129711	7.29E-05	33.73487

Salt added to food || id:ukb-b-8121	462630	rs35271178	T	C	0.588507	0.010244	1.10E-08	0.001791	0.411493	7.07E-05	32.71749
Salt added to food || id:ukb-b-8121	462630	rs7927679	T	C	0.504024	0.014954	2.30E-17	0.001764	0.495976	1.55E-04	71.86538
Salt added to food || id:ukb-b-8121	462630	rs12789951	T	C	0.411036	-0.011717	7.00E-11	0.001797	0.411036	9.19E-05	42.52002
Salt added to food || id:ukb-b-8121	462630	rs7110845	G	A	0.579174	-0.011538	1.10E-10	0.001786	0.420826	9.02E-05	41.71909
Salt added to food || id:ukb-b-8121	462630	rs35702851	C	G	0.204203	0.012196	2.60E-08	0.002191	0.204203	6.70E-05	30.9984
Salt added to food || id:ukb-b-8121	462630	rs11022746	G	T	0.681941	0.011827	6.30E-10	0.001913	0.318059	8.26E-05	38.215
Salt added to food || id:ukb-b-8121	462630	rs99780	T	C	0.350239	0.010907	3.60E-09	0.001848	0.350239	7.53E-05	34.85097
Salt added to food || id:ukb-b-8121	462630	rs667128	T	C	0.126584	0.015426	6.00E-09	0.002652	0.126584	7.31E-05	33.82501
Salt added to food || id:ukb-b-8121	462630	rs1895951	C	T	0.220794	-0.011691	4.80E-08	0.002142	0.220794	6.44E-05	29.78579
Salt added to food || id:ukb-b-8121	462630	rs324018	G	T	0.613528	0.01001	3.30E-08	0.001812	0.386472	6.60E-05	30.533
Salt added to food || id:ukb-b-8121	462630	rs12579997	C	G	0.170455	0.016031	8.60E-12	0.002348	0.170455	1.01E-04	46.62127
Salt added to food || id:ukb-b-8121	462630	rs1045411	T	C	0.268305	-0.015066	4.00E-14	0.001993	0.268305	1.24E-04	57.17038
Salt added to food || id:ukb-b-8121	462630	rs9569747	G	T	0.298073	-0.012559	8.60E-11	0.001936	0.298073	9.10E-05	42.10567
Salt added to food || id:ukb-b-8121	462630	rs9317406	T	C	0.376115	-0.010702	4.40E-09	0.001824	0.376115	7.44E-05	34.42817
Salt added to food || id:ukb-b-8121	462630	rs7982263	C	T	0.581981	0.010608	2.90E-09	0.001788	0.418019	7.61E-05	35.22133
Salt added to food || id:ukb-b-8121	462630	rs10140751	T	G	0.245951	-0.011275	3.60E-08	0.002047	0.245951	6.56E-05	30.34362
Salt added to food || id:ukb-b-8121	462630	rs4981196	C	A	0.635898	-0.011456	3.30E-10	0.001822	0.364102	8.54E-05	39.51356
Salt added to food || id:ukb-b-8121	462630	rs8022455	C	T	0.54702	-0.011142	3.40E-10	0.001774	0.45298	8.53E-05	39.44561
Salt added to food || id:ukb-b-8121	462630	rs2693687	T	C	0.393736	0.011628	1.70E-10	0.00182	0.393736	8.82E-05	40.79751
Salt added to food || id:ukb-b-8121	462630	rs961044	T	C	0.857621	-0.014008	3.00E-08	0.002527	0.142379	6.64E-05	30.72052
Salt added to food || id:ukb-b-8121	462630	rs2521501	T	A	0.322086	-0.011395	2.00E-09	0.0019	0.322086	7.77E-05	35.95604
Salt added to food || id:ukb-b-8121	462630	rs8040685	T	C	0.884465	-0.015407	2.30E-08	0.002758	0.115535	6.74E-05	31.20379
Salt added to food || id:ukb-b-8121	462630	rs491907	G	A	0.511555	0.010986	4.60E-10	0.001763	0.488445	8.39E-05	38.83436
Salt added to food || id:ukb-b-8121	462630	rs6416794	C	T	0.798724	-0.012	4.90E-08	0.0022	0.201276	6.43E-05	29.74157
Salt added to food || id:ukb-b-8121	462630	rs1728779	G	A	0.436092	0.009871	3.50E-08	0.001789	0.436092	6.58E-05	30.4357
Salt added to food || id:ukb-b-8121	462630	rs11075194	G	A	0.425797	-0.009817	4.00E-08	0.001787	0.425797	6.52E-05	30.16224
Salt added to food || id:ukb-b-8121	462630	rs2547040	C	G	0.805074	-0.012708	1.20E-08	0.002229	0.194926	7.02E-05	32.49164
Salt added to food || id:ukb-b-8121	462630	rs72807804	T	C	0.218782	0.012064	1.70E-08	0.002139	0.218782	6.88E-05	31.8105
Salt added to food || id:ukb-b-8121	462630	rs35142265	G	A	0.218202	0.015013	2.10E-12	0.002136	0.218202	1.07E-04	49.38594
Salt added to food || id:ukb-b-8121	462630	rs11082431	T	C	0.301904	0.010565	3.80E-08	0.001921	0.301904	6.54E-05	30.25454
Salt added to food || id:ukb-b-8121	462630	rs62098445	A	C	0.318657	-0.015864	1.00E-16	0.001911	0.318657	1.49E-04	68.87957
Salt added to food || id:ukb-b-8121	462630	rs8097544	G	A	0.145366	0.01403	2.40E-08	0.002513	0.145366	6.74E-05	31.1755

Salt added to food || id:ukb-b-8121	462630	rs4799949	T	C	0.667226	-0.01098	4.50E-09	0.001872	0.332774	7.44E-05	34.39976
Salt added to food || id:ukb-b-8121	462630	rs2852348	G	A	0.458696	-0.013117	1.60E-13	0.001777	0.458696	1.18E-04	54.45991
Salt added to food || id:ukb-b-8121	462630	rs429358	C	T	0.154168	-0.015398	2.90E-10	0.002442	0.154168	8.59E-05	39.7581
Salt added to food || id:ukb-b-8121	462630	rs2835623	T	C	0.04766	0.022677	4.40E-08	0.004144	0.04766	6.47E-05	29.95099
Salt added to food || id:ukb-b-8121	462630	rs9611875	G	A	0.039616	0.066201	1.30E-48	0.004519	0.039616	4.64E-04	214.6348
Salt added to food || id:ukb-b-8121	462630	rs2899345	C	T	0.504298	0.009887	2.50E-08	0.001773	0.495702	6.72E-05	31.09423
Tea intake || id:ukb-b-6066	447485	rs11587444	G	A	0.393464	0.014033	1.00E-10	0.002171	0.393464	9.34E-05	41.78834
Tea intake || id:ukb-b-6066	447485	rs11164870	G	C	0.604574	-0.01196	4.20E-08	0.002182	0.395426	6.71E-05	30.03673
Tea intake || id:ukb-b-6066	447485	rs56188862	C	T	0.387454	-0.015757	4.30E-13	0.002175	0.387454	1.17E-04	52.49711
Tea intake || id:ukb-b-6066	447485	rs1156588	G	A	0.210071	-0.015454	2.90E-09	0.002603	0.210071	7.87E-05	35.24099
Tea intake || id:ukb-b-6066	447485	rs57462170	A	G	0.108773	0.019151	1.90E-08	0.003406	0.108773	7.07E-05	31.62011
Tea intake || id:ukb-b-6066	447485	rs2117137	G	A	0.405148	0.012995	1.70E-09	0.002156	0.405148	8.12E-05	36.33797
Tea intake || id:ukb-b-6066	447485	rs1481012	G	A	0.112209	-0.026244	5.30E-15	0.003356	0.112209	1.37E-04	61.14725
Tea intake || id:ukb-b-6066	447485	rs34619	A	G	0.430905	0.011712	4.30E-08	0.002138	0.430905	6.71E-05	30.02104
Tea intake || id:ukb-b-6066	447485	rs72797284	G	A	0.270797	-0.017115	7.00E-13	0.002384	0.270797	1.15E-04	51.55792
Tea intake || id:ukb-b-6066	447485	rs7757102	G	A	0.555426	-0.011804	3.10E-08	0.002133	0.444574	6.84E-05	30.62381
Tea intake || id:ukb-b-6066	447485	rs2478875	G	A	0.208758	0.021894	5.10E-17	0.002611	0.208758	1.57E-04	70.29913
Tea intake || id:ukb-b-6066	447485	rs149805207	G	A	0.008538	-0.071934	1.10E-08	0.012582	0.008538	7.30E-05	32.68457
Tea intake || id:ukb-b-6066	447485	rs4410790	C	T	0.631224	0.040551	3.40E-76	0.002195	0.368776	7.62E-04	341.2682
Tea intake || id:ukb-b-6066	447485	rs17685	A	G	0.277512	0.023066	1.60E-22	0.002362	0.277512	2.13E-04	95.36355
Tea intake || id:ukb-b-6066	447485	rs141071726	A	G	0.026713	0.040732	2.20E-09	0.006812	0.026713	7.99E-05	35.75339
Tea intake || id:ukb-b-6066	447485	rs9648476	A	G	0.622954	0.012501	1.10E-08	0.002185	0.377046	7.31E-05	32.72191
Tea intake || id:ukb-b-6066	447485	rs713598	G	C	0.402254	0.013397	5.20E-10	0.002157	0.402254	8.62E-05	38.58971
Tea intake || id:ukb-b-6066	447485	rs13282783	T	C	0.285899	-0.013584	7.90E-09	0.002354	0.285899	7.44E-05	33.28919
Tea intake || id:ukb-b-6066	447485	rs56348300	G	C	0.184619	0.015882	6.10E-09	0.002732	0.184619	7.55E-05	33.79851
Tea intake || id:ukb-b-6066	447485	rs10764990	A	G	0.607155	-0.012191	1.90E-08	0.002169	0.392845	7.06E-05	31.58909
Tea intake || id:ukb-b-6066	447485	rs10752269	A	G	0.506082	-0.012873	1.30E-09	0.00212	0.493918	8.24E-05	36.87806
Tea intake || id:ukb-b-6066	447485	rs2351187	A	G	0.318935	0.012902	1.60E-08	0.002282	0.318935	7.14E-05	31.95854
Tea intake || id:ukb-b-6066	447485	rs17245213	A	G	0.208046	-0.014648	2.00E-08	0.002609	0.208046	7.04E-05	31.5207
Tea intake || id:ukb-b-6066	447485	rs10741694	C	T	0.627915	0.015004	7.90E-12	0.002194	0.372085	1.05E-04	46.78426
Tea intake || id:ukb-b-6066	447485	rs1453548	A	T	0.664929	-0.013341	3.00E-09	0.00225	0.335071	7.86E-05	35.16738
Tea intake || id:ukb-b-6066	447485	rs977474	T	C	0.833746	0.021781	2.40E-14	0.002856	0.166254	1.30E-04	58.18003

Tea intake || id:ukb-b-6066	447485	rs2783129	G	C	0.484878	-0.011733	3.80E-08	0.002133	0.484878	6.76E-05	30.25415
Tea intake || id:ukb-b-6066	447485	rs17576658	A	G	0.247081	-0.013481	4.10E-08	0.002457	0.247081	6.73E-05	30.11646
Tea intake || id:ukb-b-6066	447485	rs6829	T	C	0.596155	-0.011916	3.70E-08	0.002165	0.403845	6.77E-05	30.28172
Tea intake || id:ukb-b-6066	447485	rs2645929	G	A	0.813066	-0.014984	3.50E-08	0.002717	0.186934	6.80E-05	30.42382
Tea intake || id:ukb-b-6066	447485	rs12591786	T	C	0.158804	-0.01844	3.70E-10	0.002942	0.158804	8.78E-05	39.2738
Tea intake || id:ukb-b-6066	447485	rs2472297	T	C	0.262049	0.053345	2.30E-109	0.002401	0.262049	1.10E-03	493.6434
Tea intake || id:ukb-b-6066	447485	rs9937354	A	G	0.424074	-0.014092	4.90E-11	0.002143	0.424074	9.66E-05	43.23106
Tea intake || id:ukb-b-6066	447485	rs9302428	G	C	0.635799	0.012246	2.60E-08	0.002201	0.364201	6.92E-05	30.94841
Tea intake || id:ukb-b-6066	447485	rs2279844	A	G	0.379343	-0.011988	4.00E-08	0.002183	0.379343	6.74E-05	30.15124
Tea intake || id:ukb-b-6066	447485	rs4808193	C	T	0.335324	0.015115	1.70E-11	0.002247	0.335324	1.01E-04	45.24027
Tea intake || id:ukb-b-6066	447485	rs57631352	G	A	0.296859	-0.013104	1.70E-08	0.002321	0.296859	7.12E-05	31.86829
Tea intake || id:ukb-b-6066	447485	rs2273447	T	A	0.203788	0.017472	3.30E-11	0.002634	0.203788	9.83E-05	43.99037
Tea intake || id:ukb-b-6066	447485	rs4817505	C	T	0.38998	0.015068	4.20E-12	0.002175	0.38998	1.07E-04	48.01208
Tea intake || id:ukb-b-6066	447485	rs132904	C	G	0.778651	0.016601	7.80E-11	0.002553	0.221349	9.45E-05	42.29563
Tea intake || id:ukb-b-6066	447485	rs9624470	A	G	0.580054	0.025207	1.30E-31	0.002155	0.419946	3.06E-04	136.839
Alcohol intake frequency. || id:ukb-b-5779	462346	rs780569	A	T	0.70882	0.019803	4.00E-09	0.003365	0.29118	7.49E-05	34.64351
Alcohol intake frequency. || id:ukb-b-5779	462346	rs4503294	T	C	0.565333	0.018148	3.40E-09	0.00307	0.434667	7.55E-05	34.93181
Alcohol intake frequency. || id:ukb-b-5779	462346	rs28787109	A	G	0.40423	0.017811	7.70E-09	0.003085	0.40423	7.21E-05	33.33954
Alcohol intake frequency. || id:ukb-b-5779	462346	rs2244598	C	T	0.605114	-0.018378	3.80E-09	0.003119	0.394886	7.51E-05	34.71717
Alcohol intake frequency. || id:ukb-b-5779	462346	rs4417025	A	G	0.361153	-0.018838	2.70E-09	0.003165	0.361153	7.66E-05	35.42189
Alcohol intake frequency. || id:ukb-b-5779	462346	rs7514579	C	A	0.232457	0.019667	4.60E-08	0.003598	0.232457	6.46E-05	29.88064
Alcohol intake frequency. || id:ukb-b-5779	462346	rs2717063	A	C	0.585731	-0.02037	4.00E-11	0.003085	0.414269	9.43E-05	43.6122
Alcohol intake frequency. || id:ukb-b-5779	462346	rs6727281	T	C	0.184023	-0.024322	5.50E-10	0.00392	0.184023	8.33E-05	38.50387
Alcohol intake frequency. || id:ukb-b-5779	462346	rs780094	C	T	0.615206	-0.050994	1.30E-60	0.003105	0.384794	5.83E-04	269.7074
Alcohol intake frequency. || id:ukb-b-5779	462346	rs13390019	C	T	0.134041	0.029612	4.30E-11	0.004492	0.134041	9.40E-05	43.45875
Alcohol intake frequency. || id:ukb-b-5779	462346	rs10188314	T	C	0.470852	-0.019787	7.20E-11	0.003036	0.470852	9.19E-05	42.47524
Alcohol intake frequency. || id:ukb-b-5779	462346	rs4241258	T	C	0.13763	0.025064	1.30E-08	0.004403	0.13763	7.01E-05	32.3995
Alcohol intake frequency. || id:ukb-b-5779	462346	rs72769229	T	A	0.154942	-0.023135	3.40E-08	0.004192	0.154942	6.59E-05	30.46474
Alcohol intake frequency. || id:ukb-b-5779	462346	rs17662759	C	T	0.089115	0.030135	3.40E-08	0.00546	0.089115	6.59E-05	30.45791
Alcohol intake frequency. || id:ukb-b-5779	462346	rs1991083	T	C	0.679886	-0.022393	6.30E-12	0.003258	0.320114	1.02E-04	47.23528
Alcohol intake frequency. || id:ukb-b-5779	462346	rs473098	T	C	0.557689	-0.021741	9.10E-13	0.003043	0.442311	1.10E-04	51.02803
Alcohol intake frequency. || id:ukb-b-5779	462346	rs9829192	T	G	0.435133	0.016932	2.80E-08	0.00305	0.435133	6.66E-05	30.81374

Alcohol intake frequency. || id:ukb-b-5779	462346	rs76082653	T	C	0.054327	0.046427	3.80E-12	0.006687	0.054327	1.04E-04	48.20698
Alcohol intake frequency. || id:ukb-b-5779	462346	rs262240	T	C	0.468553	-0.017207	1.40E-08	0.003035	0.468553	6.95E-05	32.14694
Alcohol intake frequency. || id:ukb-b-5779	462346	rs9814516	T	G	0.237423	-0.025112	1.60E-12	0.003556	0.237423	1.08E-04	49.87165
Alcohol intake frequency. || id:ukb-b-5779	462346	rs7610856	A	C	0.429053	-0.023864	7.70E-15	0.00307	0.429053	1.31E-04	60.41512
Alcohol intake frequency. || id:ukb-b-5779	462346	rs1515591	G	T	0.383186	0.01823	4.90E-09	0.003116	0.383186	7.40E-05	34.22105
Alcohol intake frequency. || id:ukb-b-5779	462346	rs1228589	A	G	0.246133	0.02107	2.30E-09	0.003528	0.246133	7.71E-05	35.66573
Alcohol intake frequency. || id:ukb-b-5779	462346	rs28622224	T	C	0.280364	-0.01862	3.20E-08	0.003368	0.280364	6.61E-05	30.55903
Alcohol intake frequency. || id:ukb-b-5779	462346	rs13135092	G	A	0.083483	0.043834	1.60E-15	0.005499	0.083483	1.37E-04	63.54482
Alcohol intake frequency. || id:ukb-b-5779	462346	rs11940694	G	A	0.604193	-0.043714	1.00E-44	0.003116	0.395807	4.25E-04	196.7955
Alcohol intake frequency. || id:ukb-b-5779	462346	rs362307	T	C	0.074582	0.043305	8.40E-14	0.005802	0.074582	1.20E-04	55.70373
Alcohol intake frequency. || id:ukb-b-5779	462346	rs1229984	C	T	0.97277	-0.261708	1.40E-178	0.009185	0.02723	1.75E-03	811.8533
Alcohol intake frequency. || id:ukb-b-5779	462346	rs13102973	C	T	0.61881	-0.019407	4.90E-10	0.003119	0.38119	8.37E-05	38.72244
Alcohol intake frequency. || id:ukb-b-5779	462346	rs62339673	A	C	0.626705	0.018294	6.60E-09	0.003154	0.373295	7.28E-05	33.64262
Alcohol intake frequency. || id:ukb-b-5779	462346	rs34811474	A	G	0.230728	-0.020181	1.90E-08	0.003593	0.230728	6.82E-05	31.54661
Alcohol intake frequency. || id:ukb-b-5779	462346	rs2159935	A	G	0.490369	-0.018574	8.30E-10	0.003026	0.490369	8.15E-05	37.68185
Alcohol intake frequency. || id:ukb-b-5779	462346	rs62305780	G	C	0.102253	-0.048522	9.90E-22	0.005066	0.102253	1.98E-04	91.74105
Alcohol intake frequency. || id:ukb-b-5779	462346	rs13178443	T	C	0.276349	-0.018652	3.80E-08	0.00339	0.276349	6.55E-05	30.27433
Alcohol intake frequency. || id:ukb-b-5779	462346	rs11750777	A	G	0.209454	-0.020493	3.80E-08	0.003726	0.209454	6.54E-05	30.24776
Alcohol intake frequency. || id:ukb-b-5779	462346	rs4916723	C	A	0.420617	0.023948	1.10E-14	0.0031	0.420617	1.29E-04	59.69623
Alcohol intake frequency. || id:ukb-b-5779	462346	rs461599	C	A	0.462259	-0.019189	2.70E-10	0.00304	0.462259	8.62E-05	39.84853
Alcohol intake frequency. || id:ukb-b-5779	462346	rs56194430	T	C	0.16931	0.02254	3.10E-08	0.004071	0.16931	6.63E-05	30.64876
Alcohol intake frequency. || id:ukb-b-5779	462346	rs9403297	A	G	0.372967	0.018823	1.80E-09	0.00313	0.372967	7.82E-05	36.15788
Alcohol intake frequency. || id:ukb-b-5779	462346	rs9349379	G	A	0.405493	-0.019346	3.50E-10	0.003082	0.405493	8.52E-05	39.39585
Alcohol intake frequency. || id:ukb-b-5779	462346	rs12153855	C	T	0.10497	0.029444	2.40E-09	0.004935	0.10497	7.70E-05	35.59843
Alcohol intake frequency. || id:ukb-b-5779	462346	rs9372625	A	G	0.381706	-0.025558	2.90E-16	0.003125	0.381706	1.45E-04	66.90216
Alcohol intake frequency. || id:ukb-b-5779	462346	rs62466318	T	C	0.202827	-0.025492	1.40E-11	0.003774	0.202827	9.87E-05	45.61973
Alcohol intake frequency. || id:ukb-b-5779	462346	rs2622167	A	G	0.428653	-0.019116	4.60E-10	0.003067	0.428653	8.40E-05	38.83547
Alcohol intake frequency. || id:ukb-b-5779	462346	rs73050128	A	C	0.164488	-0.026005	2.10E-10	0.004091	0.164488	8.74E-05	40.4084
Alcohol intake frequency. || id:ukb-b-5779	462346	rs6943160	C	T	0.208646	0.020627	3.10E-08	0.003728	0.208646	6.62E-05	30.61816
Alcohol intake frequency. || id:ukb-b-5779	462346	rs4726481	T	G	0.400576	0.021761	2.30E-12	0.003102	0.400576	1.06E-04	49.21775
Alcohol intake frequency. || id:ukb-b-5779	462346	rs9648478	A	G	0.510245	0.01686	2.60E-08	0.003029	0.489755	6.70E-05	30.98532
Alcohol intake frequency. || id:ukb-b-5779	462346	rs2160935	T	C	0.604293	-0.018718	1.40E-09	0.003091	0.395707	7.93E-05	36.66665

Alcohol intake frequency. || id:ukb-b-5779	462346	rs34440851	T	C	0.157151	-0.022683	4.60E-08	0.004151	0.157151	6.46E-05	29.86657
Alcohol intake frequency. || id:ukb-b-5779	462346	rs11787216	T	C	0.369127	0.024416	2.40E-14	0.003201	0.369127	1.26E-04	58.18923
Alcohol intake frequency. || id:ukb-b-5779	462346	rs2977454	G	C	0.124072	-0.025922	1.70E-08	0.004599	0.124072	6.87E-05	31.77633
Alcohol intake frequency. || id:ukb-b-5779	462346	rs74679146	C	T	0.074515	-0.032074	2.50E-08	0.005758	0.074515	6.71E-05	31.03219
Alcohol intake frequency. || id:ukb-b-5779	462346	rs489062	A	G	0.437454	0.01665	4.90E-08	0.003053	0.437454	6.43E-05	29.74323
Alcohol intake frequency. || id:ukb-b-5779	462346	rs34473884	A	G	0.24819	-0.020362	6.20E-09	0.003503	0.24819	7.31E-05	33.77718
Alcohol intake frequency. || id:ukb-b-5779	462346	rs61873510	T	G	0.32785	0.020374	6.90E-10	0.003303	0.32785	8.23E-05	38.0445
Alcohol intake frequency. || id:ukb-b-5779	462346	rs4242715	A	G	0.680585	-0.018654	9.30E-09	0.003248	0.319415	7.13E-05	32.97927
Alcohol intake frequency. || id:ukb-b-5779	462346	rs10792669	G	A	0.505254	0.017432	9.90E-09	0.003041	0.494746	7.11E-05	32.86773
Alcohol intake frequency. || id:ukb-b-5779	462346	rs11223617	A	G	0.206155	0.025091	2.30E-11	0.003754	0.206155	9.66E-05	44.67685
Alcohol intake frequency. || id:ukb-b-5779	462346	rs550942	T	C	0.823865	0.022401	2.00E-08	0.003989	0.176135	6.82E-05	31.5384
Alcohol intake frequency. || id:ukb-b-5779	462346	rs11039429	T	C	0.454624	-0.02356	8.70E-15	0.003037	0.454624	1.30E-04	60.16374
Alcohol intake frequency. || id:ukb-b-5779	462346	rs1666658	C	T	0.392206	0.017967	6.70E-09	0.003099	0.392206	7.27E-05	33.62178
Alcohol intake frequency. || id:ukb-b-5779	462346	rs12312693	C	T	0.451772	-0.017681	6.80E-09	0.00305	0.451772	7.27E-05	33.60029
Alcohol intake frequency. || id:ukb-b-5779	462346	rs7302200	A	G	0.339998	-0.018422	8.40E-09	0.003198	0.339998	7.17E-05	33.17515
Alcohol intake frequency. || id:ukb-b-5779	462346	rs28768122	C	T	0.759525	0.0207	5.60E-09	0.003552	0.240475	7.34E-05	33.96121
Alcohol intake frequency. || id:ukb-b-5779	462346	rs7298932	G	A	0.147849	-0.023721	3.80E-08	0.004312	0.147849	6.55E-05	30.26842
Alcohol intake frequency. || id:ukb-b-5779	462346	rs58905411	A	G	0.410052	-0.026634	5.10E-18	0.003078	0.410052	1.62E-04	74.8528
Alcohol intake frequency. || id:ukb-b-5779	462346	rs1937522	G	A	0.528054	0.016898	2.50E-08	0.003032	0.471946	6.72E-05	31.05883
Alcohol intake frequency. || id:ukb-b-5779	462346	rs7330939	T	C	0.720352	-0.02133	3.70E-10	0.003405	0.279648	8.49E-05	39.25109
Alcohol intake frequency. || id:ukb-b-5779	462346	rs2535911	T	C	0.354749	-0.018848	2.70E-09	0.003168	0.354749	7.65E-05	35.38389
Alcohol intake frequency. || id:ukb-b-5779	462346	rs186347	T	G	0.463343	0.017949	4.00E-09	0.003051	0.463343	7.49E-05	34.61561
Alcohol intake frequency. || id:ukb-b-5779	462346	rs80292319	C	T	0.057704	-0.039373	1.40E-09	0.006496	0.057704	7.95E-05	36.7385
Alcohol intake frequency. || id:ukb-b-5779	462346	rs117799466	C	G	0.336989	-0.01967	3.10E-09	0.00332	0.336989	7.59E-05	35.1101
Alcohol intake frequency. || id:ukb-b-5779	462346	rs34631026	T	C	0.446061	-0.016913	2.90E-08	0.003048	0.446061	6.66E-05	30.78279
Alcohol intake frequency. || id:ukb-b-5779	462346	rs72787062	A	G	0.162767	-0.028195	6.40E-12	0.004103	0.162767	1.02E-04	47.21492
Alcohol intake frequency. || id:ukb-b-5779	462346	rs35105141	T	C	0.401541	0.026345	1.40E-17	0.003088	0.401541	1.57E-04	72.79033
Alcohol intake frequency. || id:ukb-b-5779	462346	rs1421085	C	T	0.403447	0.019939	1.00E-10	0.003085	0.403447	9.04E-05	41.77887
Alcohol intake frequency. || id:ukb-b-5779	462346	rs1104608	C	G	0.426338	0.017421	1.70E-08	0.003088	0.426338	6.88E-05	31.81719
Alcohol intake frequency. || id:ukb-b-5779	462346	rs8043563	C	G	0.737192	0.023365	1.70E-11	0.003471	0.262808	9.80E-05	45.30778
Alcohol intake frequency. || id:ukb-b-5779	462346	rs2411453	G	T	0.597353	-0.035079	7.30E-30	0.00309	0.402647	2.79E-04	128.8467
Alcohol intake frequency. || id:ukb-b-5779	462346	rs728538	G	T	0.168868	0.022875	1.80E-08	0.004063	0.168868	6.86E-05	31.70457

Alcohol intake frequency. || id:ukb-b-5779	462346	rs9906502	A	G	0.176998	0.023788	1.90E-09	0.003962	0.176998	7.80E-05	36.05323
Alcohol intake frequency. || id:ukb-b-5779	462346	rs8614	A	C	0.182509	0.024781	2.70E-10	0.003925	0.182509	8.62E-05	39.85296
Alcohol intake frequency. || id:ukb-b-5779	462346	rs4968391	T	G	0.674892	-0.01927	2.30E-09	0.003227	0.325108	7.71E-05	35.66775
Alcohol intake frequency. || id:ukb-b-5779	462346	rs9912298	C	A	0.239585	0.020589	9.70E-09	0.00359	0.239585	7.11E-05	32.89376
Alcohol intake frequency. || id:ukb-b-5779	462346	rs17690703	T	C	0.262687	0.025034	2.90E-13	0.00343	0.262687	1.15E-04	53.26282
Alcohol intake frequency. || id:ukb-b-5779	462346	rs650558	T	C	0.247918	0.020736	3.40E-09	0.003508	0.247918	7.56E-05	34.94315
Alcohol intake frequency. || id:ukb-b-5779	462346	rs1893659	A	C	0.459939	-0.029326	7.60E-22	0.003053	0.459939	2.00E-04	92.25994
Alcohol intake frequency. || id:ukb-b-5779	462346	rs5022348	T	C	0.40703	0.020264	1.40E-08	0.00357	0.40703	6.97E-05	32.21841
Alcohol intake frequency. || id:ukb-b-5779	462346	rs2043677	T	C	0.145599	0.026113	1.60E-09	0.004327	0.145599	7.88E-05	36.4173
Alcohol intake frequency. || id:ukb-b-5779	462346	rs9958320	C	T	0.153147	0.024855	5.90E-09	0.004271	0.153147	7.32E-05	33.86829
Alcohol intake frequency. || id:ukb-b-5779	462346	rs62097995	A	T	0.423591	0.020002	6.90E-11	0.003067	0.423591	9.20E-05	42.53885
Alcohol intake frequency. || id:ukb-b-5779	462346	rs2924321	A	G	0.539592	-0.019513	1.60E-10	0.00305	0.460408	8.85E-05	40.92497
Alcohol intake frequency. || id:ukb-b-5779	462346	rs4940926	C	T	0.735045	-0.0191	2.80E-08	0.003441	0.264955	6.66E-05	30.81697
Alcohol intake frequency. || id:ukb-b-5779	462346	rs838145	A	G	0.542982	0.021955	6.70E-13	0.003055	0.457018	1.12E-04	51.6292
Alcohol intake frequency. || id:ukb-b-5779	462346	rs6030200	A	G	0.31415	-0.019529	2.40E-09	0.003271	0.31415	7.71E-05	35.65032
Alcohol intake frequency. || id:ukb-b-5779	462346	rs11700855	G	A	0.093465	-0.029795	1.20E-08	0.005233	0.093465	7.01E-05	32.41878
Alcohol intake frequency. || id:ukb-b-5779	462346	rs71651683	T	C	0.0142	-0.070459	3.60E-08	0.012791	0.0142	6.56E-05	30.34506
Alcohol intake frequency. || id:ukb-b-5779	462346	rs1894544	C	G	0.454379	0.017393	1.10E-08	0.003046	0.454379	7.05E-05	32.60058
Coffee intake || id:ukb-b-5237	428860	rs516636	A	C	0.208913	0.011677	4.00E-09	0.001984	0.208913	8.07E-05	34.63153
Coffee intake || id:ukb-b-5237	428860	rs4615895	A	G	0.740926	0.012203	4.20E-11	0.00185	0.259074	1.01E-04	43.51947
Coffee intake || id:ukb-b-5237	428860	rs13387939	A	C	0.828363	0.016556	9.80E-15	0.002139	0.171637	1.40E-04	59.9293
Coffee intake || id:ukb-b-5237	428860	rs780093	C	T	0.615839	0.013294	1.00E-15	0.001657	0.384161	1.50E-04	64.3662
Coffee intake || id:ukb-b-5237	428860	rs12989746	T	G	0.249928	0.01035	2.80E-08	0.001864	0.249928	7.19E-05	30.82136
Coffee intake || id:ukb-b-5237	428860	rs1527961	C	T	0.1349	-0.013343	1.70E-08	0.002366	0.1349	7.42E-05	31.80805
Coffee intake || id:ukb-b-5237	428860	rs2597805	T	C	0.682463	0.009855	2.00E-08	0.001756	0.317537	7.34E-05	31.48837
Coffee intake || id:ukb-b-5237	428860	rs2189234	G	T	0.617795	0.009987	1.80E-09	0.001661	0.382205	8.43E-05	36.17181
Coffee intake || id:ukb-b-5237	428860	rs13163336	A	C	0.15761	0.014947	1.30E-11	0.00221	0.15761	1.07E-04	45.74182
Coffee intake || id:ukb-b-5237	428860	rs12514566	A	G	0.337107	-0.011397	2.40E-11	0.001706	0.337107	1.04E-04	44.65085
Coffee intake || id:ukb-b-5237	428860	rs2465037	A	C	0.343017	-0.010632	4.80E-10	0.001707	0.343017	9.04E-05	38.77194
Coffee intake || id:ukb-b-5237	428860	rs1338549	G	T	0.533932	-0.009451	5.60E-09	0.001622	0.466068	7.92E-05	33.96082
Coffee intake || id:ukb-b-5237	428860	rs9398171	T	C	0.71064	0.010858	1.10E-09	0.00178	0.28936	8.68E-05	37.20986
Coffee intake || id:ukb-b-5237	428860	rs73075167	T	A	0.12918	-0.016064	5.00E-11	0.002444	0.12918	1.01E-04	43.19112

Coffee intake || id:ukb-b-5237	428860	rs7811609	T	C	0.374746	0.009139	4.00E-08	0.001665	0.374746	7.03E-05	30.13697
Coffee intake || id:ukb-b-5237	428860	rs1057868	T	C	0.284986	0.019951	5.40E-29	0.001785	0.284986	2.91E-04	124.9004
Coffee intake || id:ukb-b-5237	428860	rs4410790	C	T	0.632141	0.039072	1.20E-120	0.001673	0.367859	1.27E-03	545.5062
Coffee intake || id:ukb-b-5237	428860	rs34060476	G	A	0.133855	0.018429	7.50E-15	0.00237	0.133855	1.41E-04	60.44958
Coffee intake || id:ukb-b-5237	428860	rs6469262	C	T	0.564966	-0.009153	1.90E-08	0.001629	0.435034	7.36E-05	31.57577
Coffee intake || id:ukb-b-5237	428860	rs78267637	G	C	0.038115	-0.025426	3.90E-09	0.004317	0.038115	8.09E-05	34.69517
Coffee intake || id:ukb-b-5237	428860	rs442355	C	G	0.254435	-0.011137	1.90E-09	0.001854	0.254435	8.42E-05	36.09544
Coffee intake || id:ukb-b-5237	428860	rs10119174	C	G	0.571035	-0.009398	1.00E-08	0.001642	0.428965	7.64E-05	32.77378
Coffee intake || id:ukb-b-5237	428860	rs117810762	A	G	0.017881	0.035909	6.20E-09	0.006179	0.017881	7.88E-05	33.77531
Coffee intake || id:ukb-b-5237	428860	rs61928609	C	A	0.835328	-0.014731	1.30E-11	0.002175	0.164672	1.07E-04	45.85331
Coffee intake || id:ukb-b-5237	428860	rs2472297	T	C	0.262883	0.046471	1.10E-142	0.001827	0.262883	1.51E-03	646.7319
Coffee intake || id:ukb-b-5237	428860	rs117968677	A	G	0.024207	-0.03103	1.90E-08	0.005516	0.024207	7.38E-05	31.64526
Coffee intake || id:ukb-b-5237	428860	rs8056750	T	C	0.359129	0.010533	1.30E-09	0.001737	0.359129	8.57E-05	36.77623
Coffee intake || id:ukb-b-5237	428860	rs1421085	C	T	0.40357	0.018543	1.70E-29	0.001644	0.40357	2.96E-04	127.1585
Coffee intake || id:ukb-b-5237	428860	rs62064918	T	C	0.244545	-0.010308	4.10E-08	0.001879	0.244545	7.02E-05	30.10097
Coffee intake || id:ukb-b-5237	428860	rs57918684	A	G	0.154747	0.012886	8.60E-09	0.002238	0.154747	7.73E-05	33.14106
Coffee intake || id:ukb-b-5237	428860	rs7224815	T	A	0.407832	-0.01086	3.70E-11	0.001642	0.407832	1.02E-04	43.76514
Coffee intake || id:ukb-b-5237	428860	rs630194	C	T	0.343374	-0.011353	2.30E-11	0.001699	0.343374	1.04E-04	44.67821
Coffee intake || id:ukb-b-5237	428860	rs1942965	C	T	0.504585	-0.008903	3.80E-08	0.001619	0.495415	7.05E-05	30.23597
Coffee intake || id:ukb-b-5237	428860	rs476828	C	T	0.237409	0.017346	5.60E-20	0.001895	0.237409	1.95E-04	83.75026
Coffee intake || id:ukb-b-5237	428860	rs56113850	C	T	0.578109	0.012667	8.90E-15	0.001634	0.421891	1.40E-04	60.12864
Coffee intake || id:ukb-b-5237	428860	rs75347775	A	G	0.244531	0.01045	2.70E-08	0.001879	0.244531	7.21E-05	30.93282
Coffee intake || id:ukb-b-5237	428860	rs6063085	C	A	0.373473	0.010411	4.50E-10	0.001669	0.373473	9.07E-05	38.89896
Coffee intake || id:ukb-b-5237	428860	rs6062682	T	C	0.464546	0.01037	2.50E-10	0.001639	0.464546	9.33E-05	40.02003
Coffee intake || id:ukb-b-5237	428860	rs13054099	C	T	0.261004	-0.010778	4.30E-09	0.001836	0.261004	8.03E-05	34.4603
Coffee intake || id:ukb-b-5237	428860	rs17842490	G	A	0.014248	-0.045168	3.30E-11	0.006808	0.014248	1.03E-04	44.01139
Water intake || id:ukb-b-14898	427588	rs34967813	G	A	0.311025	-0.012055	7.10E-10	0.001956	0.311025	8.89E-05	38.00285
Water intake || id:ukb-b-14898	427588	rs2305813	C	G	0.123354	-0.019115	5.00E-12	0.002769	0.123354	1.11E-04	47.66929
Water intake || id:ukb-b-14898	427588	rs782221	T	C	0.200429	-0.01271	1.90E-08	0.002262	0.200429	7.39E-05	31.58082
Water intake || id:ukb-b-14898	427588	rs182050989	T	C	0.02835	0.030528	2.40E-08	0.005474	0.02835	7.27E-05	31.10302
Water intake || id:ukb-b-14898	427588	rs11890994	T	A	0.371683	0.011095	3.60E-09	0.001879	0.371683	8.15E-05	34.85051
Water intake || id:ukb-b-14898	427588	rs2198234	T	G	0.528757	0.01167	1.30E-10	0.001816	0.471243	9.66E-05	41.30014

Water intake || id:ukb-b-14898	427588	rs11125629	G	A	0.546761	0.010867	2.50E-09	0.001823	0.453239	8.31E-05	35.53855
Water intake || id:ukb-b-14898	427588	rs9830293	G	A	0.073462	0.019082	4.00E-08	0.003475	0.073462	7.05E-05	30.15816
Water intake || id:ukb-b-14898	427588	rs7626335	C	A	0.668446	0.010769	2.50E-08	0.001933	0.331554	7.26E-05	31.04708
Water intake || id:ukb-b-14898	427588	rs6844845	G	A	0.057487	-0.021514	3.30E-08	0.003894	0.057487	7.14E-05	30.52292
Water intake || id:ukb-b-14898	427588	rs6835325	G	T	0.30096	-0.011865	2.00E-09	0.001978	0.30096	8.41E-05	35.97606
Water intake || id:ukb-b-14898	427588	rs67174962	A	G	0.232324	-0.012265	1.30E-08	0.002157	0.232324	7.56E-05	32.34077
Water intake || id:ukb-b-14898	427588	rs6905712	A	T	0.664977	0.011088	8.90E-09	0.001928	0.335023	7.73E-05	33.067
Water intake || id:ukb-b-14898	427588	rs2274156	T	C	0.258771	-0.011485	2.90E-08	0.002071	0.258771	7.19E-05	30.76605
Water intake || id:ukb-b-14898	427588	rs6957745	C	T	0.202686	-0.015292	1.40E-11	0.002262	0.202686	1.07E-04	45.69578
Water intake || id:ukb-b-14898	427588	rs3808058	T	C	0.117807	0.018332	7.50E-11	0.002816	0.117807	9.91E-05	42.37326
Water intake || id:ukb-b-14898	427588	rs4410790	C	T	0.629877	-0.030871	8.60E-61	0.001877	0.370123	6.32E-04	270.5409
Water intake || id:ukb-b-14898	427588	rs10954732	A	G	0.671297	0.01062	3.60E-08	0.001928	0.328703	7.09E-05	30.33761
Water intake || id:ukb-b-14898	427588	rs2656285	C	T	0.709918	0.012242	1.10E-09	0.002007	0.290082	8.70E-05	37.19657
Water intake || id:ukb-b-14898	427588	rs9414686	T	C	0.178007	0.01476	8.60E-10	0.002406	0.178007	8.80E-05	37.62187
Water intake || id:ukb-b-14898	427588	rs10758255	A	T	0.609855	-0.010787	6.70E-09	0.00186	0.390145	7.86E-05	33.61724
Water intake || id:ukb-b-14898	427588	rs11140831	G	A	0.514151	-0.012382	1.50E-11	0.001836	0.485849	1.06E-04	45.47697
Water intake || id:ukb-b-14898	427588	rs35028442	C	G	0.137272	0.016424	9.90E-10	0.002688	0.137272	8.73E-05	37.33586
Water intake || id:ukb-b-14898	427588	rs11012726	C	T	0.307334	-0.012241	6.20E-10	0.001979	0.307334	8.94E-05	38.24397
Water intake || id:ukb-b-14898	427588	rs3763874	A	G	0.41763	0.01633	6.90E-19	0.00184	0.41763	1.84E-04	78.79405
Water intake || id:ukb-b-14898	427588	rs7124005	T	C	0.415348	0.010367	2.50E-08	0.00186	0.415348	7.26E-05	31.04981
Water intake || id:ukb-b-14898	427588	rs2229357	A	G	0.240393	-0.012206	7.90E-09	0.002115	0.240393	7.79E-05	33.30299
Water intake || id:ukb-b-14898	427588	rs34940743	G	A	0.346783	-0.010392	4.40E-08	0.001898	0.346783	7.01E-05	29.96471
Water intake || id:ukb-b-14898	427588	rs146394874	A	C	0.049323	0.026142	5.80E-10	0.00422	0.049323	8.97E-05	38.37909
Water intake || id:ukb-b-14898	427588	rs1963510	G	A	0.458107	0.013307	2.60E-13	0.00182	0.458107	1.25E-04	53.48612
Water intake || id:ukb-b-14898	427588	rs4603502	C	T	0.292378	-0.013072	5.40E-11	0.001993	0.292378	1.01E-04	43.02579
Water intake || id:ukb-b-14898	427588	rs2472297	T	C	0.26061	-0.033419	2.40E-59	0.002057	0.26061	6.17E-04	263.9061
Water intake || id:ukb-b-14898	427588	rs8054636	C	T	0.537476	-0.010331	2.40E-08	0.001851	0.462524	7.28E-05	31.14794
Water intake || id:ukb-b-14898	427588	rs2289292	T	C	0.334337	-0.012769	3.40E-11	0.001927	0.334337	1.03E-04	43.90847
Water intake || id:ukb-b-14898	427588	rs1421085	C	T	0.403711	0.01201	7.70E-11	0.001846	0.403711	9.90E-05	42.33464
Water intake || id:ukb-b-14898	427588	rs2435200	A	G	0.412694	0.011214	1.10E-09	0.001838	0.412694	8.70E-05	37.20757
Water intake || id:ukb-b-14898	427588	rs4239466	C	A	0.391897	0.010643	2.00E-08	0.001896	0.391897	7.36E-05	31.49242
Water intake || id:ukb-b-14898	427588	rs9957088	T	C	0.253192	-0.01183	1.40E-08	0.002087	0.253192	7.51E-05	32.12378

Water intake || id:ukb-b-14898	427588	rs429358	C	T	0.154368	0.016681	3.00E-11	0.002509	0.154368	1.03E-04	44.20171
Water intake || id:ukb-b-14898	427588	rs56100328	G	A	0.090196	-0.017554	4.80E-08	0.003217	0.090196	6.96E-05	29.78116
Water intake || id:ukb-b-14898	427588	rs3746410	G	A	0.20396	0.016839	7.30E-14	0.00225	0.20396	1.31E-04	55.99821
Hot drink temperature || id:ukb-b-14203	457873	rs79564740	C	T	0.030417	-0.019251	2.50E-08	0.003453	0.030417	6.79E-05	31.08344
Hot drink temperature || id:ukb-b-14203	457873	rs2786529	G	C	0.209494	-0.008307	1.20E-08	0.001456	0.209494	7.11E-05	32.55118
Hot drink temperature || id:ukb-b-14203	457873	rs12132579	T	C	0.357498	0.007053	1.20E-08	0.001238	0.357498	7.08E-05	32.43571
Hot drink temperature || id:ukb-b-14203	457873	rs10927006	C	T	0.143611	-0.01374	4.80E-16	0.001693	0.143611	1.44E-04	65.88018
Hot drink temperature || id:ukb-b-14203	457873	rs12038134	A	T	0.508213	0.006537	3.60E-08	0.001187	0.491787	6.63E-05	30.35141
Hot drink temperature || id:ukb-b-14203	457873	rs1001880	C	T	0.313764	0.007384	8.90E-09	0.001284	0.313764	7.22E-05	33.06724
Hot drink temperature || id:ukb-b-14203	457873	rs12622811	T	C	0.298571	-0.010264	3.10E-15	0.001302	0.298571	1.36E-04	62.17966
Hot drink temperature || id:ukb-b-14203	457873	rs74384251	T	C	0.136886	0.009525	3.50E-08	0.001727	0.136886	6.65E-05	30.43148
Hot drink temperature || id:ukb-b-14203	457873	rs62134736	A	G	0.2591	0.007577	2.00E-08	0.001351	0.2591	6.87E-05	31.45225
Hot drink temperature || id:ukb-b-14203	457873	rs17409597	C	T	0.491526	-0.006986	4.90E-09	0.001194	0.491526	7.47E-05	34.21904
Hot drink temperature || id:ukb-b-14203	457873	rs1260326	C	T	0.604332	-0.009185	3.20E-14	0.00121	0.395668	1.26E-04	57.59346
Hot drink temperature || id:ukb-b-14203	457873	rs1568452	T	C	0.382005	-0.012052	5.90E-23	0.001222	0.382005	2.13E-04	97.32773
Hot drink temperature || id:ukb-b-14203	457873	rs17024335	C	T	0.223585	-0.008289	6.90E-09	0.001431	0.223585	7.33E-05	33.56662
Hot drink temperature || id:ukb-b-14203	457873	rs6736362	T	C	0.560656	-0.006648	2.70E-08	0.001195	0.439344	6.76E-05	30.93412
Hot drink temperature || id:ukb-b-14203	457873	rs1997468	T	C	0.439876	0.010862	9.40E-20	0.001194	0.439876	1.81E-04	82.72858
Hot drink temperature || id:ukb-b-14203	457873	rs34292254	T	G	0.552492	0.007443	4.70E-10	0.001195	0.447508	8.48E-05	38.81734
Hot drink temperature || id:ukb-b-14203	457873	rs1144428	A	G	0.790277	0.012221	5.80E-17	0.00146	0.209723	1.53E-04	70.02952
Hot drink temperature || id:ukb-b-14203	457873	rs9835772	T	A	0.243662	-0.00808	4.90E-09	0.001381	0.243662	7.48E-05	34.2449
Hot drink temperature || id:ukb-b-14203	457873	rs12695358	T	G	0.597342	-0.007062	5.40E-09	0.00121	0.402658	7.43E-05	34.04412
Hot drink temperature || id:ukb-b-14203	457873	rs888405	G	A	0.794793	0.009196	3.80E-10	0.001468	0.205207	8.56E-05	39.21997
Hot drink temperature || id:ukb-b-14203	457873	rs17023019	G	A	0.647408	0.012121	1.70E-22	0.001242	0.352592	2.08E-04	95.24572
Hot drink temperature || id:ukb-b-14203	457873	rs11710570	C	T	0.448108	-0.007679	1.20E-10	0.001192	0.448108	9.07E-05	41.5352
Hot drink temperature || id:ukb-b-14203	457873	rs1513475	C	T	0.34937	-0.008914	7.80E-13	0.001244	0.34937	1.12E-04	51.34228
Hot drink temperature || id:ukb-b-14203	457873	rs2255015	A	G	0.454555	-0.006978	4.70E-09	0.001192	0.454555	7.49E-05	34.2907
Hot drink temperature || id:ukb-b-14203	457873	rs2702576	G	A	0.621311	0.007416	1.40E-09	0.001224	0.378689	8.02E-05	36.73444
Hot drink temperature || id:ukb-b-14203	457873	rs2952894	C	T	0.664339	0.0072	1.00E-08	0.001258	0.335661	7.16E-05	32.76675
Hot drink temperature || id:ukb-b-14203	457873	rs6876382	G	C	0.05895	-0.015142	2.00E-09	0.002524	0.05895	7.86E-05	35.9776
Hot drink temperature || id:ukb-b-14203	457873	rs111251222	G	A	0.258804	-0.007733	1.30E-08	0.00136	0.258804	7.06E-05	32.34034
Hot drink temperature || id:ukb-b-14203	457873	rs2728784	G	A	0.208554	-0.011009	5.30E-14	0.001463	0.208554	1.24E-04	56.63313

Hot drink temperature || id:ukb-b-14203	457873	rs6892119	G	A	0.574513	-0.008065	3.90E-11	0.00122	0.425487	9.54E-05	43.68664
Hot drink temperature || id:ukb-b-14203	457873	rs6469	T	C	0.127478	0.010933	1.50E-08	0.001931	0.127478	7.00E-05	32.05448
Hot drink temperature || id:ukb-b-14203	457873	rs1326609	A	G	0.371579	-0.007807	2.10E-10	0.001228	0.371579	8.82E-05	40.39039
Hot drink temperature || id:ukb-b-14203	457873	rs12664031	A	C	0.289569	-0.010451	3.00E-14	0.001376	0.289569	1.26E-04	57.71393
Hot drink temperature || id:ukb-b-14203	457873	rs3132487	T	G	0.345911	-0.009646	4.80E-14	0.00128	0.345911	1.24E-04	56.7944
Hot drink temperature || id:ukb-b-14203	457873	rs9372734	T	C	0.482495	-0.008024	1.40E-11	0.001188	0.482495	9.96E-05	45.60264
Hot drink temperature || id:ukb-b-14203	457873	rs62403110	A	G	0.302512	-0.007432	8.40E-09	0.00129	0.302512	7.25E-05	33.18854
Hot drink temperature || id:ukb-b-14203	457873	rs3117103	T	A	0.139554	-0.01526	4.00E-19	0.001707	0.139554	1.74E-04	79.89228
Hot drink temperature || id:ukb-b-14203	457873	rs58391518	C	T	0.291369	-0.007371	1.70E-08	0.001307	0.291369	6.94E-05	31.79813
Hot drink temperature || id:ukb-b-14203	457873	rs4410790	C	T	0.630852	-0.010958	4.80E-19	0.001229	0.369148	1.74E-04	79.50672
Hot drink temperature || id:ukb-b-14203	457873	rs2113336	A	C	0.470426	-0.00721	1.40E-09	0.001192	0.470426	7.99E-05	36.60524
Hot drink temperature || id:ukb-b-14203	457873	rs35968894	G	A	0.377461	-0.007216	3.80E-09	0.001225	0.377461	7.58E-05	34.71938
Hot drink temperature || id:ukb-b-14203	457873	rs210600	A	G	0.27676	0.007675	7.10E-09	0.001326	0.27676	7.32E-05	33.50244
Hot drink temperature || id:ukb-b-14203	457873	rs2360802	T	A	0.225125	-0.009424	3.60E-11	0.001423	0.225125	9.57E-05	43.83566
Hot drink temperature || id:ukb-b-14203	457873	rs62572325	A	C	0.13467	0.011211	1.20E-10	0.001739	0.13467	9.07E-05	41.53897
Hot drink temperature || id:ukb-b-14203	457873	rs62580693	C	G	0.252484	0.008041	4.10E-09	0.001367	0.252484	7.55E-05	34.58171
Hot drink temperature || id:ukb-b-14203	457873	rs1027583	C	G	0.272924	-0.007661	1.20E-08	0.001344	0.272924	7.10E-05	32.50451
Hot drink temperature || id:ukb-b-14203	457873	rs10822089	G	A	0.523143	-0.007278	8.90E-10	0.001188	0.476857	8.20E-05	37.55893
Hot drink temperature || id:ukb-b-14203	457873	rs10764990	A	G	0.607319	0.009482	5.80E-15	0.001214	0.392681	1.33E-04	60.96154
Hot drink temperature || id:ukb-b-14203	457873	rs10829603	G	T	0.451828	0.007357	7.50E-10	0.001195	0.451828	8.27E-05	37.8797
Hot drink temperature || id:ukb-b-14203	457873	rs17461712	C	T	0.167426	0.012089	3.40E-14	0.001595	0.167426	1.25E-04	57.46273
Hot drink temperature || id:ukb-b-14203	457873	rs11570094	A	C	0.295801	0.007546	6.80E-09	0.001302	0.295801	7.34E-05	33.59028
Hot drink temperature || id:ukb-b-14203	457873	rs4492837	C	G	0.586887	-0.006792	1.80E-08	0.001206	0.413113	6.93E-05	31.71238
Hot drink temperature || id:ukb-b-14203	457873	rs1447182	C	T	0.782248	0.009392	9.50E-11	0.001451	0.217752	9.15E-05	41.91924
Hot drink temperature || id:ukb-b-14203	457873	rs61909866	T	C	0.316065	-0.00734	8.90E-09	0.001277	0.316065	7.22E-05	33.06389
Hot drink temperature || id:ukb-b-14203	457873	rs2712661	A	G	0.769898	-0.008238	5.10E-09	0.001409	0.230102	7.46E-05	34.16206
Hot drink temperature || id:ukb-b-14203	457873	rs10744560	T	C	0.340057	-0.00689	3.90E-08	0.001254	0.340057	6.59E-05	30.19406
Hot drink temperature || id:ukb-b-14203	457873	rs826848	T	C	0.933713	-0.014781	6.10E-10	0.002388	0.066287	8.36E-05	38.30225
Hot drink temperature || id:ukb-b-14203	457873	rs4477562	T	C	0.1287	-0.01047	4.50E-09	0.001786	0.1287	7.51E-05	34.38642
Hot drink temperature || id:ukb-b-14203	457873	rs9570736	G	A	0.518982	-0.006557	3.30E-08	0.001187	0.481018	6.66E-05	30.49611
Hot drink temperature || id:ukb-b-14203	457873	rs34759521	A	T	0.227087	-0.008259	5.60E-09	0.001417	0.227087	7.42E-05	33.97192
Hot drink temperature || id:ukb-b-14203	457873	rs2472297	T	C	0.261771	-0.015385	2.60E-30	0.001345	0.261771	2.86E-04	130.9122

Hot drink temperature || id:ukb-b-14203	457873	rs34935263	G	A	0.533192	-0.006817	1.00E-08	0.00119	0.466808	7.17E-05	32.82304
Hot drink temperature || id:ukb-b-14203	457873	rs58726064	G	C	0.442086	0.006771	1.50E-08	0.001196	0.442086	7.00E-05	32.06313
Hot drink temperature || id:ukb-b-14203	457873	rs55880962	A	G	0.192325	0.008487	1.70E-08	0.001505	0.192325	6.94E-05	31.78738
Hot drink temperature || id:ukb-b-14203	457873	rs17513240	T	A	0.172466	-0.010646	1.20E-11	0.001569	0.172466	1.01E-04	46.04575
Hot drink temperature || id:ukb-b-14203	457873	rs4452038	T	C	0.389867	-0.011175	6.50E-20	0.001223	0.389867	1.82E-04	83.45575
Hot drink temperature || id:ukb-b-14203	457873	rs1132845	T	C	0.404377	-0.006993	8.20E-09	0.001213	0.404377	7.26E-05	33.23491
Hot drink temperature || id:ukb-b-14203	457873	rs1680349	A	G	0.563781	-0.00673	1.90E-08	0.001198	0.436219	6.89E-05	31.57129
Hot drink temperature || id:ukb-b-14203	457873	rs6045331	G	C	0.37918	-0.007591	6.10E-10	0.001227	0.37918	8.36E-05	38.2822
Hot drink temperature || id:ukb-b-14203	457873	rs2825972	G	A	0.292758	0.008751	1.90E-11	0.001304	0.292758	9.84E-05	45.04115
Hot drink temperature || id:ukb-b-14203	457873	rs2032512	C	A	0.607796	-0.008918	2.10E-13	0.001215	0.392204	1.18E-04	53.89186

Supplementary Table 3. Detailed information of exposures dataset.

GWAS ID	Food types	Trait	Sample Size	SNPs	Consortium	Population	Category
ukb-b-2862	Meat and poultry	Beef intake	461053	9851867	MRC-IEU	European	Categorical (single)
ukb-b-14179	Meat and poultry	Lamb/mutton intake	460006	9851867	MRC-IEU	European	Categorical (single)
ukb-b-5640	Meat and poultry	Pork intake	460162	9851867	MRC-IEU	European	Categorical (single)
ukb-b-8006	Meat and poultry	Poultry intake	461900	9851867	MRC-IEU	European	Categorical (single)
ukb-b-6324	Meat and poultry	Processed meat intake	461981	9851867	MRC-IEU	European	Categorical (single)
ukb-b-17627	Seafood	Non-oily fish intake	460880	9851867	MRC-IEU	European	Categorical (single)
ukb-b-2209	Seafood	Oily fish intake	460443	9851867	MRC-IEU	European	Categorical (single)
ukb-b-8089	Vegetable	Cooked vegetable intake	448651	9851867	MRC-IEU	European	Integer, tablespoons/week
ukb-b-1996	Vegetable	Salad/raw vegetable intake	435435	9851867	MRC-IEU	European	Integer tablespoons/day
ukb-b-16576	Fruit	Dried fruit intake	421764	9851867	MRC-IEU	European	Integer, pieces/week
ukb-b-3881	Fruit	Fresh fruit intake	446462	9851867	MRC-IEU	European	Categorical (single)
ukb-b-11348	Bread	Bread intake	452236	9851867	MRC-IEU	European	Integer, slices/week
ukb-b-15926	Grains, nuts, and seeds	Cereal intake	441640	9851867	MRC-IEU	European	Integer, bowls/week
ukb-b-1489	Dairy products	Cheese intake	451486	9851867	MRC-IEU	European	Categorical (single)
ukb-b-8121	Food additive	Salt added to food	462630	9851867	MRC-IEU	European	Categorical (single)
ukb-b-6066	Drinks	Tea intake	447485	9851867	MRC-IEU	European	Integer, cups/day
ukb-b-5779	Drinks	Alcohol intake frequency	462346	9851867	MRC-IEU	European	Categorical (single)
ukb-b-5237	Drinks	Coffee intake	428860	9851867	MRC-IEU	European	Integer, cups/week

ukb-b-14898	Drinks	Water intake	427588	9851867	MRC-IEU	European	Integer glasses/day
ukb-b-14203	/	Hot drink temperature	457873	9851867	MRC-IEU	European	Categorical (single)

Supplementary Table 4. Detailed information of intermediators and outcomes dataset.

Category	GWAS ID	Trait	Sample size	Case	Control	SNPs	Consortium	Population
Intermediates	ieu-a-72	Waist-to-hip ratio (WHR)	224459	/	/	2562516	GIANT	Mixed
	ukb-b-2303	Body mass index (BMI)	454884	/	/	9851867	MRC-IEU	European
	ebi-a-GCST90025954	Low density lipoprotein cholesterol levels (LDL-C)	389189	/	/	10783697	NA	European
	ebi-a-GCST90014007	High density lipoprotein cholesterol levels (HDL-C)	357810	/	/	10783660	NA	European
Outcomes	ebi-a-GCST90038613	Stroke	484598	6925	477673	9587836	NA	NA
	ebi-a-GCST005843	Ischemic stroke	440328	34217	406111	7537579	NA	European
	ebi-a-GCST006910	Ischemic stroke (cardioembolic)	211763	7193	406111	8271294	NA	European
	ebi-a-GCST005841	Ischemic stroke (small-vessel)	198048	5386	192662	6150261	NA	European
	ebi-a-GCST005840	Ischemic stroke (large artery atherosclerosis)	150765	4373	406111	7992739	NA	European

Detailed information of intermediators and outcomes dataset. Two adiposity indicators (waist-to-hip ratio [WHR] and body mass index [BMI]), and two lipoprotein cholesterol indicators (Low Density Lipoprotein Cholesterol [LDL-C]; High Density Lipoprotein Cholesterol [HDL-C]) were selected as potential intermediates.

Supplementary Table 5. Detailed information on the significant causal relationship between dietary habits and stroke.

Exposures	Outcomes	Methods	SNPs	P Value	Beta(95%CI)	Cochran's Q test		MR Egger	
						Q Value	P Value	Intercept	P Value
Dried fruit intake	Stroke	MR Egger	41	0.320	-0.017(-0.051/0.016)				
		Weighted median	41	0.173	-0.007(-0.017/0.030)				
		IVW	41	**0.013**	-0.009(-0.017/-0.002)	47.63	0.19	9.90e-05	0.641
		Weighted mode	41	0.959	0.0006(-0.023/0.024)				
Oily fish intake	Stroke	MR Egger	61	0.542	-0.007(-0.028/0.015)				
		Weighted median	61	0.573	-0.002(-0.009/0.005)				
		IVW	61	**0.020**	-0.006(-0.011/-0.0009)	70.89	0.159	7.80e-06	0.961

		Weighted mode	61	0.956	0.0004(-0.015/0.016)				
Dried fruit intake	Ischemic stroke	MR Egger	40	0.684	0.306(-1.157/1.770)				
		Weighted median	40	0.077	-0.352(-0.733/0.028)				
		IVW	40	**0.003**	-0.475(-0.792/-0.158)	63.32	0.008	-1.00e-02	0.29
		Weighted mode	40	0.402	-0.330(-1.057/0.397)				
Cheese intake	Ischemic stroke	MR Egger	61	0.679	0.159(-0.590/0.908)				
		Weighted median	61	0.007	-0.341(-0.579/-0.102)				
		IVW	61	**0.003**	-0.275(-0.457/-0.092)	79.91	0.044	-8.00e-03	0.247
		Weighted mode	61	0.183	-0.409(-0.996/0.178)				
Lamb/mutton intake	Cardioembolic	MR Egger	31	0.192	2.273(-1.062/9.704)				
		Weighted median	31	0.154	0.768(-0.287/2.155)				
		IVW	31	**0.048**	0.805(0.009/2.237)	33.82	0.288	-1.60e-02	0.382
		Weighted mode	31	0.385	0.836(-1.022/2.306)				
Dried fruit intake	Small vessel	MR Egger	40	0.784	-0.421(-3.416/2.574)				
		Weighted median	40	0.452	-0.300(-1.115/0.514)				
		IVW	40	**0.033**	-0.682(-1.308/-0.055)	60.07	0.017	-3.00e-03	0.862
		Weighted mode	40	0.797	0.250(-1.432/1.932)				
Poultry intake	Small vessel	MR Egger	7	0.429	16.73(-21.42/54.88)				
		Weighted median	7	0.259	1.091(-0.728/2.909)				
		IVW	7	**0.044**	1.349(0.039/2.660)	6.148	0.407	-1.67e-01	0.465
		Weighted mode	7	0.718	0.539(-2.221/3.299)				
Cereal intake	Large artery	MR Egger	37	0.829	0.283(-2.266/2.832)				
		Weighted median	37	0.094	-0.703(-1.550/0.144)				
		IVW	37	**0.010**	-0.756(-1.332/-0.181)	34.79	0.526	-1.40e-02	0.418
		Weighted mode	37	0.080	-1.799(-3.727/0.128)				
Salt added to food	Large artery	MR Egger	101	0.489	0.489(-0.891/1.869)				
		Weighted median	101	0.017	0.596(0.099/1.906)				
		IVW	101	**0.033**	0.432(0.036/0.829)	142.5	0.0034	-8.00e-04	0.933
		Weighted mode	101	0.225	0.752(-0.488/1.993)				
Hot drink temperature	Large artery	MR Egger	66	0.460	-1.094(-3.979/1.791)				
		Weighted median	66	0.026	-1.048(-2.021/-0.074)				

IVW	66	**0.0098**	-0.862(-1.516/-0.207)	68.54	0.358	2.00e-03	0.872
Weighted mode	66	0.148	-1.827(-4.418/0.495)				

Supplementary Table 6. Detailed information on the significant causal relationship between dietary habits and obesity.

Exposures	Intermediates	Methods	SNPs	P value	Beta(95%CI)	Cochran's Q test		MR-Egger	
						Q Value	P Value	Intercept	P Value
Dried fruit intake	BMI	MR Egger	41	8.30e-02	-1.244(-2.615/0.127)				
		Weighted median	41	8.30e-13	-0.464(-0.592/-0.337)				
		IVW	41	**1.20e-02**	-0.395(-0.702/-0.088)	1195.6	1.20e-224	0.011	0.221
		Weighted mode	41	4.10e-03	-0.474(-0.779/-0.169)				
	WHR	MR Egger	31	0.934	0.064(-1.441/1.569)				
		Weighted median	31	0.003	-0.354(-0.584/-0.124)				
		IVW	31	0.085	-0.235(-0.503/0.032)	112.1	2.10e-11	-0.004	0.695
		Weighted mode	31	0.087	-0.366(-0.773/0.040)				
Oily fish intake	BMI	MR Egger	61	0.994	-0.004(-0.936/0.929)				
		Weighted median	61	0.148	-0.067(-0.158/0.024)				
		IVW	61	0.724	0.041(-0.186/0.268)	2038	0	0.0007	0.923
		Weighted mode	61	0.970	0.003(-0.167/0.174)				
	WHR	MR Egger	47	0.983	-0.011(-0.989/0.968)				
		Weighted median	47	0.241	-0.090(-0.246/0.062)				
		IVW	47	0.447	0.085(-0.135/0.306)	265.1	1.50e-32	0.001	0.844
		Weighted mode	47	0.596	-0.079(-0.373/0.213)				
Cheese intake	BMI	MR Egger	64	7.88e-01	0.082(-0.513/0.677)				
		Weighted median	64	5.10e-05	-0.168(-0.249/-0.087)				
		IVW	64	**2.30e-09**	-0.440(-0.584/-0.296)	1075.2	4.60e-1984	-0.009	0.081
		Weighted mode	64	3.70e-02	-0.107(-0.205/-0.009)				
	WHR	MR Egger	43	4.66e-01	0.207(-0.345/0.759)				
		Weighted median	43	1.69e-06	-0.326(-0.460/-0.193)				

		IVW	43	**2.94e-10**	-0.377(-0.494/-0.259)	77.54	7.00e-04	-0.009	0.509
		Weighted mode	43	3.92e-02	-0.303(-0.582/-0.024)				
Cereal intake	BMI	MR Egger	39	5.60e-02	-0.713(-1.422/-0.005)				
		Weighted median	39	1.90e-08	-0.317(-0.427/-0.206)				
		IVW	39	**2.90e-07**	-0.425(-0.587/-0.262)	374.5	6.50e-57	0.004	0.417
		Weighted mode	39	4.18e-03	-0.319(-0.523/-0.114)				
	WHR	MR Egger	33	1.08e-01	-0.590(-1.287/0.108)				
		Weighted median	33	3.87e-05	-0.359(-0.530/-0.188)				
		IVW	33	**2.73e-05**	-0.330(-0.484/-0.176)	59.68	2.00e-03	0.004	0.46
		Weighted mode	33	1.99e-02	-0.396(-0.712/-0.079)				
Hot drink temperature	BMI	MR Egger	69	0.808	-0.124(-1.117/0.870)				
		Weighted median	69	0.534	0.050(-0.107/0.206)				
		IVW	69	0.881	0.017(-0.211/0.246)	1018	2.90e-169	0.001	0.776
		Weighted mode	69	0.144	0.467(-0.153/1.088)				
	WHR	MR Egger	54	0.778	0.109(-0.641/0.859)				
		Weighted median	54	0.339	-0.108(-0.329/0.114)				
		IVW	54	0.475	0.065(-0.113/0.242)	91.32	0.0008	-0.0004	0.906
		Weighted mode	54	0.233	-0.292(-0.765/0.182)				
Lamb/mutton intake	BMI	MR Egger	31	0.809	0.224(-1.579/2.028)				
		Weighted median	31	0.897	0.013(-0.179/0.205)				
		IVW	31	0.520	-0.144(-0.583/0.295)	1001	3.40e-191	-0.004	0.683
		Weighted mode	31	0.405	-0.133(-0.442/0.176)				
	WHR	MR Egger	22	0.841	0.133(-1.142/1.407)				
		Weighted median	22	0.598	-0.076(-0.358/0.206)				
		IVW	22	0.160	-0.198(-0.475/0.078)	44.53	0.002	-0.004	0.608
		Weighted mode	22	0.731	0.080(-0.368/0.527)				
Poultry intake	BMI	MR Egger	7	4.17e-01	7.223(-8.796/23.24)				

		Weighted median	7	2.00e-03	0.458(0.163/0.754)				
		IVW	7	**3.40e-02**	0.572(0.042/1.101)	81.14	2.10e-15	-0.072	0.453
		Weighted mode	7	2.51e-01	0.395(-0.214/1.004)				
	WHR	MR Egger	7	0.309	7.648(-5.606/20.90)				
		Weighted median	7	0.505	0.138(-0.267/0.543)				
		IVW	7	0.060	0.429(-0.017/0.875)	15.27	0.018	-0.078	0.334
		Weighted mode	7	0.741	0.083(-0.387/0.553)				
Salt added to food	BMI	MR Egger	102	4.07e-01	-0.138(-0.462/0.187)				
		Weighted median	102	5.71e-01	0.019(-0.048/0.086)				
		IVW	102	**2.00e-03**	0.162(0.062/0.262)	1068	3.00e-161	0.005	0.06
		Weighted mode	102	4.95e-01	-0.031(-0.120/0.058)				
	WHR	MR Egger	80	7.71e-01	-0.047(-0.360/0.267)				
		Weighted median	80	3.80e-02	0.114(0.006/0.222)				
		IVW	80	**2.00e-04**	0.175(0.082/0.268)	129.8	3.00e-04	0.03	0.151
		Weighted mode	80	3.69e-01	0.098(-0.114/0.310)				

Supplementary Table 7. Detailed information on the significant causal relationship between obesity and stroke.

Intermediates	Outcomes	Methods	SNPs	P Value	Beta(95%CI)	Cochran's Q test		MR Egger	
						Q Value	P Value	Intercept	P Value
BMI	Stroke	MR Egger	433	4.10e-02	-0.004(-0.008/-0.0002)				
		Weighted median	433	8.13e-01	-0.0003(-0.003/0.002)				
		IVW	433	**1.70e-02**	0.0019(0.0003/0.003)	495.6	1.80e-02	0.0001	0.002
		Weighted mode	433	5.74e-01	-0.0015(-0.007/0.004)				
	Ischemic stroke	MR Egger	419	1.73e-03	0.267(0.101/0.434)				
		Weighted median	419	2.24e-05	0.215(0.116/0.314)				
		IVW	419	**1.88e-13**	0.227(0.166/0.314)	559.5	4.23e-06	-0.0008	0.606
		Weighted mode	419	1.37e-02	0.209(0.043/0.374)				
	Cardioembolic	MR Egger	430	4.90e-02	0.339(0.002/0.675)				
		Weighted median	430	6.60e-02	0.208(-0.013/0.430)				

		IVW	430	**3.50e-02**	0.132(0.009/0.254)	478.6	4.90e-02	-0.004	0.196
		Weighted mode	430	2.47e-01	0.220(-0.152/0.591)				
	Small vessel	MR Egger	396	1.28e-01	0.264(-0.075/0.602)				
		Weighted median	396	4.00e-02	0.194(0.009/0.379)				
		IVW	396	**1.90e-03**	0.191(0.070/0.311)	491.6	7.00e-04	-0.001	0.653
		Weighted mode	396	2.27e-01	0.233(-0.144/0.609)				
	Large artery	MR Egger	420	2.07e-02	0.459(0.072/0.846)				
		Weighted median	420	2.54e-04	0.415(0.193/0.638)				
		IVW	420	**3.17e-05**	0.299(0.158/0.440)	520.0	5.00e-04	-0.003	0.386
		Weighted mode	420	6.27e-02	0.391(-0.020/0.800)				
WHR	Stroke	MR Egger	30	0.501	-0.006(-0.024/0.011)				
		Weighted median	30	0.778	-0.001(-0.007/0.005)				
		IVW	30	0.742	0.001(-0.003/0.005)	23.67	0.745	0.0002	0.446
		Weighted mode	30	0.833	-0.001(-0.010/0.008)				
	Ischemic stroke	MR Egger	30	0.822	-0.075(-0.718/0.569)				
		Weighted median	30	0.050	0.205(0.0002/0.410)				
		IVW	30	**0.004**	0.209(0.066/0.352)	31.9	3.24e-01	0.007	0.383
		Weighted mode	30	0.341	0.185(-0.190/0.561)				
	Cardioembolic	MR Egger	30	0.764	-0.242(-1.805/1.322)				
		Weighted median	30	0.529	0.147(-0.310/0.603)				
		IVW	30	0.840	0.035(-0.306/0.376)	35.15	0.200	0.007	0.725
		Weighted mode	30	0.928	-0.034(-0.757/0.689)				
	Small vessel	MR Egger	30	0.328	0.736(-0.714/2.187)				
		Weighted median	30	0.014	0.531(0.108/0.954)				
		IVW	30	**0.001**	0.531(0.215/0.847)	38.93	1.03e-01	-0.005	0.778
		Weighted mode	30	0.142	0.560(-0.168/1.288)				
	Large artery	MR Egger	30	0.873	-0.139(-1.836/1.558)				
		Weighted median	30	0.340	0.245(-0.259/0.750)				
		IVW	30	0.228	0.229(-0.143/0.601)	37.59	0.132	0.009	0.667
		Weighted mode	30	0.498	0.357(-0.662/1.376)				

Supplementary Table 8. Detailed information on the significant causal relationship between dietary habits and lipoproteins.

Exposures	Intermediates	Methods	SNPS	P value	Beta(95%CI)	Cochran's Q test		MR-Egger	
						Q value	P value	Intercept	P value
Dried fruit intake	LDL-C	MR Egger	34	0.325	-0.248(-0.736/0.239)				
		Weighted median	34	0.003	-0.165(-0.272/-0.058)				
		IVW	34	**0.032**	-0.105(-0.201/-0.009)	66.38	0.0005	0.002	0.561
		Weighted mode	34	0.069	-0.210(-0.430/0.009)				
	HDL-C	MR Egger	35	0.049	-1.686(-3.307/-0.065)				
		Weighted median	35	4.0e-03	0.210(0.093/0.327)				
		IVW	35	0.856	-0.034(-0.401/0.333)	1233	4.1e-237	0.02	0.050
		Weighted mode	35	0.048	0.198(0.009/0.386)				
Oily fish intake	LDL-C	MR Egger	25	0.482	-0.353(-1.320/0.615)				
		Weighted median	25	0.060	0.104(-0.004/0.212)				
		IVW	25	0.455	-0.086(-0.312/0.140)	346.6	6.1e-59	0.004	0.584
		Weighted mode	25	0.118	0.135(-0.028/0.298)				
	HDL-C	MR Egger	52	0.759	0.088(-0.473/0.649)				
		Weighted median	52	0.009	0.111(0.028/0.194)				
		IVW	52	**0.039**	0.146(0.007/0.286)	580.1	8.2e-91	0.0009	0.834
		Weighted mode	52	0.707	0.029(-0.121/0.179)				
Cheese intake	LDL-C	MR Egger	31	0.001	0.877(0.393/1.362)				
		Weighted median	31	0.618	0.024(-0.071/0.120)				
		IVW	31	0.991	0.001(-0.112/0.113)	128.7	3.4e-14	-0.014	0.052
		Weighted mode	31	0.440	0.086(-0.129/0.301)				
	HDL-C	MR Egger	54	0.057	0.779(-0.004/1.563)				
		Weighted median	54	1.9e-06	0.171(0.093/0.249)				
		IVW	54	**0.049**	0.191(0.001/0.381)	1381	3.7e-254	-0.01	0.136
		Weighted mode	54	0.039	0.126(0.010/0.243)				
Cereal intake	LDL-C	MR Egger	17	0.128	4.634(-1.005/10.27)				
		Weighted median	17	0.520	-0.045(-0.182/0.092)				
		IVW	17	0.137	0.935(-0.296/2.167)	4093	0	-0.056	0.208

		Weighted mode	17	0.487	-0.054(-0.202/0.094)				
	HDL-C	MR Egger	34	0.877	0.138(-1.593/1.869)				
		Weighted median	34	0.681	-0.022(-0.126/0.082)				
		IVW	34	0.977	0.006(-0.401/0.413)	1825	0	-0.002	0.879
		Weighted mode	34	0.995	0.001(-0.153/0.154)				
Lamb/mutton intake	LDL-C	MR Egger	14	0.086	-6.741(-13.81/0.329)				
		Weighted median	14	0.700	0.034(-0.140/0.208)				
		IVW	14	**0.170**	-1.397(-3.394/0.600)	4121	0	0.066	0.150
		Weighted mode	14	0.762	0.037(-0.196/0.269)				
	HDL-C	MR Egger	26	0.046	2.213(0.149/4.279)				
		Weighted median	26	0.222	0.085(-0.051/0.221)				
		IVW	26	0.233	0.320(-0.206/0.845)	1071	4.0e-210	-0.022	0.076
		Weighted mode	26	0.525	0.082(-0.167/0.330)				
Hot drink temperature	LDL-C	MR Egger	35	0.388	-0.558(-1.807/0.691)				
		Weighted median	35	0.800	0.022(-0.151/0.196)				
		IVW	35	0.480	0.108(-0.191/0.408)	413.3	1.04e-66	0.006	0.289
		Weighted mode	35	0.193	-0.298(-0.737/0.141)				
	HDL-C	MR Egger	58	0.921	-0.045(-0.936/0.845)				
		Weighted median	58	7.2e-06	-0.249(-0.357/-0.140)				
		IVW	58	**0.048**	-0.204(-0.405/-0.002)	591	7.6e-90	-0.001	0.722
		Weighted mode	58	0.003	-0.362(-0.588/-0.136)				
Poultry intake	LDL-C	MR Egger	4	0.170	20.92(1.402/40.44)				
		Weighted median	4	0.339	-0.130(-0.396/0.136)				
		IVW	4	0.245	-0.624(-1.677/0.428)	85.74	1.8e-18	-0.237	0.163
		Weighted mode	4	0.428	-0.121(-0.381/0.139)				
	HDL-C	MR Egger	7	0.655	4.149(-13.52/21.82)				
		Weighted median	7	0.991	0.002(-0.285/0.288)				
		IVW	7	0.557	-0.168(-0.730/0.393)	94.29	3.9e-18	-0.047	0.652
		Weighted mode	7	0.406	0.225(-0.269/0.720)				
Salt added to food	LDL-C	MR Egger	47	0.763	-0.208(-1.552/1.135)				
		Weighted median	47	0.779	0.011(-0.069/0.092)				

		IVW	47	0.375	-0.222(-0.711/0.268)	4988	0	-0.0002	0.984
		Weighted mode	47	0.569	0.032(-0.078/0.142)				
	HDL-C	MR Egger	89	0.259	0.279(-0.202/0.759)				
		Weighted median	89	0.757	0.010(-0.053/0.073)				
		IVW	89	0.960	0.004(-0.150/0.158)	2014	0	-0.004	0.240
		Weighted mode	89	0.191	0.061(-0.030/0.151)				

Supplementary Table 9. Detailed information on the significant causal relationship between lipoproteins and stroke.

Exposures	Intermediates	Methods	SNPS	P value	Beta(95%CI)	Cochran's Q test		MR-Egger	
						Q value	P value	Intercept	P value
LDL-C	Stroke	MR Egger	177	1.00e-04	0.003(0.002/0.004)				
		Weighted median	177	2.00e-03	0.003(0.001/0.004)				
		IVW	177	**5.00e-02**	0.001(0.00/0.002)	205.9	0.061	-1.0e-04	0.053
		Weighted mode	177	8.00e-03	0.002(0.001/0.004)				
	Ischemic stroke	MR Egger	167	1.16e-03	0.154(0.063/0.245)				
		Weighted median	167	4.28e-04	0.126(0.056/0.196)				
		IVW	167	**2.38e-02**	0.082(0.011/0.153)	437.6	2.4e-26	-0.004	0.628
		Weighted mode	167	7.94e-06	0.142(0.082/0.203)				
	Cardioembolic	MR Egger	172	0.740	0.025(-0.124/0.175)				
		Weighted median	172	0.796	0.020(-0.135/0.176)				
		IVW	172	0.980	0.001(-0.113/0.116)	228.2	0.002	-0.001	0.627
		Weighted mode	172	0.715	0.025(-0.109/0.160)				
	Small vessel	MR Egger	141	0.200	0.102(-0.053/0.256)				
		Weighted median	141	0.246	0.080(-0.055/0.216)				
		IVW	141	0.454	0.045(-0.073/0.163)	226.5	5.2e-06	-0.003	0.267
		Weighted mode	141	0.210	0.075(-0.042/0.191)				
	Large artery	MR Egger	172	1.47e-04	0.398(0.197/0.598)				
		Weighted median	172	4.88e-05	0.359(0.186/0.532)				
		IVW	172	**3.04e-04**	0.281(0.129/0.434)	331.9	2.2e-12	-0.007	0.084
		Weighted mode	172	1.23e-06	0.376(0.230/0.523)				
HDL-C	Stroke	MR Egger	314	0.340	0.001(-0.001/0.003)				

	Weighted median	314	0.663	0.0004(-0.001/0.002)				
	IVW	314	0.093	-0.001(-0.002/0.003)	316.5	0.434	-6.9e-05	0.433
	Weighted mode	314	0.677	0.005(-0.002/0.003)				
Ischemic stroke	MR Egger	291	0.734	0.017(-0.080/0.113)				
	Weighted median	291	0.058	-0.074(-0.150/0.003)				
	IVW	291	**3.0e-04**	-0.107(-0.166/-0.049)	501.3	1.7e-13	-0.004	0.977
	Weighted mode	291	0.476	-0.031(-0.116/0.054)				
Cardioembolic	MR Egger	301	0.373	0.076(-0.090/0.242)				
	Weighted median	301	0.394	0.068(-0.089/0.226)				
	IVW	301	0.738	0.017(-0.083/0.118)	279.8	0.793	-0.002	0.387
	Weighted mode	301	0.314	0.105(-0.099/0.309)				
Small vessel	MR Egger	267	0.766	-0.025(-0.191/0.140)				
	Weighted median	267	0.004	-0.214(-0.361/-0.068)				
	IVW	267	**5.0e-05**	-0.207(-0.308/-0.107)	321.9	0.107	-0.006	0.797
	Weighted mode	267	0.021	-0.174(-0.322/-0.027)				
Large artery	MR Egger	296	0.404	0.082(-0.110/0.274)				
	Weighted median	296	0.882	0.014(-0.173/0.201)				
	IVW	296	**0.003**	-0.179(-0.298/-0.059)	372.2	0.002	-0.009	0.904
	Weighted mode	296	0.407	0.098(-0.134/0.330)				

Supplementary Figure 1. Scatter plots depicting the results of MR analysis investigating the association between dietary habits and stroke. Each line in the plot represents a different MR method, and the slope of each line represents the estimated association between the two variables. (A) Scatter plot between dried fruit intake and total stroke; (B) Scatter plot between oily fish intake and total stroke; (C) Scatter plot between cheese intake and ischemic stroke; (D) Scatter plot between dried fresh fruit intake and ischemic stroke; (E) Scatter plot between lamb/mutton intake and cardioembolic ischemic stroke; (F) Scatter plot between dried fruit intake and small vessel ischemic stroke; (G) Scatter plot between poultry intake and small vessel ischemic stroke; (H) Scatter plot between cereal intake and small vessel ischemic stroke; (I) Scatter plot between salt added to food and large artery ischemic stroke; (J) Scatter plot between hot drink temperature and large artery ischemic stroke. IVW, inverse-variance-weighted.

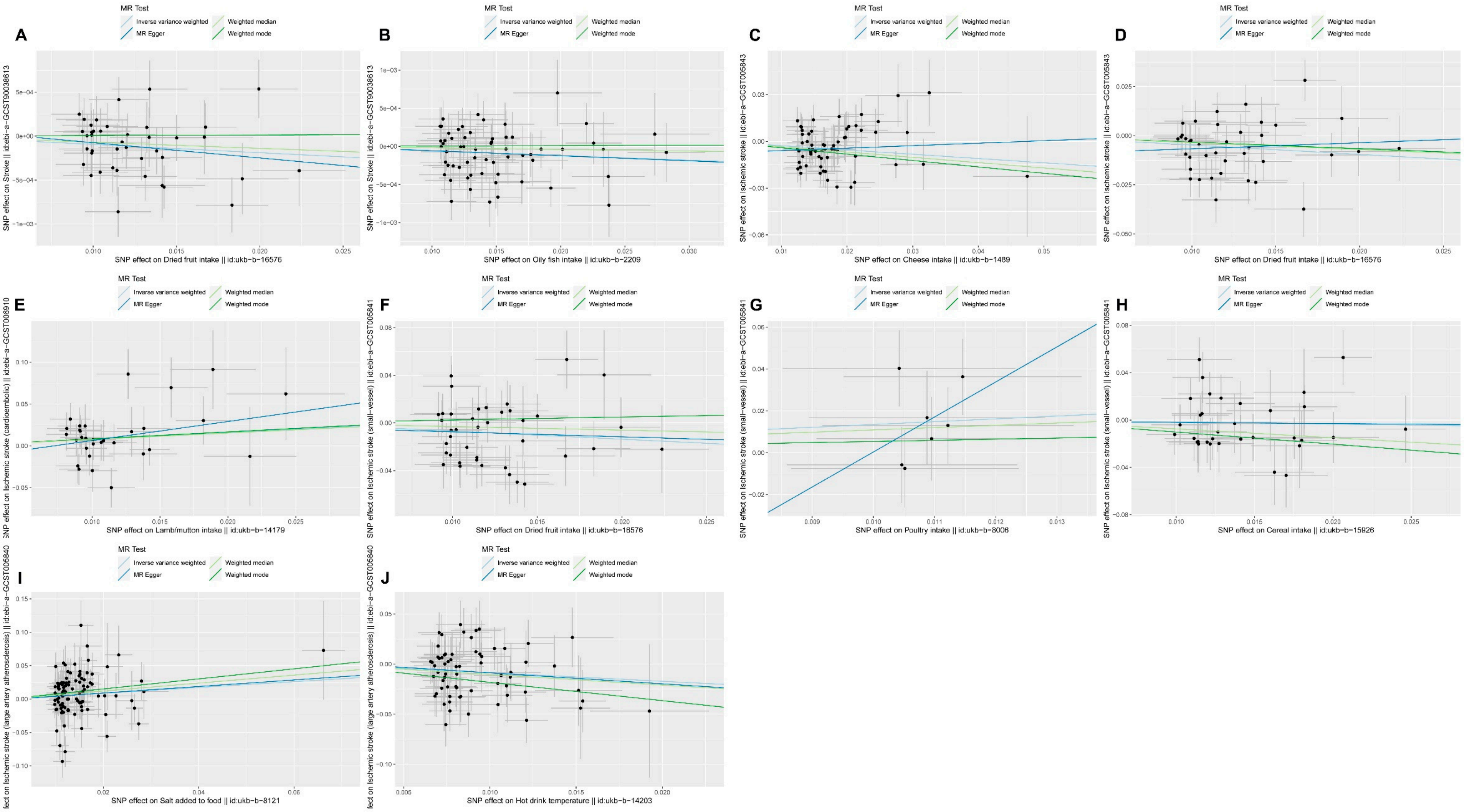

Supplementary Figure 2. Funnel plot depicting the significant results of MR analysis investigating the association between dietary habits and stroke. Funnel plot represented the estimates of precision (1/SE) and Wald ratios for each SNP. (A) Funnel plot for dried fruit intake and total stroke; (B) Funnel plot for oily fish intake and total stroke; (C) Funnel plot for cheese intake and ischemic stroke; (D) Funnel plot for dried fresh fruit intake and ischemic stroke; (E) Funnel plot for lamb/mutton intake and cardioembolic ischemic stroke; (F) Funnel plot for dried fruit intake and small vessel ischemic stroke; (G) Funnel plot for poultry intake

and small vessel ischemic stroke; (H) Funnel plot for cereal intake and small vessel ischemic stroke; (I) Funnel plot for salt added to food and large artery ischemic stroke; (J) Funnel plot for hot drink temperature and large artery ischemic stroke. IVW, inverse-variance-weighted.

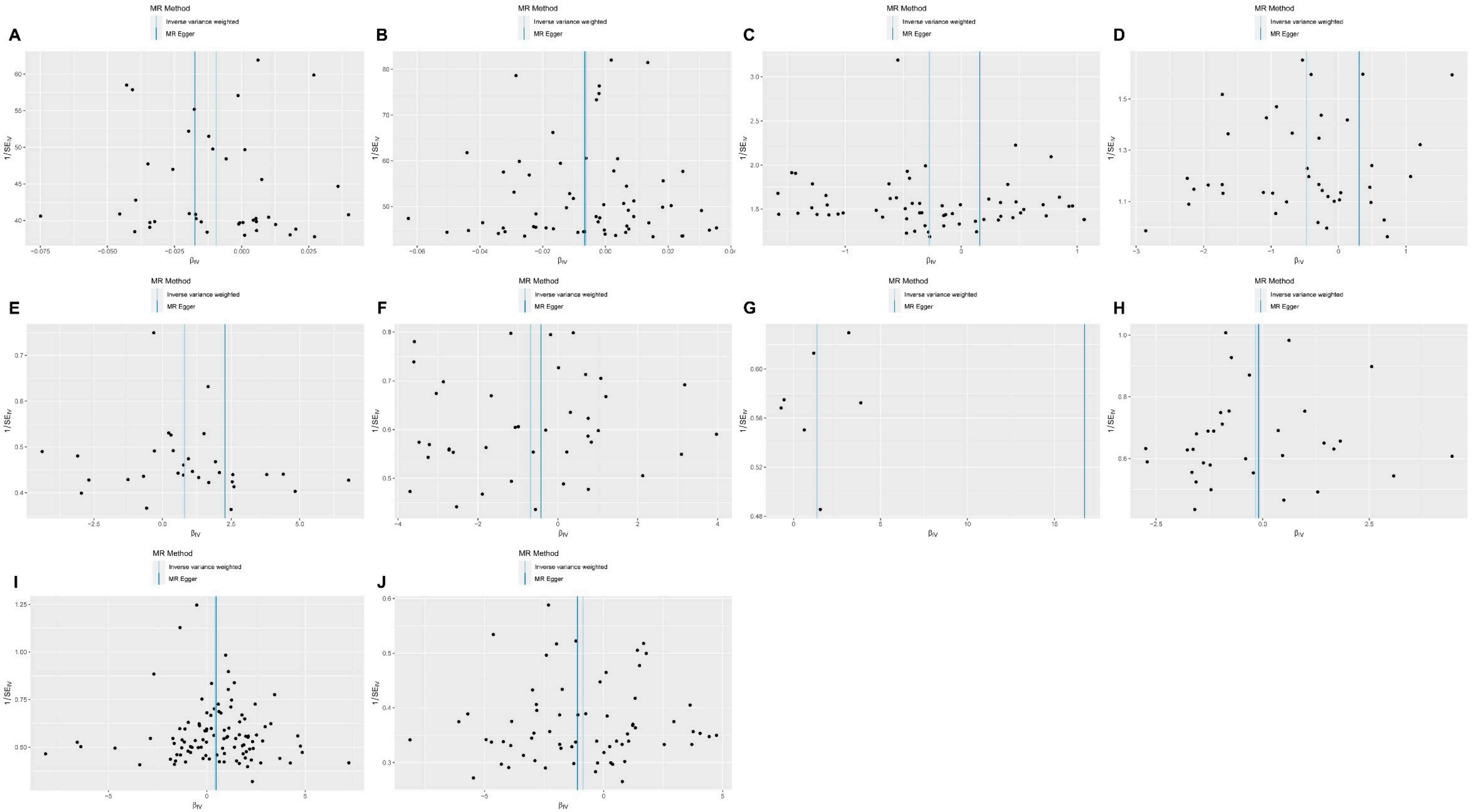

Supplementary Figure 3. Leave-one-out plots depicting the significant associations between dietary habits and stroke. Each black line in the figure corresponds to the outcome of the MR analysis when one single nucleotide polymorphism (SNP) is removed from the analysis, while the remaining SNPs are used on the left. (A) Leave-one-out analysis between dried fruit intake and total stroke; (B) Leave-one-out analysis between oily fish intake and total stroke; (C) Leave-one-out analysis between cheese intake and ischemic stroke; (D) Leave-one-out analysis between dried fresh fruit intake and ischemic stroke; (E) Leave-one-out analysis between lamb/mutton intake and cardioembolic ischemic stroke; (F) Leave-one-out analysis between dried fruit intake and small vessel ischemic stroke; (G) Leave-one-out analysis between poultry intake and small vessel ischemic stroke; (H) Leave-one-out analysis between cereal intake and small vessel ischemic stroke; (I) Leave-one-out analysis between salt added to food and large artery ischemic stroke; (J) Leave-one-out analysis between hot drink temperature and large artery ischemic stroke. Red line reference the overall effect.

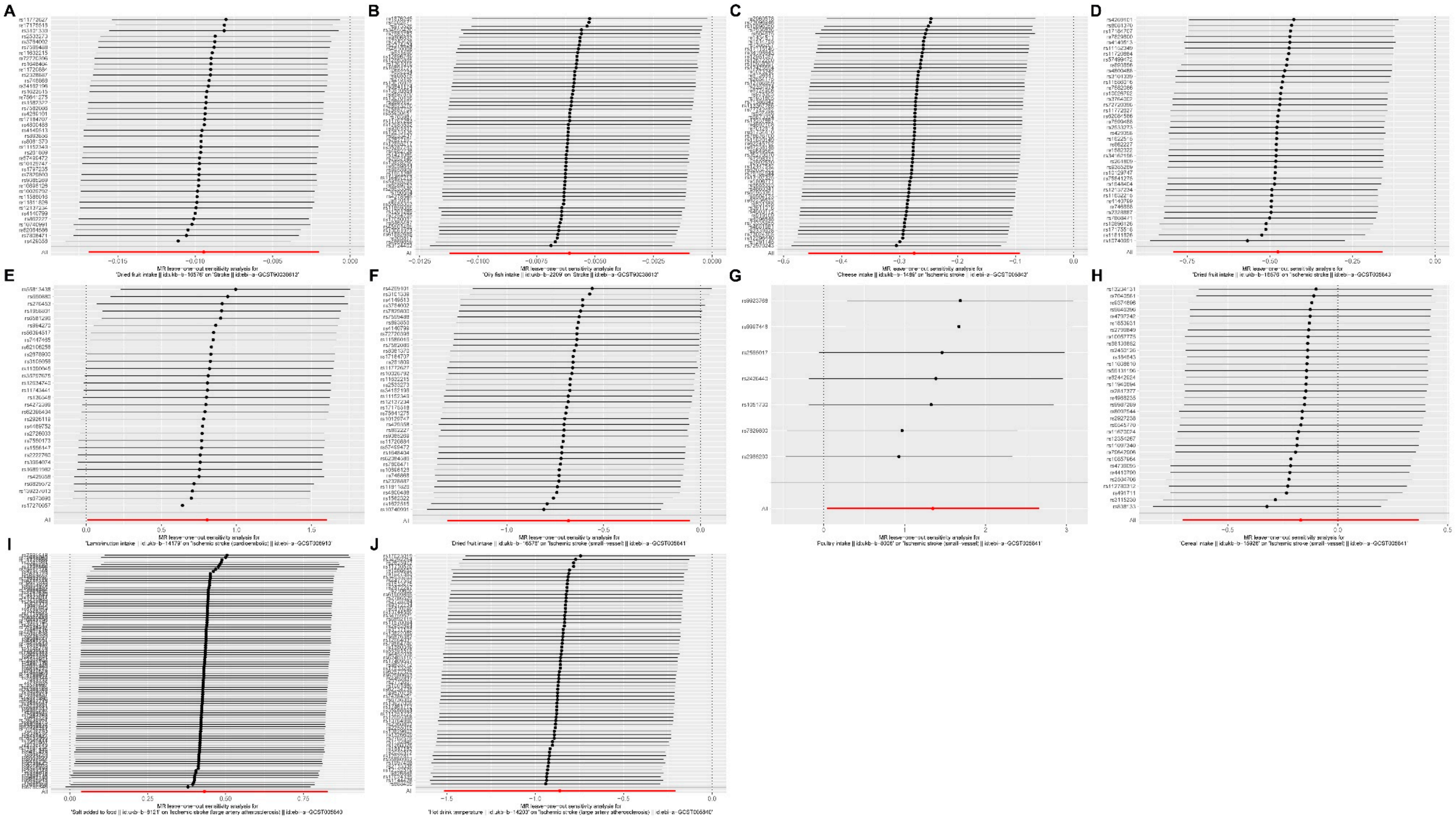

Supplement: Supplementary file 1 [file nutrients-16-03548-s001.zip › nutrients-3242617-supplementary.pdf]
